# Preparation and Synthetic Applications of Phototropone

**DOI:** 10.1021/acs.joc.3c00590

**Published:** 2023-06-21

**Authors:** Jack P. Lowe, Nathan R. Halcovitch, Susannah C. Coote

**Affiliations:** Department of Chemistry, Lancaster University, Bailrigg, LA1 4YB, U.K.

## Abstract

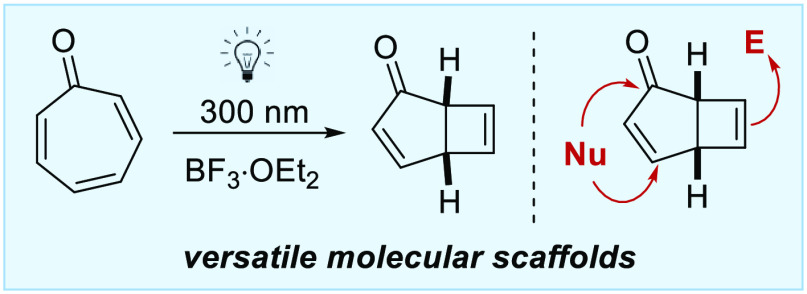

An optimized multigram-scale route to phototropone (bicyclo[3.2.0]hepta-2,6-dien-7-one) is reported via the 4-π-photocyclization of tropone complexed to Lewis acid. Phototropone is a highly versatile molecular building block, and its conversion into 18 novel derivatives using standard transformations is demonstrated, allowing access to a variety of rigid bicyclic scaffolds.

The development of novel molecular building blocks to access unexplored areas of biologically relevant chemical space remains a pertinent area of organic chemistry.^1^ In particular, diversity-oriented synthesis (DOS)^2^ has emerged as a powerful tool in generating highly diverse chemical libraries for biological activity screening.^3^ Such an approach often starts from a single core molecule that possesses functionality that can be exploited to generate a wide range of novel building blocks.^4^ Boasting both cyclobutene and cyclopentenone motifs, we envisaged phototropone (**1**; bicyclo[3.2.0]heptadienone) as a versatile synthetic intermediate en route to such complex chemical libraries. Despite its rich synthetic potential, the use of **1** as a scaffold has not previously been explored, but its rigid nature appears promising for the introduction of diversity^3^ (for example, skeletal diversity through addition of extra rings/groups, functional diversity through selective functionalizations of the cyclobutene and enone moieties). Herein, we describe an improved multigram-scale synthesis of **1**, as well as various selective manipulations of **1** to give diverse building blocks with synthetic handles for further manipulation in varied applications.

First, we sought an efficient synthetic route for the preparation of **1**. Prinzbach and co-workers^5^ first reported the thermal rearrangement of tropovalene (**2**) to **1** in quantitative yield (Scheme 1), though the inefficient synthesis of **2** renders this an impractical route to **1**. Similarly, Story and Fahrenholtz described the rearrangement of quadricyclanone (**3**) into **1**, but **3** is also difficult to access.^6^ Alternatively, Cavazza and Zandomeneghi reported the [2 + 2] photocycloaddition of enone **4** with acetylene, followed by acetoxy elimination to give **1** in moderate overall yield.^7^ This route is not ideal when working on a multigram scale due to safety issues associated with employing large quantities of acetylene gas and because enone **4** is not commercially available. Given our interest in 4-π-photocyclization reactions,^8,9^ a particularly attractive approach to **1** would involve the 4-π-photocyclization of tropone (**5**), which would allow direct access to **1** in a single step from commercially available materials. The 4-π-photocyclization of tropolone (2-hydroxytropone) and tropolone ethers are well-known;^9^ thus it is somewhat surprising that the corresponding photocyclization of tropone itself has been investigated only sporadically. Thus, while the irradiation of tropone in acetonitrile results only in very slow dimerization reactions (furnishing the [6 + 4], [6 + 2], [4 + 2],^10^ and [6 + 6]^11^ dimers in low yields), early work by Childs and Taguchi,^12^ and Reingold and co-workers,^13^ showed that irradiation of tropone in acidic media produces **1**, albeit in very low yields. Notably, Cavazza et al. demonstrated that irradiation of **5** in the presence of boron trifluoride generated **1** selectively, although the reaction was only demonstrated on very small scale (<1 mmol).^14^ It was suggested that the lowest excited state of protonated tropone (or tropone complexed to a Lewis acid) corresponds to a π–π* transition that enables productive 4-π-photocyclization, whereas in the absence of acid, the lowest excited state of tropone corresponds to a prohibited n−π* transition from which 4- π-photocyclization does not occur.^15^ Inspired by the efficiency of Cavazza’s approach, we optimized this process to access **1** on a multigram scale for downstream applications.

**Scheme 1:**
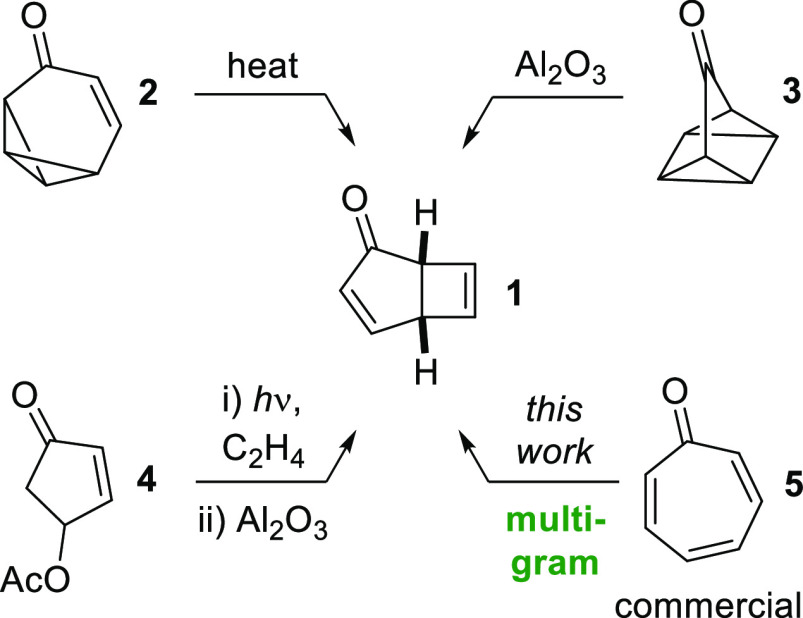
Synthetic Routes to Phototropone (**1**)

We began our investigation by performing the 4-π-photocyclization of **5** in a Rayonet batch reactor using Cavazza’s conditions^14^ (Table 1, entry 1). Thus, irradiation of a 25 mM solution of **5**·BF_3_ in acetonitrile at 300 nm led directly to **1** after 2 h. However, only traces of **1** were isolated from the reaction despite full conversion being observed by ^1^H NMR spectroscopy. It turned out that **1** was much more volatile than **5**, leading to difficulty in its isolation; thus, a lower-boiling solvent was required to minimize coevaporation of **1**. The **5**·BF_3_ complex was insoluble in diethyl ether, *tert-*butyl methyl ether, and methanol but soluble in dichloromethane, so this solvent was chosen for further optimization. **1** was obtained in good yield after 2 h of irradiation at 300 nm (entry 2), whereas irradiation at 350 or 419 nm only led to traces of phototropone after 18 h of irradiation (entries 3–4), due to the lower absorption of **5**·BF_3_ at these wavelengths. Other Lewis acid additives such as AlCl_3_ and Sc(OTf)_3_ were also investigated (entries 5–6), but they gave significantly poorer results than BF_3_·OEt_2_. Finally, the concentration of **5**·BF_3_ was varied. The yield of **1** only increased marginally at a lower concentration (10 mM; entry 8) and decreased significantly at higher concentrations of **5** (50 or 100 mM; entries 9 and 10), with the longer reaction times resulting in more degradation. Therefore, a compromise between isolated yield and throughput was chosen, and irradiation in dichloromethane at 25 mM was selected as the optimal batch conditions, giving around 3 g of **1** in a typical run, with yields between runs ranging from 64% to 75%).

**Table 1:**
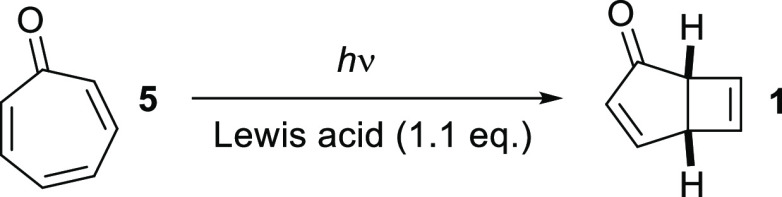
Optimization of the 4-π-Photocyclization of **5**

Entry	Solvent	λ (nm)	Lewis Acid	Concn (mM)	Time (h)^a^	Yield (%)^b^
1	MeCN	300	BF_3_·OEt_2_	25	2	traces
2	CH_2_Cl_2_	300	BF_3_·OEt_2_	25	2	61
3	CH_2_Cl_2_	350	BF_3_·OEt_2_	25	18	traces
4	CH_2_Cl_2_	419	BF_3_·OEt_2_	25	24	0
5	CH_2_Cl_2_	300	AlCl_3_	25	4	7
6	CH_2_Cl_2_	300	Sc(OTf)_3_	25	18	0
7	CH_2_Cl_2_	300	BF_3_·OEt_2_	10	1.5	66
8	CH_2_Cl_2_	300	BF_3_·OEt_2_	50	4.5	48
9	CH_2_Cl_2_	300	BF_3_·OEt_2_	100	6	41

a Time taken for complete consumption of **5**

b Isolated yields after chromatography.

With multigram quantities of **1** in hand, attention was turned to its transformation into novel building blocks, to produce a library of diverse functionalized products. First, additions to the cyclopentenone motif were targeted (Scheme 2). Conjugate addition of dimethylmalonate gave ketone **6**, Corey–Chaykovsky cyclopropanation gave ketone **7**, and conjugate reduction using LiAlH(OtBu)_3_ gave ketone **8**.^16^ Reduction of **1** using DIBAL-H was completely regioselective and reasonably diastereoselective, leading to a 3:1 mixture of allylic alcohols **9** and *epi*-**9**. Interestingly, Luche reduction conditions led to lower diastereoselectivity in this reaction, and employing sodium borohydride alone led to a complex mixture of products comprising both direct and conjugate reduction products. In contrast, the addition of vinylmagnesium bromide was completely regioselective and completely diastereoselective, leading to a single product isomer **10**, although **10** was unstable toward purification on silica/alumina, and derivatization before purification is advisable. Accordingly, tertiary alcohol **10** underwent oxidative 1,3-transposition upon exposure to pyridinium dichromate (PDC) to give substituted enone **11**.^17^ It should be noted that (as for **1** itself) many of the derivatives described in Scheme 2 are volatile, and care should be taken during handling/storage to avoid losses.

**Scheme 2:**
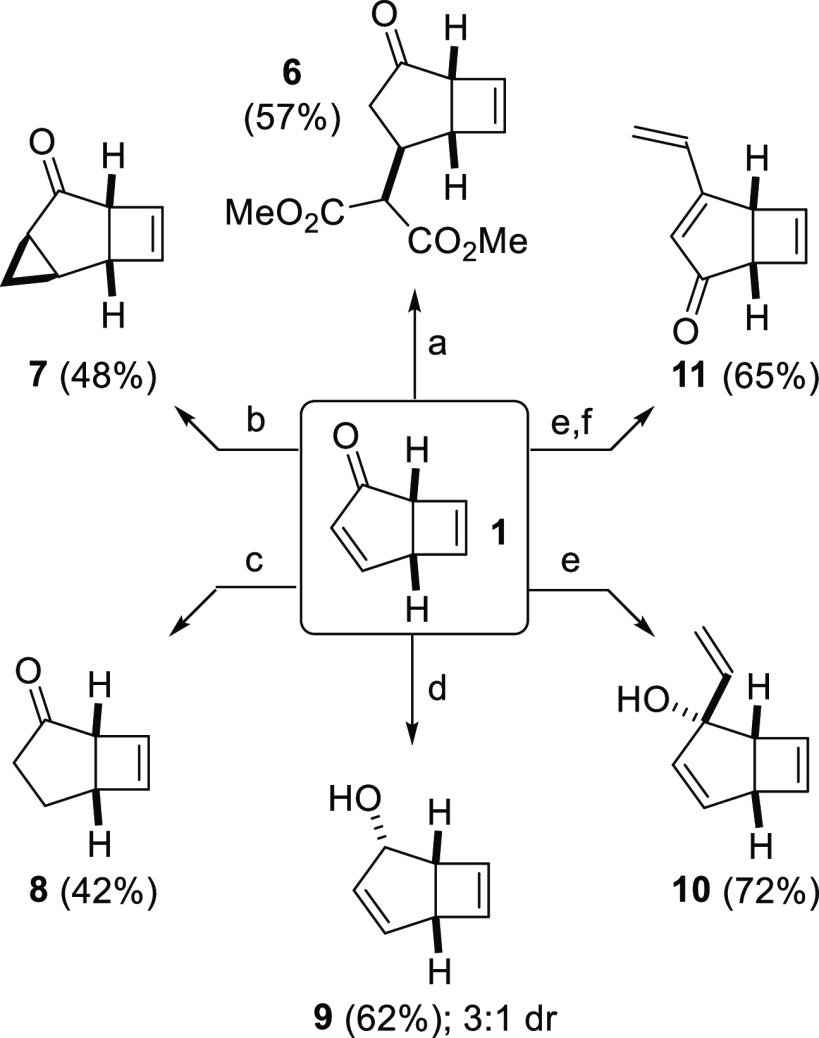
Additions to Phototropone To Give Varied Bicyclic Building Blocks Reaction conditions: Dimethyl malonate, MeONa, MeOH; Me_3_S(O)I, NaH, DMSO; LiAlH(O^*t*^Bu)_3_, THF; (^*i*^Bu)_2_AlH, THF; VinylMgBr, THF; Pyridinium dichromate, CH_2_Cl_2_.

Next, various functionalizations of cyclopentenone were studied (Scheme 3). Thus, iodination of **1** cleanly furnished **12**, which is a novel cross-coupling partner that allows the subsequent introduction of various groups to the enone moiety. For example, Suzuki coupling using phenylboronic acid gave phenylphototropone **13**, and Sonogashira coupling of trimethylsilylacetylene resulted in alkynylphototropone **14**. Baylis–Hillman reaction of **1** generated alcohol **15**, and copper-catalyzed aziridination using PhI=NTs was selective for the enone rather than the cyclobutene, giving ketoaziridine **16** in moderate yield (the addition of extra equivalents of PhI=NTs resulted in complex mixtures in which reaction also took place on the cyclobutene, although the bis-aziridine that would result from double addition could not be isolated). Similarly, nucleophilic epoxidation conditions furnished the corresponding epoxyketone **17**, which is also a versatile synthetic intermediate for further derivatization. For example, **17** underwent a nucleophilic ring opening/elimination sequence with morpholine to give aminophototropone **18**, and similar reactions with other nucleophiles could easily be envisaged. Epoxyketone **17** also undergoes Wharton transposition^18^ upon exposure to hydrazine under acidic conditions, generating *epi*-**9**—a diastereoisomer of the major product obtained via direct reduction of **1** using DIBAL-H.

**Scheme 3:**
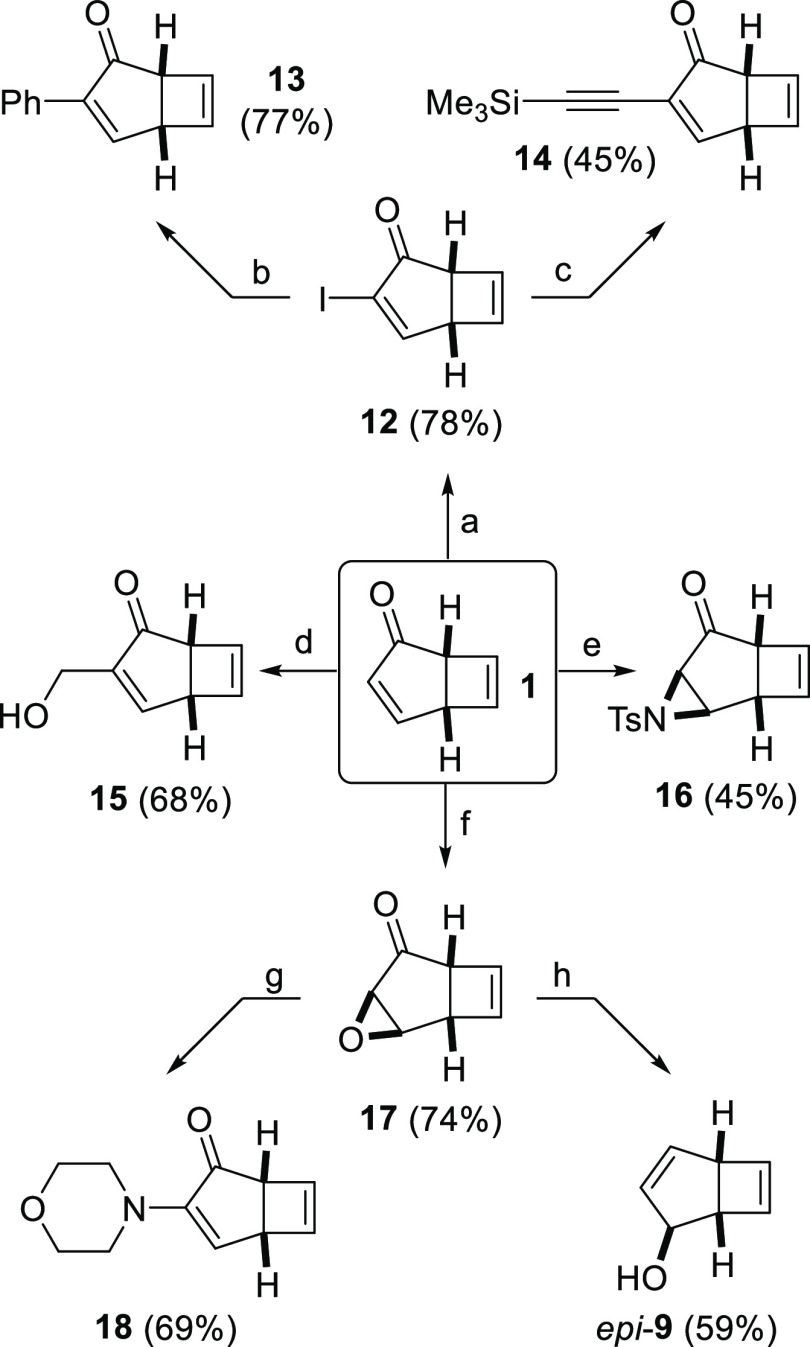
Functionalization of Phototropone and Selected Further Manipulations of Iodide **12** and Epoxyketone **17** Reaction conditions: I_2_, DMAP, K_2_CO_3_, THF/H_2_O; PhB(OH)_2_, Pd(PPh_3_)_4_, Cs_2_CO_3_, THF/H_2_O; Me_3_Si–C≡CH, PdCl_2_(PPh_3_)_2_, CuI, THF; CH_2_O (aq), ^n^Bu_3_P, CHCl_3_/MeOH; PhI = NTs, Cu(OTf)_2_, MeCN; H_2_O_2_ (aq), NaOH (aq), MeOH; Morpholine, 70 °C, MeOH/H_2_O; H_2_N-NH_2_·H_2_O, Et_3_N, MeOH.

Manipulation of the cyclobutene moiety proved more challenging than expected; attempts at dihydroxylation and RuO_4_-mediated oxidative cleavage led to complex mixtures, and attempts at aziridination/cyclopropanation led to reaction only on the enone moiety by using a variety of reagent systems. Nevertheless, epoxidation using *m*-CPBA proceeded cleanly to give epoxide **19** as a single diastereomer; however, the fragility of **19** limited the isolated yields after chromatographic purification, and further derivatization of the crude product is recommended. In addition, an inverse-electron-demand Diels–Alder cycloaddition with tetraphenylcyclopentadienone furnished cycloadduct **20** (Scheme 4), the structure of which was confirmed through analysis by X-ray diffraction (see SI for details).

**Scheme 4:**
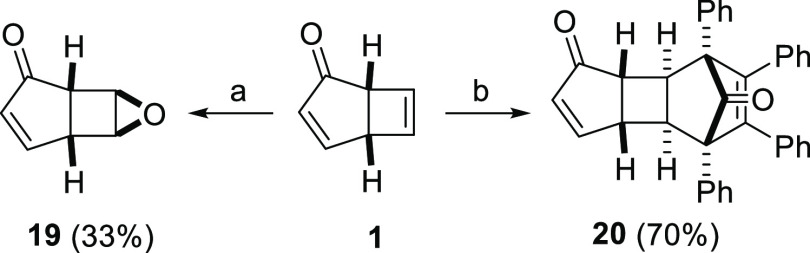
Derivatization of the Cyclobutene Moiety of **1** Reaction conditions: *meta*-Chloroperbenzoic acid, CH_2_Cl_2_; Tetraphenylcyclopentadienone, PhMe, reflux.

Finally, phototropone undergoes a variety of other cycloaddition reactions, allowing access to diverse molecular frameworks (Scheme 5). For example, a Diels–Alder reaction with the Danishefsky diene took place on the cyclopentenone, giving tricycle **21** after acid-catalyzed elimination. Similarly, 1,3-dipolar cycloaddition led to tricyclic pyrrolidine **22**, and cyclopropanation with dichlorocarbene gave dichlorocyclopropane **23**.

**Scheme 5:**
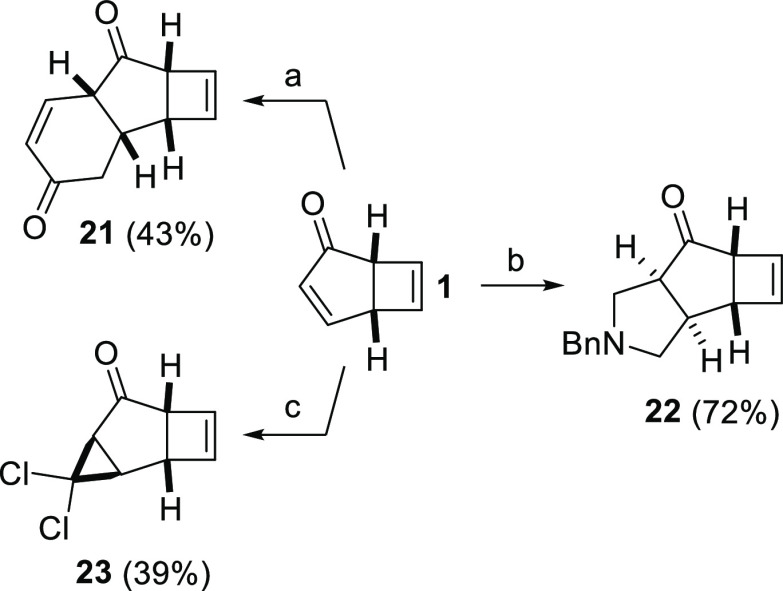
Cycloadditions Involving Phototropone Reaction conditions: Danishefsky diene, BF_3_·OEt_2_, CH_2_Cl_2_; CF_3_CO_2_H, MeOCH_2_N(Bn)CH_2_SiMe_3_, CH_2_Cl_2_; CHCl_3_, NaOH, ^*n*^Bu_4_NCl.

In summary, we have developed an efficient, multigram-scale route to phototropone (**1**), via 4-π-photocyclization of tropone (**5**) in the presence of a Lewis acid. **1** is an interesting scaffold that was shown to be amenable to a wide variety of synthetic transformations, including chemoselective epoxidations and aziridinations, regioselective reduction/Grignard additions, and various cycloadditions. The products obtained are novel molecular building blocks, many of which can themselves undergo further synthetic transformations and which have broad potential uses in medicinal and synthetic chemistry. In total, 18 new small molecule targets have been accessed, showcasing the potential of 4-π-photocyclization as a method for the generation of promising rigid scaffolds for further synthetic manipulations. The extension of this work to the 4-π-photocyclization of substituted tropones and further diversifications of the resulting phototropone cores will be reported in due course.

## Data Availability

The data underlying this study are available in the published article and its Supporting Information.

## Supporting Information Available

Experimental procedures and analytical and crystallographic data for all new compounds (PDF)
