# Supplementary material for: Preparation and Synthetic Applications of Phototropone
Source: J Org Chem. 2023 Jun 21;88(13):9514–7. doi: 10.1021/acs.joc.3c00590 (PMC10337036; doi:10.1021/acs.joc.3c00590)
Supplement: Supplementary file 1 — jo3c00590_si_001.pdf [file jo3c00590_si_001.pdf]

Preparation and Synthetic Applications of Phototropone

Supporting Information

Jack P. Lowe, Nathan R. Halcovitch and Susannah C. Coote*

Email: s.coote@lancaster.ac.uk

Lancaster University, Bailrigg, Lancaster, LA1 4YB, UK

1	Synthetic Procedures and Analytical Data for New Compounds	S2
	1.1 General Information	S2
	1.2 Synthetic Procedures/Data	S3
2	Crystallographic Data for **20**	S16
3	References	S17
4	$^{1}H/^{13}C$ NMR Spectra of New Compounds	S18

1.1 General Information

All solvents and reagents were purchased from Sigma Aldrich, Acros Organics, TCI Chemicals, Fisher Scientific, Alfa Aesar or Fluorochem. All reactions were carried out under dry inert conditions unless stated otherwise, and anhydrous solvents were purchased from Acros Organics equipped with AcroSeal™. All other solvents used were of reagent grade. Reaction vessels were oven-dried and cooled under an argon atmosphere prior to use, and experiments were performed under argon gas.

Photochemical reactions were carried out in a Rayonet RPR-100 Photochemical Batch photoreactor equipped with 16 × 8 W fluorescent lamps (300 nm, 350 nm or 429 nm), in Duran phototubes (70 mL tubes for scale-up), which were placed in the carousel 4 cm from the light source. For optimisation reactions, 20 mL Duran phototubes were used, which were placed in the carousel 2 cm from the light source. The carousel was not rotated during operation. Reactions were monitored by thin layer chromatography (TLC) or by ^{1}H NMR spectroscopy (with solvent suppression). Under the optimised conditions, the emission is centred at 300 nm – the spectral energy distribution (revised 5/9/2017) is reproduced below as provided by the manufacturer (The Southern New England Ultraviolet Company). When operating, the approximate temperature inside the reactor chamber is 40 °C.

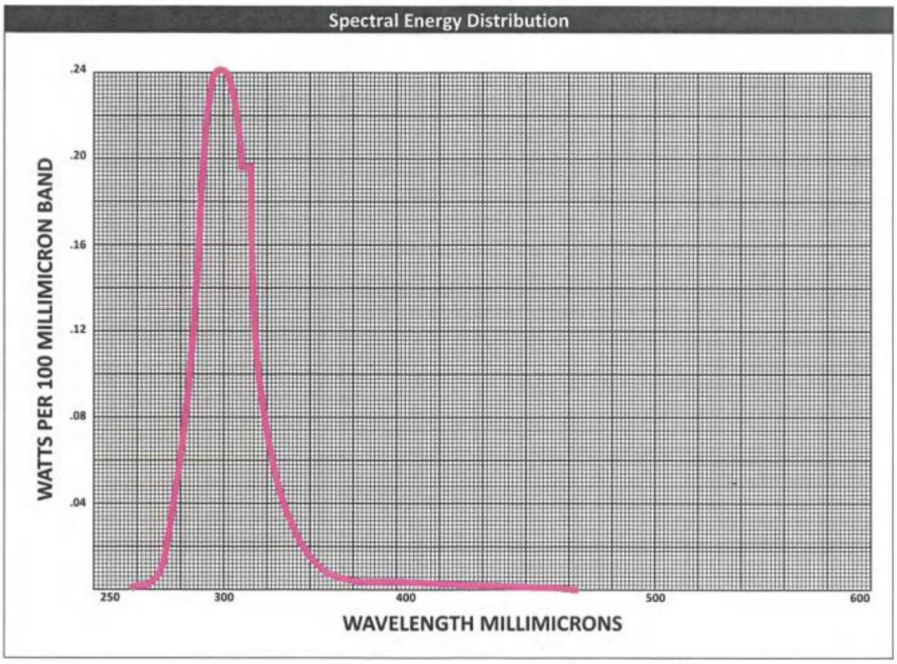

Analytical TLC was carried out on pre-coated aluminium-backed TLC plates (silica gel 60 F_{254}) and visualised by UV light (254 nm) or stained with potassium permanganate as developing agent. Flash chromatography on silica gel was performed with silica gel from VWR (40-63 microns).

^{1}H and ^{13}C NMR spectroscopic data were collected on a Bruker Ultrashield 400 Plus instrument and chemical shifts are reported in parts per million (δ) calibrated to residual $CHCl_3$ (δ_H = 7.26 ppm) and $CDCl_3$ (δ_C 77.0 ppm, central line). The following abbreviations are used to describe the multiplicities: s = singlet, d = doublet, t = triplet, q = quartet, m = multiplet and br = broad. Coupling constants (*J*) are given in Hertz. Assignment of the signals in ^{1}H and ^{13}C NMR spectra was achieved using 2D-NMR techniques (COSY, HSQC and HMBC). When using ^{1}H NMR to monitor the reaction progress, solvent suppression was used to supress the signal arising from the non-deuterated solvent. Infrared (IR) spectra were recorded on an Agilent Technologies Cary 630 FTIR spectrometer. High resolution mass spectrometry data were recorded using electron spray ionization (ESI) or atmospheric pressure chemical ionization (APCI) on a Shimadzu LCMS-IT-TOF mass spectrometer. Melting points were performed on a Sanyo Gallenkamp capillary melting point apparatus and are uncorrected.

1.2 Synthetic Procedures and Analytical Data for new Compounds

Bicyclo[3.2.0]hepta-3,6-dien-2-one 1

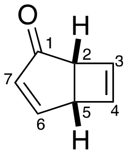

A solution of tropone (1.9 mL, 17.6 mmol) and $BF_3{\cdot}OEt_2$ (2.4 mL, 19.5 mmol) in anhydrous CH_2Cl_2 (390 mL) was distributed between six 70-mL Duran phototubes, and each tube was bubbled with Ar gas for 10 minutes before being irradiated at 300 nm for 10 hours. This process was repeated with a further six phototubes, then the tube contents were combined and the solvent was removed under reduced pressure (500 mbar) to yield the crude product. Purification by flash chromatography on silica gel with $CH_2Cl_2 \rightarrow$ 95:5 CH_2Cl_2-Et_2O as the eluent gave **1** (3.1 g, 2.07 mmol, 75%) as a yellow oil.

R_f (CH_2Cl_2) = 0.47.

^{1}H NMR (400 MHz, $CDCl_3$); δ 7.64 (dd, *J* = 5.8, 2.6 Hz, 1H), 6.53 (dd, *J* = 2.6, 0.9 Hz, 1H), 6.34 (dd, *J* = 2.6, 1.3 Hz, 1H), 6.06 (dd, *J* = 5.8, 0.9 Hz, 1H), 3.93 (dd, *J* = 3.5, 2.6, 1.4 Hz, 1H), 3.48 – 3.41 (m).

^{13}C{^{1}H} NMR (101 MHz, $CDCl_3$); δ 206.3, 162.6, 143.1, 137.0, 134.5, 53.2, 50.6.

FTIR (ATR) *v* (cm^{-1}): 3054, 2946, 1690 (C=O).

HRMS (APCI): *m/z* calculated for C_7H_6O $[M+H]^+$ 107.0491, found 107.0489.

NB The product is volatile and should not be subjected to high vacuum.

***rac*-1,3-Dimethyl 2-[(*1R,2S,5R*)-4-oxobicyclo[3.2.0]hept-6-en-2-yl]propanedioate 6**

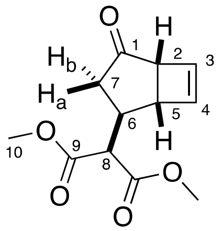

Dimethyl malonate (0.17 mL, 1.5 mmol) was added to a stirred solution of sodium methoxide (82 mg, 1.5 mmol) in MeOH (1 mL) at 0 °C, and the resulting mixture was allowed to warm to room temperature and stirred at room temperature for one hour. A solution of **1** (106 mg, 1.00 mmol) in MeOH (1 mL) was added dropwise at 0 °C and the mixture was allowed to warm to room temperature and then stirred at room temperature for 15 hours. The reaction mixture was quenched with brine (5 mL) and extracted with Et_2O (3 x 10 mL). The combined organic layers were dried ($MgSO_4$) and evaporated under reduced pressure to give the crude product. Purification by flash column

chromatography on silica gel with 19:1 → 9:1 heptane-EtOAc as the eluent gave **6** (128 mg, 0.57 mmol, 57%) as a yellow oil.

R_f (Heptane-EtOAc, 4:1) = 0.25

^{1}H NMR (400 MHz, $CDCl_3$); δ 6.37 (dt, *J* = 2.6, 0.8 Hz, 1H), 6.12 (dd, *J* = 2.6, 1.0 Hz, 1H), 3.72 (s, 3H), 3.71 (s, 3H) 3.44 (d, *J* = 6.7 Hz, 1H), 3.31 (ddd, *J* = 2.7, 1.8, 1.0 Hz, 1H), 3.27 (dd, *J* = 3.0, 0.8 Hz, 1H), 3.20 (ddd, *J* = 18.7, 9.2, 1.2 Hz, 1H), 2.83 (dddd, *J* = 9.1, 6.7, 1.5, 0.9 Hz, 1H), 2.23 – 2.15 (dtd, *J* = 18.4, 1.6, 0.9 Hz 1H).

$^{13}C\{^1H\}$ NMR (101 MHz, $CDCl_3$); δ 214.2, 168.70, 168.67, 143.0, 137.2, 55.3, 55.0, 52.8, 52.7, 48.3, 39.2, 33.9.

FTIR (ATR) *v* (cm^{-1}): 3449, 2959, 1718 (C=O stretches).

HRMS (APCI): *m/z* calculated for $C_{12}H_{14}O_5$ $[M+H]^+$ 239.0914, found 239.0906.

rac-(_1R,2R,4R,6R_)-Tricyclo[4.2.0.0^{2,4}]oct-7-en-5-one 7

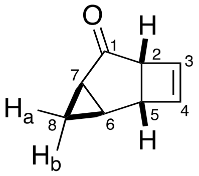

Sodium hydride (80 mg of a 60% dispersion in mineral oil, 2.00 mmol) was added portion-wise to anhydrous DMSO (1 mL) at room temperature, followed by trimethylsulfoxonium iodide (440 mg, 2.00 mmol). The resulting suspension was stirred at room temperature for one hour, before a solution of **1** (212 mg, 2 mmol) in anhydrous DMSO (0.2 mL) was added. The resulting suspension was stirred at 50 °C (using a metal heating block) for 1 hour, then allowed to cool to room temperature. Crushed ice was added (~5 g) and the resulting mixture was extracted with Et_2O (3 x 20 mL). The combined organic layers were dried ($MgSO_4$) and evaporated under reduced pressure to yield **7** (115 mg, 0.96 mmol, 48%) as a yellow oil.

R_f (4:1 Heptane-EtOAc) = 0.29

^{1}H NMR (400 MHz, $CDCl_3$); δ 6.35 (d, *J* = 2.5 Hz, 1H), 6.12 (dd, *J* = 2.5, 1.1 Hz, 1H), 3.29 (t, *J* = 2.4 Hz, 1H), 3.19 (m, 1H), 2.06 – 2.01 (m, 1H), 1.91 (dddd, *J* = 9.0, 4.6, 3.4, 2.3 Hz, 1H), 1.34 – 1.22 (m, 1H), 0.70 (td, *J* = 4.8, 3.4 Hz, 1H).

$^{13}C\{^1H\}$ NMR (101 MHz, $CDCl_3$); δ 209.9, 142.3, 136.5, 52.1, 46.4, 27.6, 23.5, 12.1.

FTIR (ATR) *v* (cm^{-1}): 3049, 2926, 2994, 1707 (C=O).

HRMS (APCI): *m/z* calculated for C_8H_8O $[M+H]^+$ 121.0648, found 121.0652.

NB The product is volatile and should not be subjected to high vacuum.

rac-(1R,2S)-Bicyclo[3.2.0]hept-6-en-2-one 8

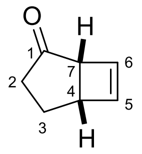

Lithium tri-*tert*-butoxyaluminium hydride (610 mg, 2.4 mmol) was added portionwise at 0 °C to a stirred solution of phototropone (212 mg, 2.0 mmol) in THF (5 mL). The resulting suspension was stirred at room temperature for 16 h, then water (20 mL) was added. The resulting mixture was extracted with CH_2Cl_2 (5 x 5 mL), dried ($MgSO_4$) and evaporated under reduced pressure (200 mbar, with water bath at ambient temperature) to give the crude product. Purification by flash column chromatography on silica gel with CH_2Cl_2 as the eluent gave **8** (91 mg, 0.84 mmol, 72%) as a colourless oil.

R_f (CH_2Cl_2) = 0.41

^{1}H NMR (400 MHz, $CDCl_3$); δ 6.22 (d, *J* = 2.6 Hz, 1H), 6.12 (dd, *J* = 2.6, 0.9 Hz, 1H), 3.49-3.46 (m, 1H), 3.20-3.18 (m, 1H), 3.00-2.90 (m, 1H), 2.19-2.11 (m, 1H), 1.95-1.84 (m, 1H).

^{13}C{^{1}H} NMR (101 MHz, $CDCl_3$); δ 217.2, 143.3, 136.7, 54.4, 44.6, 34.4, 21.8.

The spectroscopic data are consistent with those previously reported.[1]

rac-(1R,2S,5R)-Bicyclo[3.2.0]hepta-3,6-dien-2-ol 9

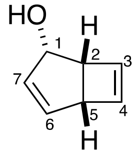

DIBAL-H (0.5 mL of a 1.0 M solution in THF, 1.00 mmol) was added dropwise to a solution of **1** (53 mg, 0.5 mmol) in Et_2O (1 mL) at 0 °C, and the resulting mixture was stirred at 0 °C for 15 minutes. Distilled water (5 mL) was then added dropwise at 0 °C and the reaction mixture was extracted with diethyl ether (3 x 10 mL). The combined organic layers were dried ($MgSO_4$) and evaporated under reduced pressure to give a 3:1 mixture (by ^{1}H NMR spectroscopy) of **9** and *epi*-**9** (34 mg, 0.31 mmol, 62%) as a colourless oil. Partial separation of the diastereoisomers by column chromatography on silica gel with 9:1 pentane-CH_2Cl_2 as the eluent afforded an analytically pure sample of **9** (8 mg, 0.072 mmol, 14%) as a colourless oil.

Data for **9**:

R_f (pentane-CH_2Cl_2, 1:1) = 0.24

^{1}H NMR (400 MHz, $CDCl_3$); δ 6.64 (d, *J* = 2.7 Hz, 1H), 6.18 (t, *J* = 2.5 Hz, 1H), 6.10 (ddd, *J* = 5.7, 2.4, 1.4 Hz, 1H), 5.63 (dd, *J* = 5.7, 1.8 Hz, 1H), 4.80 (d, *J* = 9.0 Hz, 1H), 3.68 (dd, *J* = 9.0, 3.4 Hz, 1H), 3.56 (t, *J* = 2.8 Hz, 1H).

$^{13}C\{^{1}H\}$ NMR (101 MHz, $CDCl_3$); δ 148.9, 137.2, 136.5, 134.4, 74.2, 54.5, 48.7.

FTIR (ATR) *ν* (cm^{-1}): 3386 (OH), 3045, 2931, 2864, 1695.

rac-(_1R,2S,5R_)-2-Ethenylbicyclo[3.2.0]hepta-3,6-dien-2-ol 10

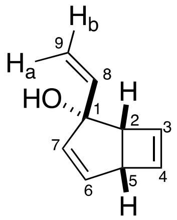

A solution of vinylmagnesium bromide (1.7 mL of a 0.89 M solution in THF) was added dropwise to a solution of **1** (106 mg, 1.00 mmol) in THF (5 mL) at 0 °C, and the resulting mixture was stirred at room temperature for 12 hours. A 0.5 M aqueous solution of HCl (10 mL) was added, and the reaction mixture was extracted with diethyl ether (3 x 10 mL). The combined organic layers were dried ($MgSO_4$) and evaporated under reduced pressure to yield the crude product. Purification by flash column chromatography on silica gel with CH_2Cl_2 then 95:5 CH_2Cl_2-Et_2O as the eluent gave **10** (96 mg, 0.72 mmol, 72%) as a colourless oil.

R_f (CH_2Cl_2) = 0.33

^{1}H NMR (400 MHz, $CDCl_3$); δ 6.63 (dd, *J* = 2.7, 0.7 Hz, 1H), 6.19 (d, *J* = 2.4 Hz, 1H), 6.09 (dd, *J* = 5.6, 2.4 Hz, 1H), 6.02 (dd, *J* = 17.2, 10.8, 1H), 5.46 (dd, *J* = 5.6, 0.7 Hz, 1H), 5.15 (dd, *J* = 17.2, 1.2, 1H), 5.05 (dd, *J* = 10.8, 1.6, 1H) 3.65 – 3.62 (m, 1H), 3.43 (dt, *J* = 3.3, 1.0 Hz, 1H).

$^{13}C\{^{1}H\}$ NMR (101 MHz, $CDCl_3$); δ 148.3, 143.6, 137.1, 136.4, 135.9, 112.3, 81.9, 54.6, 54.1.

FTIR (ATR) *ν* (cm^{-1}): 3416 (O-H, broad), 3050, 2924, 2853, 2359, 2338, 1720.

HRMS (APCI): *m/z* calculated for $C_9H_{10}O$ $[M+H]^+$ 135.0804, found 135.0805.

NB Tertiary alcohol **10** was found to be somewhat unstable towards purification on silica/alumina, resulting in some degradation during the purification process. Further derivatization of the crude product is advisable prior to purification.

4-Ethenylbicyclo[3.2.0]hepta-3,6-dien-2-one 11

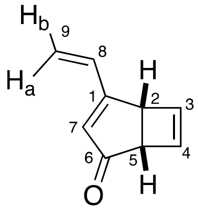

A solution of vinylmagnesium bromide (1.7 mL of a 0.89 M solution in THF) was added dropwise to a solution of **1** (106 mg, 1.00 mmol) in THF (5 mL) at 0 °C, and the resulting mixture was stirred at room temperature for 12 hours. A 0.5 M aqueous solution of HCl (10 mL) was added, and the reaction mixture was extracted with diethyl ether (3 x 10 mL). The combined organic layers were dried ($MgSO_4$) and evaporated under reduced pressure to yield the crude tertiary alcohol product. PDC (752 mg, 2.00 mmol) was added in one portion to a solution of the crude tertiary alcohol product in CH_2Cl_2 (5 mL), and the resulting suspension was stirred at room temperature for 12 hours, then diluted with diethyl ether (5 mL), filtered through Celite and evaporated under reduced pressure to give the crude product. Purification by flash column chromatography on silica gel with CH_2Cl_2 as the eluent gave **11** (86 mg, 0.65 mmol, 65%) as a colourless oil.

R_f (CH_2Cl_2) = 0.31

^{1}H NMR (400 MHz, $CDCl_3$); δ 6.75 (ddt, *J* = 17.4, 10.6, 0.6 Hz, 1H), 6.60 (dt, *J* = 2.4, 0.7 Hz, 1H), 6.41 (dd, *J* = 2.4, 1.5 Hz, 1H), 6.00-5.99 (m, 1H), 5.82 (dt, *J* = 17.4, 0.7 Hz, 1H), 5.60 (br d, *J* = 10.6, 1H), 4.03 (dt, *J* = 2.7, 0.6, 1H), 3.62-3.61 (m, 1H).

^{13}C{^{1}H} NMR (101 MHz, $CDCl_3$); δ 205.0, 170.6, 142.2, 138.4, 132.0, 131.1, 123.2, 54.4, 48.2.

FTIR (ATR) *v* (cm^{-1}): 3051, 2928, 1680 (C=O), 1622, 1562.

HRMS (APCI): *m/z* calculated for C_9H_8O [M+H]$^+$ 133.0648, found 133.0650.

3-Iodobicyclo[3.2.0]hepta-3,6-dien-1-one 12

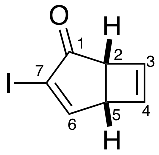

A solution of **1** (318 mg, 3.00 mmol), K_2CO_3 (621 mg, 4.5 mmol), DMAP (72 mg, 0.59 mmol) and I_2 (1.1 g, 4.5 mmol)) in 1:1 THF-H_2O (30 mL) was stirred at room temperature for 1.5 hours. EtOAc (20 mL) was added and the organic layer was seperated. The organic layer was washed with 1M HCl (10 mL) and saturated aquous $Na_2S_2O_3$ (2 x 10 mL). The organic layer was dried ($MgSO_4$) and evaporated under reduced pressure to give the crude product. Purification by flash column chromatography on silica gel with 4:1 hexane-EtOAc as the eluent gave **12** (498 mg, 2.2 mmol, 78%) as an orange oil.

R_f (hexane-EtOAc, 4:1) = 0.38.

^{1}H NMR (400 MHz, $CDCl_3$); δ 8.01 (d, *J* = 3.0 Hz, 1H), 6.59 (ddd, *J* = 1.6, 0.9, 0.9 Hz 1H), 6.34 (dd, *J* = 1.6, 0.9 Hz, 1H), 4.00 (ddt, *J* = 3.0, 2.5, 0.9 Hz, 1H), 3.61 (ddt, *J* = 2.5, 1.5, 0.9 Hz, 1H).

^{13}C{^{1}H} NMR (101 MHz, $CDCl_3$); δ 199.6, 167.8, 143.6, 137.0, 104.0, 52.3, 49.8.

FTIR (ATR) *ν* (cm^{-1}): 2988, 2359, 1707 (C=O).

HRMS (APCI): *m/z* calculated for C_7H_5OI $[M+H]^+$ 232.9458, found 232.9451.

3-Phenylbicyclo[3.2.0]hepta-3,6-dien-1-one 13

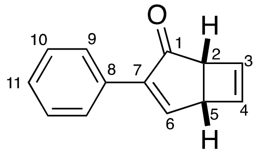

A solution of **12** (231 mg, 1.00 mmol), $PhB(OH)_2$ (242 mg, 2.0 mmol), Cs_2CO_3 (1.3 g, 4.00 mmol) and $Pd(PPh_3)_4$ (57 mg, 0.05 mmol) in THF:H_2O (9:1, 7.5 mL) was stirred at 75 °C (using a metal heating block) for 18 hours. After cooling to room temperature, brine (5 mL) was added, and the mixture was extracted with EtOAc (3 x 10 mL). The organic layer was dried ($MgSO_4$) and evaporated under reduced pressure to give the crude product. Purification by flash column chromatography on silica gel with 9:1 heptane-EtOAc as the eluent gave **13** (140 mg, 0.77 mmol, 77%) as an orange oil.

R_f (hexane-EtOAc, 9:1) = 0.33.

^{1}H NMR (400 MHz, $CDCl_3$); δ 7.76 (d, *J* = 3.0 Hz, 1H), 7.68-7.63 (m, 2H), 7.41-7.29 (m, 3H), 6.61 (dd, 1H, *J* = 2.4, 0.5), 6.44 (dd, *J* = 2.4, 1.3 Hz, 1H), 3.95 (ddd, *J* = 3.0, 2.5, 0.5 Hz, 1H), 3.69 (dd, *J* = 2.5, 1.3, 0.5 Hz, 1H)

^{13}C{^{1}H} NMR (101 MHz, $CDCl_3$); δ 204.0, 156.1, 143.4, 143.2, 137.2, 132.2, 128.6, 128.5, 127.4, 54.9, 47.4.

FTIR (ATR) *ν* (cm^{-1}): 3052, 2935, 1692 (C=O)

HRMS (APCI): *m/z* calculated for $C_{13}H_{10}O$ $[M+H]^+$ 183.0804, found 183.0807.

3-[2-(Trimethylsilyl)ethynyl]bicyclo[3.2.0]hepta-3,6-dien-2-one 14

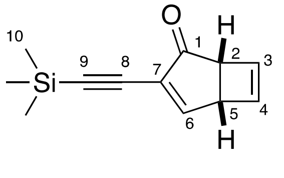

A solution of **12** (131 mg, 0.56 mmol), trimethylsilylacetylene (95 mL 0.67 mmol), CuI (11 mg, 0.056 mmol) and $PdCl_2(PPh_3)_2$ (20 mg, 0.028 mmol) in THF (2.5 mL) was stirred at 0 °C for one hour. The

solution was allowed to warm to room temperature before 1M HCl (2 mL) was added, and the resulting mixture was extracted with EtOAc (3 x 5 mL). The combined organic layers were dried ($MgSO_4$) and evaporated under reduced pressure to give the crude product. Purification by flash column chromatography on silica gel with 4:1 heptane-EtOAc as the eluent gave **14** (52 mg, 0.25 mmol, 45%) as a waxy orange solid.

R_f (heptane-EtOAc, 4:1) = 0.32

^{1}H NMR (400 MHz, $CDCl_3$); δ 7.74 (d, *J* = 3.0 Hz, 1H), 6.53-6.51 (m, 1H), 6.34 (dd, *J* = 2.4, 1.3 Hz, 1H), 3.93-3.88 (m, 1H), 3.55 (dd, *J* = 2.1, 1.3 Hz, 1H), 0.22 (s, 9H).

^{13}C{^{1}H} NMR (101 MHz, $CDCl_3$); δ 201.5, 163.7, 143.0, 137.0, 129.9, 102.1, 95.7, 53.2, 48.4, -0.08.

FTIR (ATR) *v* (cm^{-1}): 3190, 3054, 2957, 2898, 2154 (C≡C), 1705 (C=O).

HRMS (APCI): *m/z* calculated for $C_{12}H_{14}OSi$ $[M+H]^+$ 203.0887, found 203.0878.

3-(Hydroxymethyl)bicyclo[3.2.0]hepta-3,6-dien-2-one 15

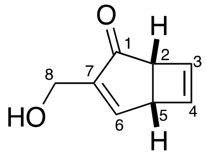

Formaldehyde (0.12 mL of a 37% (wt) aqueous solution, 1.50 mmol) and $^nBu_3P$ (1 drop) were added sequentially to a stirred solution of **1** (106 mg, 1.00 mmol) in $CHCl_3$ (2 mL) and MeOH (2 mL) at room temperature. The resulting solution was stirred for 1 hour at room temperature, then evaporated under reduced pressure to give the crude product. Purification by flash column chromatography on silica gel with 8:2 $CHCl_3$-Et_2O as the eluent gave **15** (92 mg, 0.68 mmol, 68%) as pale yellow oil.

R_f ($CHCl_3$-Et_2O, 8:2) = 0.08.

^{1}H NMR (400 MHz, $CDCl_3$); δ 7.49 (dt, *J* = 3.0, 1.3 Hz, 1H), 6.54 (dt, *J* = 2.4, 1.0 Hz, 1H), 6.33 (dd, *J* = 2.4, 1.2 Hz, 1H), 4.32 (dt, *J* = 14.4, 1.3 Hz, 1H), 4.26 (dt, *J* = 14.4, 1.3 Hz, 1H), 3.90-3.88 (m, 1H), 3.55-3.54 (m, 1H), 2.69 (br s, 1H, OH).

^{13}C{^{1}H} NMR (101 MHz, $CDCl_3$); δ 205.8, 156.3, 144.9, 143.1, 136.3, 57.7, 54.2, 48.3.

FTIR (ATR) *v* (cm^{-1}): 3382 (OH), 3052, 2928, 2868, 1676 (C=O).

HRMS (APCI): *m/z* calculated for $C_8H_8O_2$ $[M+H]^+$ 137.0597, found 137.0603.

***rac*-(*1S,2R,4R,6R*)-3-(4-Methylbenzenesulfonyl)-3-azatricyclo[4.2.0.0^{2,4}]oct-7-en-5-one 16**

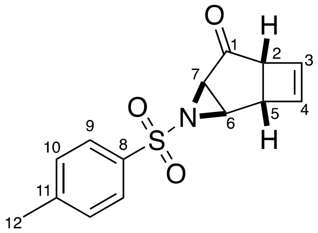

PhI=NTs (243 mg, 0.91 mmol) was added in one portion to a stirred solution of **1** (25 mg, 0.23 mmol) and $Cu(OTf)_2$ (6 mg, 0.023 mmol) in MeCN (2 mL) at room temperature under nitrogen, then stirred at room temperature for 48 hours. The reaction mixture was filtered through Celite, washed with diethyl ether (15 mL) and evaporated under reduced pressure to give the crude product. Purification by flash column chromatography on silica gel with 4:1 hexane-EtOAc as the eluent gave **16** (29 mg, 0.11 mmol, 45%) as a light beige oil.

R_f (Hexane-EtOAc, 4:1) = 0.15

^{1}H NMR (400 MHz, $CDCl_3$); δ 7.81 (d, *J* = 8.1 Hz, 2H), 7.35 (d, *J* = 8.1 Hz, 2H), 6.30 (dd, *J* = 2.7, 1.1 Hz, 1H), 6.09 (dd, *J* = 2.7, 1.1 Hz, 1H), 3.77 (dd, *J* = 4.4, 1.1 Hz, 1H), 3.51 (dd, *J* = 2.6, 1.1 Hz, 1H), 3.38 (dd, *J* = 4.4, 1.1 Hz, 1H), 3.31 (dt, *J* = 2.6, 1.1 Hz, 1H), 2.45 (s, 3H).

^{13}C{^{1}H} NMR (101 MHz, $CDCl_3$); δ 200.8, 145.4, 139.7, 137.2, 134.4, 130.1, 128.1, 52.7, 46.4, 45.6, 45.2, 21.8.

FTIR (ATR) *v* (cm^{-1}): 3125, 3058, 2957, 1742 (C=O).

HRMS (APCI): *m/z* calculated for $C_{14}H_{13}NO_3S$ $[M+H]^+$ 276.0689, found 276.0687.

***rac*-(*1S,2R,4R,6R*)-3-Oxatricyclo[4.2.0.0^{2,4}]oct-7-en-5-one 17**

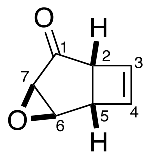

An aqueous 2M solution of NaOH (1.5 mL, 3.00 mmol) was added dropwise to a stirred solution of **1** (318 mg, 3.00 mmol) and H_2O_2 (50% w/w in H_2O, 0.73 mL, 8.97 mmol) in MeOH (50 mL) at −20 °C under argon, and the resulting solution was stirred at −20 °C for 15 minutes. The reaction mixture was diluted with H_2O (30 mL) and extracted with CH_2Cl_2 (3 x 20 mL). The combined organic layers were dried ($MgSO_4$) and evaporated under reduced pressure to give epoxide **17** (272 mg, 2.23 mmol, 74%) as a colourless oil.

R_f (CH_2Cl_2) = 0.63.

^{1}H NMR (400 MHz, $CDCl_3$); δ 6.31 (dd, *J* = 2.6, 0.8 Hz, 1H), 6.14 (dd, *J* = 2.6, 1.5 Hz, 1H), 3.89 (td, *J* = 1.4, 0.5 Hz, 1H), 3.57 (ddd, *J* = 2.6, 1.4, 0.8 Hz, 1H), 3.41 (td, *J* = 1.4, 0.5 Hz, 1H), 3.34 (ddd, *J* = 2.6, 1.5, 0.5 Hz, 1H).

$^{13}C\{^{1}H\}$ NMR (101 MHz, $CDCl_3$); δ 203.6, 139.1, 137.2, 58.9, 55.2, 50.9, 46.0.

FTIR (ATR) *v* (cm^{-1}): 3423, 2920, 2851, 1727 (C=O).

3-(Morpholin-4-yl)bicyclo[3.2.0]hepta-3,6-dien-2-one 18

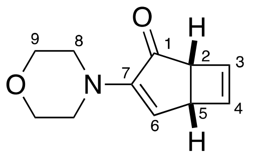

A solution of **17** (122 mg, 1.00 mmol) and morpholine (86 mL, 1.2 mmol) in 3:1 MeOH:H_2O (2 mL) was stirred at 70 °C (using a metal heating block) for four hours. The reaction mixture was diluted with brine (5 mL) and extracted with Et_2O (3 x 5mL). The combined organic layers were dried ($MgSO_4$) and evaporated under reduced pressure to give **18** (57 mg, 0.29 mmol, 69%) as light-yellow crystals.

R_f (heptane-EtOAc, 4:1) = 0.14

m.p. = 59-61 °C

^{1}H NMR (400 MHz, $CDCl_3$); δ 6.58 (dt, *J* = 2.4, 0.6, 1H), 6.39 (d, *J* = 3.0 Hz, 1H), 6.35 (dd, *J* = 2.4, 1.3 Hz, 1H), 3.80 (ddd, *J* = 6.0, 3.7, 2.3 Hz, 4H), 3.76 (t, *J* = 3.0 Hz, 1H), 3.54 (dd, *J* = 2.2, 1.2 Hz, 1H), 3.30-3.22 (m, 2H), 2.99-2.91 (m, 2H).

$^{13}C\{^{1}H\}$ NMR (101 MHz, $CDCl_3$); δ 202.3, 150.9, 144.8, 136.1, 132.1, 66.7, 53.8, 48.3, 44.9.

FTIR (ATR) *v* (cm^{-1}): 3050, 2967, 2860, 2816, 1686 (C=O).

HRMS (ESI): *m/z* calculated for $C_{11}H_{13}NO_2$ $[M+H]^+$ 192.1019, found 192.1012.

***rac*-(*1R,2R,5R*)-Bicyclo[3.2.0]hepta-3,6-dien-2-ol *epi*-9**

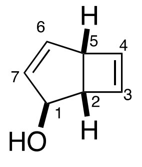

Triethylamine (0.14 mL, 1.00 mmol) was added to a stirred solution of hydrazine monohydrate (0.13 mL, 1.00 mmol) in MeCN (2.5 mL) at room temperature, and the resulting solution was stirred at room temperature for 20 minutes. A solution of **17** (94 mg, 0.76 mmol) in acetonitrile (2.5 mL) was added in one portion at room temperature and the resulting solution stirred for a further 20 minutes. Glacial acetic acid (0.065 mL, 1.14 mmol) was then added at room temperature and the solution was stirred

for a further five minutes. The reaction mixture was diluted with water (5 mL) and was extracted with Et_2O (3 x 10 mL). The combined organic layers were dried ($MgSO_4$) and evaporated under reduced pressure to yield the crude product. Purification by flash column chromatography on silica gel with 1:1 pentane-CH_2Cl_2 as the eluent gave *epi*-**9** (48 mg, 0.44 mmol, 59%) as a colourless oil.

R_f (pentane-CH_2Cl_2, 1:1) = 0.24

^{1}H NMR (400 MHz, $CDCl_3$); δ 6.47 (dd, *J* = 2.6, 1.0 Hz, 1H), 6.21 – 6.19 (m, 2H), 5.84 – 5.78 (m, 1H), 4.58 (s, 1H), 3.82 (qd, *J* = 3.1, 1.3, 1H), 3.18 (dt, *J* = 4.1, 1.0 1H).

$^{13}C\{^1H\}$ NMR (101 MHz, $CDCl_3$): δ 148.5, 139.8, 138.6, 133.8, 76.2, 55.1, 55.0.

FTIR (ATR) *v* (cm^{-1}): 3343 (br, OH), 3047, 2924.

***rac*-(*1R,2S,4R,5S*)-3-Oxatricyclo[3.3.0.0^{2,4}]oct-7-en-6-one 19**

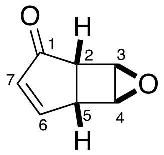

m-CPBA (516 mg, 2.31 mmol) was added dropwise to a stirred solution of **1** (106 mg, 1.00 mmol) in dry dichloromethane (2 mL) at 0 °C under argon, and the resulting suspension was allowed to warm to room temperature. The reaction mixture was stirred at room temperature for 24 hours before being diluted with saturated aqueous $NaHCO_3$ (2 mL) and extracted with CH_2Cl_2 (3 x 5 mL). The combined organic layers were dried ($MgSO_4$) and evaporated under reduced pressure to give the crude product. Purification by flash column chromatography on silica gel with 4:1:0.01 pentane-CH_2Cl_2-NEt_3 as the eluent gave **19** (40 mg, 0.33 mmol, 33%) as a colourless oil.

R_f (Pentane-CH_2Cl_2, 4:1) = 0.19.

^{1}H NMR (400 MHz, $CDCl_3$); δ 7.60 (dd, *J* = 5.8, 2.9, 1H), 6.39 (dt, *J* = 5.8, 0.7, 1H), 4.10 (dd, *J* = 2.9, 2.1 Hz, 1H), 3.93 (dd, *J* = 2.9, 2.1 Hz, 1H), 3.47 (qd, *J* = 2.9, 0.7 Hz, 1H), 3.01 (t, *J* = 2.9 Hz, 1H).

$^{13}C\{^1H\}$ NMR (101 MHz, $CDCl_3$); δ 204.6, 160.1, 138.6, 60.5, 55.9, 54.5, 52.4.

FTIR (ATR) *v* (cm^{-1}): 3060, 2959, 1764, 1697 (C=O).

rac-(2S,3S,7S,8S)-1,9,10,11-Tetraphenyltetracyclo[7.2.1.0^{2,8}.0^{3,7}]dodeca-5,10-diene-4,12-dione 20

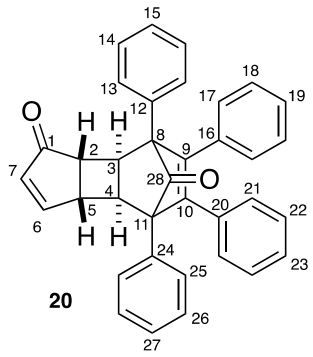

20

Tetraphenylcyclopentadienone (192 mg, 0.50 mmol) was added in one portion to a stirred solution of **1** (53 mg, 0.50 mmol) in PhMe (2 mL) at room temperature under argon. The resulting solution was heated at 100 °C (using a metal heating block) for 18 hours, then allowed to cool to room temperature ad evaporated under reduced pressure to give the crude product. Purification by flash column chromatography on silica gel with 1:1 $CHCl_3$-CH_2Cl_2 then CH_2Cl_2 as the eluent gave **20** (172 mg, 0.35 mmol, 70%) as a white solid.

R_f (CH_2Cl_2) = 0.41

m.p. = 188-190 °C

^{1}H NMR (400 MHz, $CDCl_3$); δ 7.92 (dd, *J* = 5.5, 3.2 Hz, 2H), 7.47-7.37 (m, 8H), 7.34-7.26 (m, 2H), 7.06-6.92 (m, 6H), 6.79-6.72 (m, 4H), 6.48 (dd, *J* = 5.5, 0.7 Hz, 1H), 3.27-3.18 (m, 3H), 2.82 (dd, *J* = 4.9, 2.7 Hz, 1H).

^{13}C{^{1}H} NMR (101 MHz, $CDCl_3$); δ 206.4, 198.2, 162.3, 141.3, 141.1, 139.9, 134.3, 133.9, 133.3, 132.9, 130.6, 130.5, 130.4, 129.5, 128.5, 128.1, 127.9, 127.6, 127.5, 127.4, 127.2, 127.1, 67.0, 65.7, 49.0, 47.0.

FTIR (ATR) *v* (cm^{-1}): 3052, 3030, 2939, 2957, 1780 (C=O), 1696 (C=O), 1575.

HRMS (APCI): *m/z* calculated for $C_{35}H_{24}O$ $[M+H]^+$ 461.1900, found 461.1908.

2aH,2bH,3H,4H,6aH,7H,7aH-Cyclobuta[a]indene-4,7-dione 21

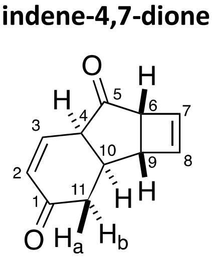

To a solution of **1** (106.12 mg, 1.00 mmol) and the Danishefsky diene (1 mL, 5.17 mmol) in CH_2Cl_2 (4 mL) was added $BF_3·OEt_2$ (0.12 mL, 1.1 mmol) at 0°C. The reaction mixture was allowed to warm to room temperature and stirred for 24 hours at room temperature. Saturated aqueous sodium

bicarbonate (5 mL) was added and the resulting mixture extracted with CH_2Cl_2 (3 x 10 mL). The combined organic layers were dried ($MgSO_4$) and evaporated under reduced pressure to give the crude product. Purification by flash column chromatography on neutral alumina with 9:1 → 1:4 heptane-EtOAc as the eluent gave **21** (75 mg, 0.43 mmol, 43%) as a waxy brown solid.

R_f (heptane-EtOAc, 4:1) = 0.18

^1H NMR (400 MHz, $CDCl_3$); δ 6.98 (dd, *J* = 10.1, 6.1 Hz, 1H), 6.42 (dt, *J* = 2.6, 0.8 Hz, 1H), 6.29 (dd, *J* = 2.6, 1.0 Hz, 1H), 6.08 (ddd, *J* = 10.1, 1.5, 0.9 Hz, 1H), 4.02 (t, *J* = 6.1 Hz, 1H), 3.46 (ddd, *J* = 2.9, 1.9, 1.0 Hz, 1H), 3.12 (d, *J* = 2.9 Hz, 1H), 2.89 (ddd, *J* = 14.2, 7.0, 5.1 Hz, 1H), 2.52 (dd, *J* = 16.1, 5.1 Hz, 1H), 2.10 (dd, *J* = 16.1, 14.2 Hz, 1H).

$^{13}C\{^1H\}$ NMR (101 MHz, $CDCl_3$); δ 212.0, 197.6, 144.3, 142.2, 138.0, 130.8, 54.8, 47.2, 44.9, 40.5, 34.5.

FTIR (ATR) *v* (cm^{-1}): 3332 (broad), 2850, 1697 (C=O), 1675 (C=O)

HRMS unsuccessful (product unstable).

NB The relative stereochemistry given above is that expected based on consideration of the expected approach of the dienophile on the less hindered face of phototropone. It was not possible to grow suitable crystals for XRD, and NOEXY analyses in a number of solvents did not yield useful data.

***rac*-(*1R*,*2R*,*5R*,*7S*)-9-Benzyl-9-Azatricyclo[5.3.0.0^{2,5}]dec-3-en-6-one 22**

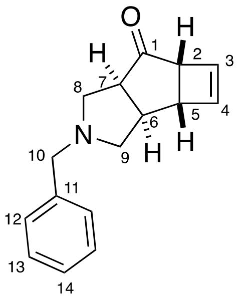

Trifluoroacetic acid (75 µL, 1.0 mmol) was added to a stirred solution of **1** (106 mg, 1.0 mmol) and *N*-methoxymethyl-*N*-(trimethylsilylmethyl)benzylamine (1.3 mL, 5.0 mmol) in CH_2Cl_2 (5 mL) at 0 °C, and the resulting solution was stirred at room temperature for 48 hours. Saturated aqueous sodium bicarbonate (5 mL) was added, and the aqueous layer was extracted with CH_2Cl_2 (3 x 10 mL). The combined organic layers were dried ($MgSO_4$) and evaporated under reduced pressure to give the crude product. Purification by flash column chromatography on silica gel with 1:9 → 3:7 EtOAc-heptane as the eluent gave **22** (172 mg, 0.72 mmol, 72%) as an orange oil.

R_f (Heptane-EtOAc, 4:1) = 0.33

^1H NMR (400 MHz, C_6D_6); δ 7.26 – 7.07 (m, 5H), 5.86 (d, *J* = 2.6 Hz, 1H), 5.73 (dd, *J* = 2.6, 1.0 Hz, 1H), 3.36 (d, *J* = 13.2 Hz, 1H), 3.34-3.32 (m, 1H), 3.23 (d, *J* = 13.2 Hz, 1H), 3.20-3.17 (m, 1H), 2.84-2.79 (m, 1H), 2.64 (d, *J* = 3.0 Hz, 1H), 2.31-2.13 (m, 4H).

$^{13}C\{^{1}H\}$ NMR (101 MHz, C_6D_6); δ 214.9, 144.2, 139.5, 135.5, 128.7, 128.6, 127.3, 60.6, 60.0, 58.6, 56.5, 51.0, 49.4, 40.0.

FTIR (ATR) *v* (cm^{-1}): 3028, 2927, 2788, 1723 (C=O).

HRMS (APCI): *m/z* calculated for $C_{16}H_{17}NO$ $[M+H]^+$ 240.1383, found 240.1374.

NB The relative stereochemistry given above is that expected based on consideration of the expected approach of the dienophile on the less hindered face of phototropone. It was not possible to grow suitable crystals for XRD (the compound is not crystalline), and NOESY analyses in a number of solvents did not yield useful data.

***rac*-(*1S,2R,4R,6R*)-3,3-Dichlorotricyclo[4.2.0.0^{2,4}]oct-7-en-5-one 23**

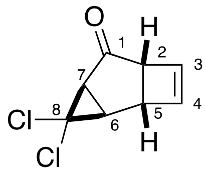

An aqueous solution of KOH (50% w/v, 5 mL) was added dropwise to a solution of **1** (106 mg, 1.00 mmol) and $^{n}Bu_4NCl$ (14 mg, 0.05 mmol) in chloroform (25 mL) at 0 °C under argon, then stirred at room temperature for 5 hours. The reaction mixture was quenched with saturated aqueous ammonium chloride solution (10 mL), the layers were separated, and the aqueous layer was extracted with EtOAc (3 x 10 mL). The combined organic layers were dried ($MgSO_4$) and evaporated under reduced pressure to yield the crude product. Purification by flash column chromatography on silica gel with 9:1 heptane-EtOAc as the eluent gave **23** (75 mg, 0.39 mmol, 39%) as an orange oil.

R_f (4:1 Heptane-EtOAc) = 0.58

^{1}H NMR (400 MHz, $CDCl_3$); δ 6.39 (dd, *J* = 2.6, 0.7 Hz, 1H), 6.08 (dd, *J* = 2.6, 1.1 Hz, 1H), 3.55 (t, *J* = 2.3 Hz, 1H), 3.29 (dd, *J* = 2.3, 1.1 Hz, 1H), 2.74 (dd, *J* = 6.0, 2.0 Hz, 1H), 2.69-2.67 (dd, *J* = 6.0, 2.1 Hz, 1H).

$^{13}C\{^{1}H\}$ NMR (101 MHz, $CDCl_3$); δ 202.3, 140.8, 136.1, 62.1, 55.4, 44.5, 44.4, 39.7.

FTIR (ATR) *v* (cm^{-1}): 3444, 3058, 2931, 1723 (C=O).

HRMS (APCI): *m/z* calculated for $C_8H_6OCl_2$ $[M+H]^+$ 188.9868, found 188.9863.

2. Crystallographic Data for 20

A suitable crystal (grown from slow evaporation from $CHCl_3$ at room temperature) was selected and mounted on a Mitegen loop, using Paratone-N oil, on a SuperNova, Dual, Cu at home/near, AtlasS2 diffractometer. The crystal was kept at 100(2) K during data collection. Using Olex2,[2] the structure was solved with the SHELXT[3] structure solution program using Intrinsic Phasing and refined with the SHELXL[4] refinement package using Least Squares minimisation.

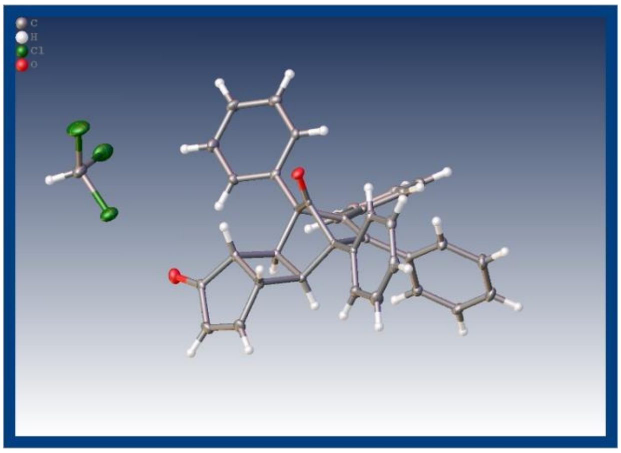

Thermal ellipsoids are drawn with 50% probability.

CCDC number	2224934
Chemical formula	$C_{37}H_{27}Cl_3O_2$
Formula weight	609.93
Temperature / K	100(2)
Crystal system	monoclinic
Space group	P21/n
a / Å	12.1171(5)
b / Å	17.1143(8)
c / Å	14.5798(6)
α / °	90
β / °	100.687(4)
γ / °	90
Volume / $Å^3$	2971.1(2)
Z	4
P calc g/cm^3	1.364
M / mm^{-1}	0.342
F(000)	1264.0
Crystal size / mm^3	0.1 × 0.07 × 0.05
Radiation	Mo Kα (λ = 0.71073)
2Θ range for data collection / °	6.52 to 59.022
Index ranges	-15 ≤ h ≤ 15, -20 ≤ k ≤ 23, -19 ≤ l ≤ 15
Reflections collected	24846
Independent reflections	7249 [R_{int} = 0.0429, R_{sigma} = 0.0421]
Data/restraints/parameters	7249/0/379
Goodness-of-fit on F^2	1.032
Final R indexes [I>=2σ (I)]	R1 = 0.0592, wR2 = 0.1454
Final R indexes [all data]	R1 = 0.0739, wR2 = 0.1547
Largest diff. peak/hole / e^- $Å^{-3}$	1.57/-1.05

3. References for the Experimental Section

1. Svensson, T. Convenient Photochemical Synthesis of 2-Bicyclo[3.2.0]heptanone, 2-Bicyclo[3.2.0]hept-6-enone, and 4-Tetracyclo[7.2.1.$0^{2,8}$.$0^{3,7}$]dodecanone. *Chem Scr*. **1973**, *3*, 171-175.
2. Dolomanov, O. V.; Bourhis, L. J.; Gildea, R. J.; Howard, J. A. K.; Puschmann, H. OLEX2: A Complete Structure Solution, Refinement and Analysis Program. *J. Appl. Crystallogr.* **2009**, *42*, 339–341.
3. Sheldrick, G. M. A Short History of SHELX. *Acta Crystallogr. Sect. A Found. Crystallogr.* **2008**, *64*, 112–122.
4. Sheldrick, G. M. SHELXT - Integrated Space-Group and Crystal-Structure Determination. *Acta Crystallogr. Sect. A Found. Crystallogr.* **2015**, *71*, 3–8.

^{1}H NMR (400 MHz, $CDCl_3$)

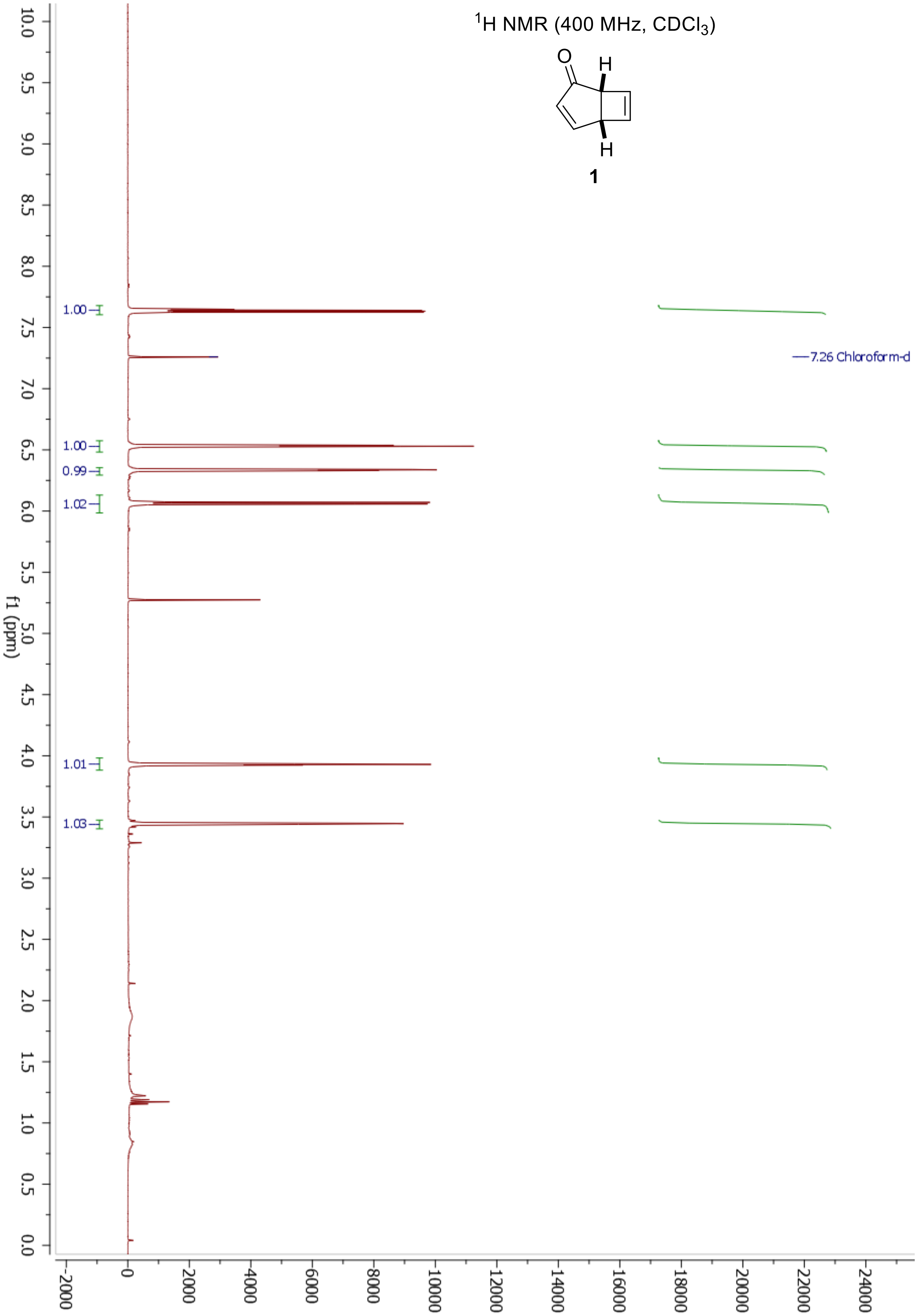

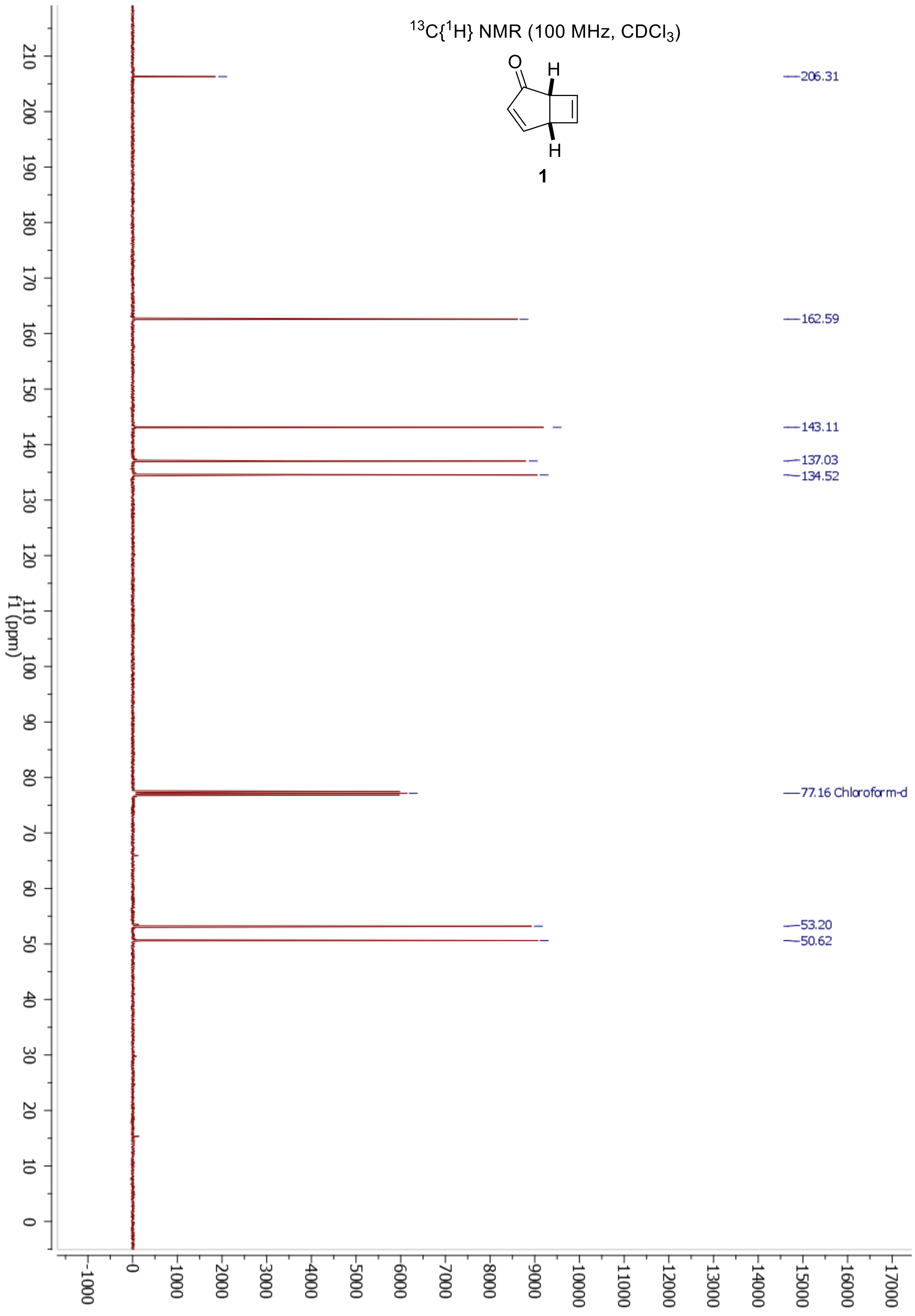
$^{13}C\{^{1}H\}$ NMR (100 MHz, $CDCl_3$)
O
H
H
1
206.31
162.59
143.11
137.03
134.52
77.16 Chloroform-d
53.20
50.62
f1 (ppm)

^1H NMR (400 MHz, $CDCl_3$)

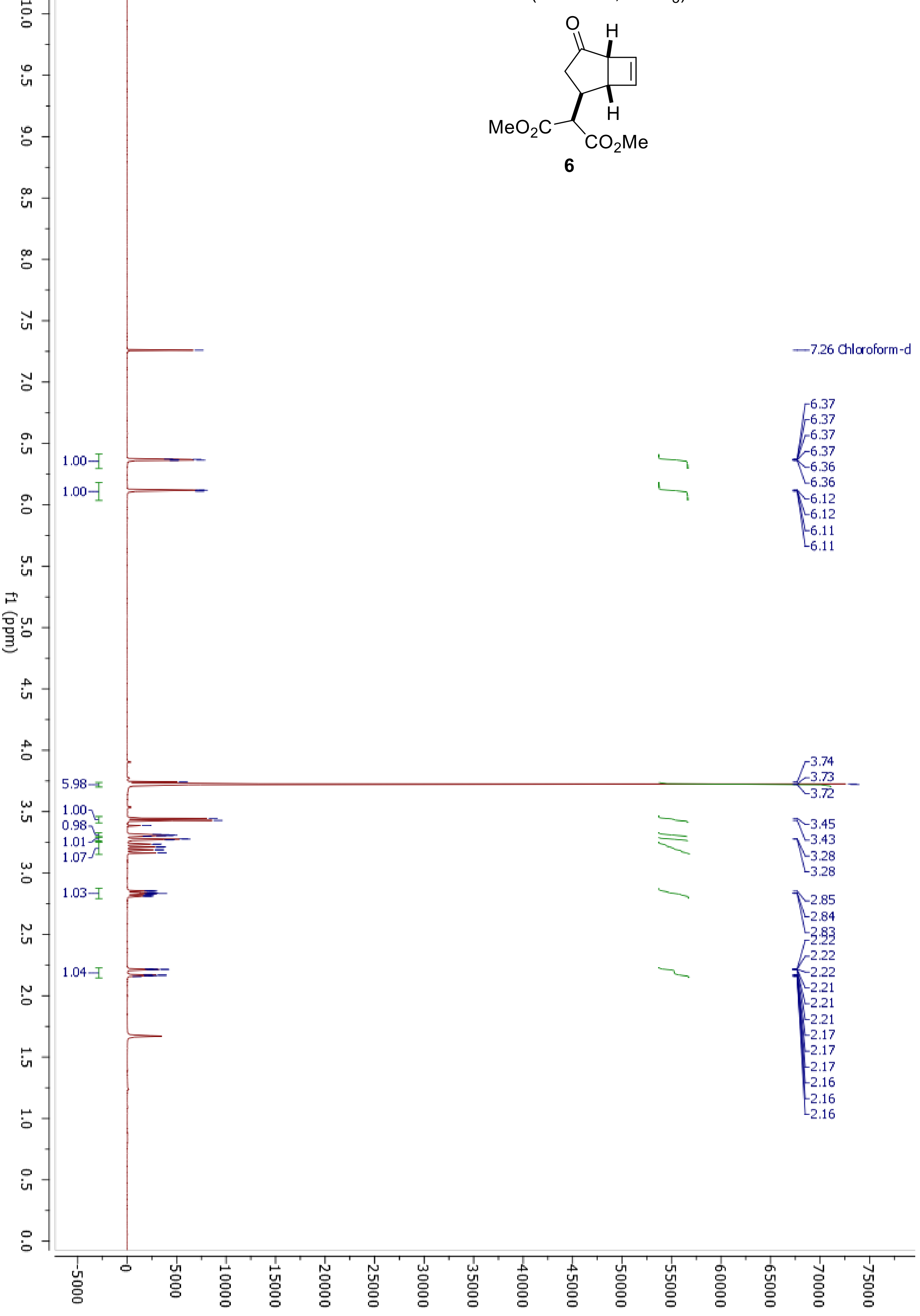

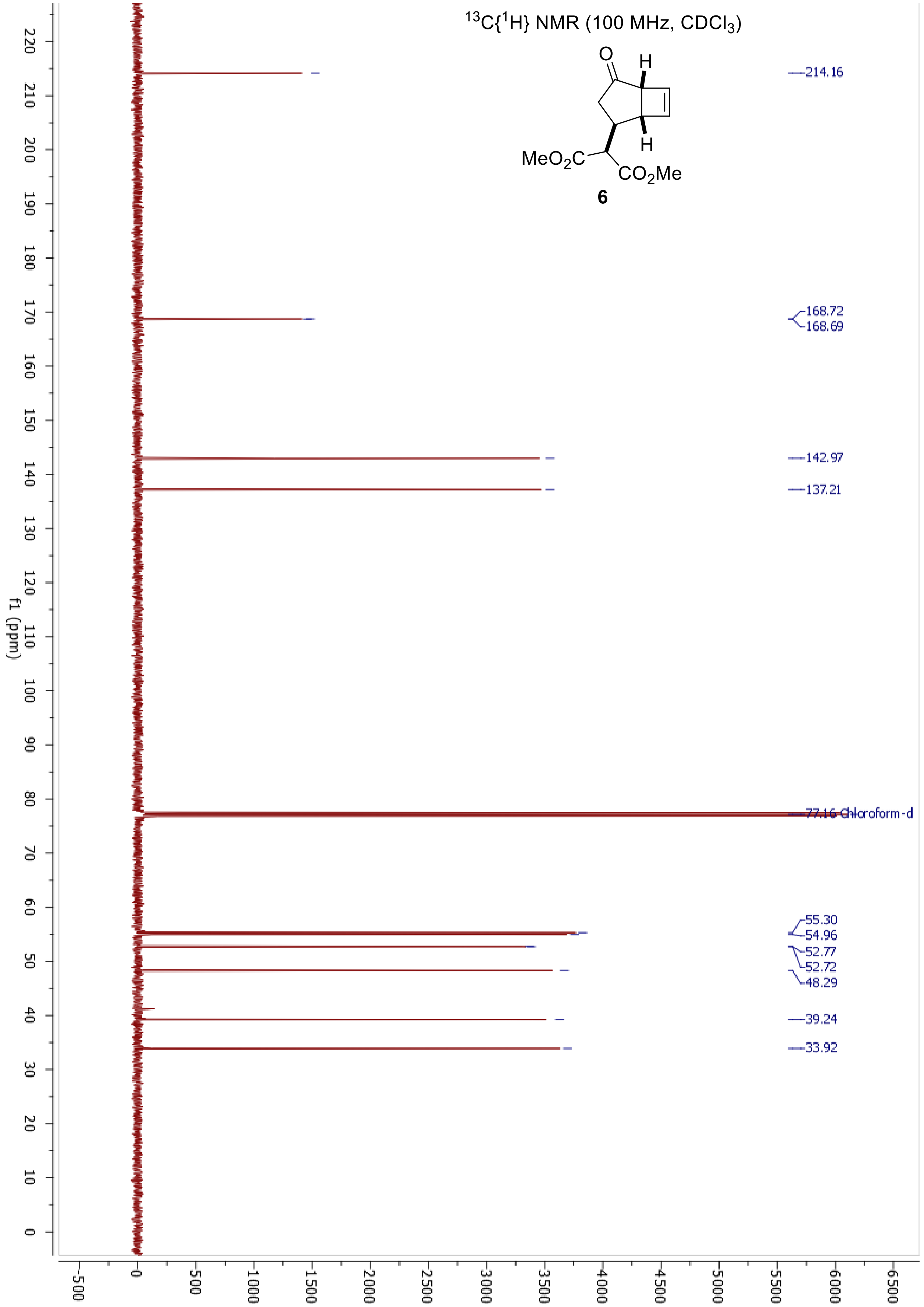
$^{13}C\{^1H\}$ NMR (100 MHz, $CDCl_3$)
MeO2C
CO2Me
6
214.16
168.72
168.69
142.97
137.21
77.16 Chloroform-d
55.30
54.96
52.77
52.72
48.29
39.24
33.92
f1 (ppm)

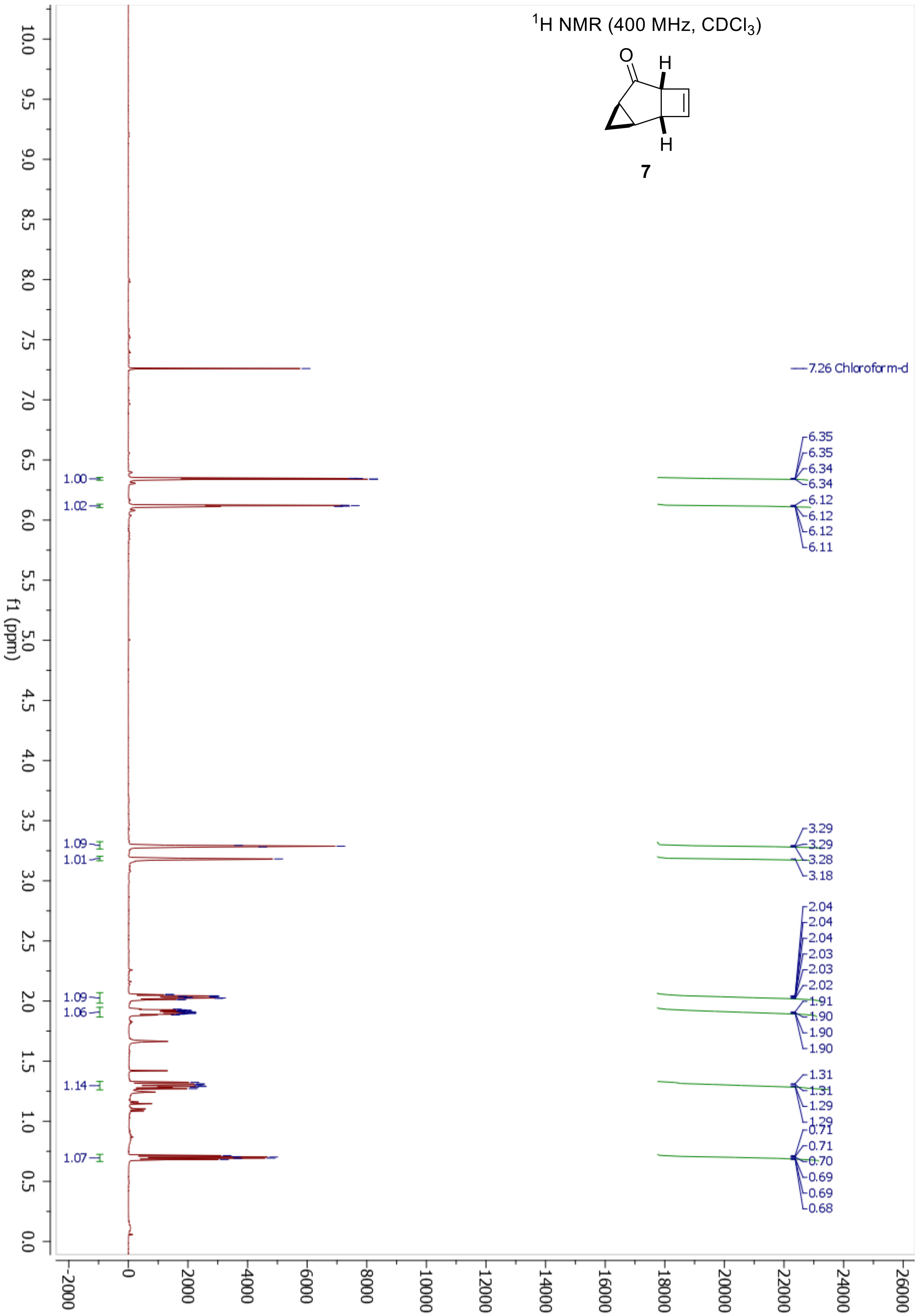

1H NMR (400 MHz, CDCl3)
7
7.26 Chloroform-d
6.35
6.35
6.34
6.34
6.12
6.12
6.12
6.11
3.29
3.29
3.28
3.18
2.04
2.04
2.04
2.03
2.03
2.02
1.91
1.90
1.90
1.90
1.31
1.31
1.29
1.29
0.71
0.71
0.70
0.69
0.69
0.68
1.00
1.02
1.09
1.01
1.09
1.06
1.14
1.07
f1 (ppm)

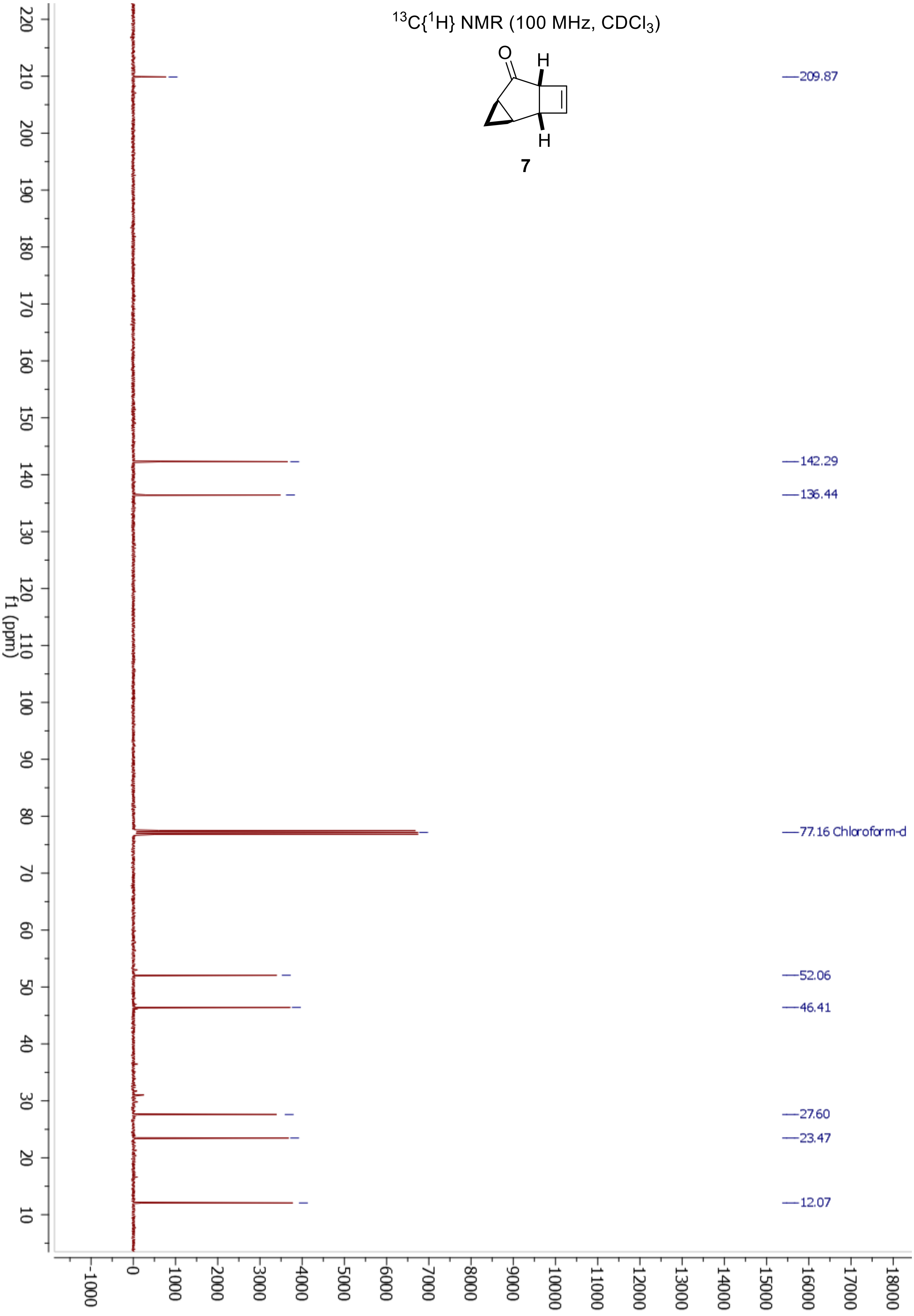

$^{13}C\{^{1}H\}$ NMR (100 MHz, $CDCl_3$)
O
H
H
7
209.87
142.29
136.44
77.16 Chloroform-d
52.06
46.41
27.60
23.47
12.07
f1 (ppm)

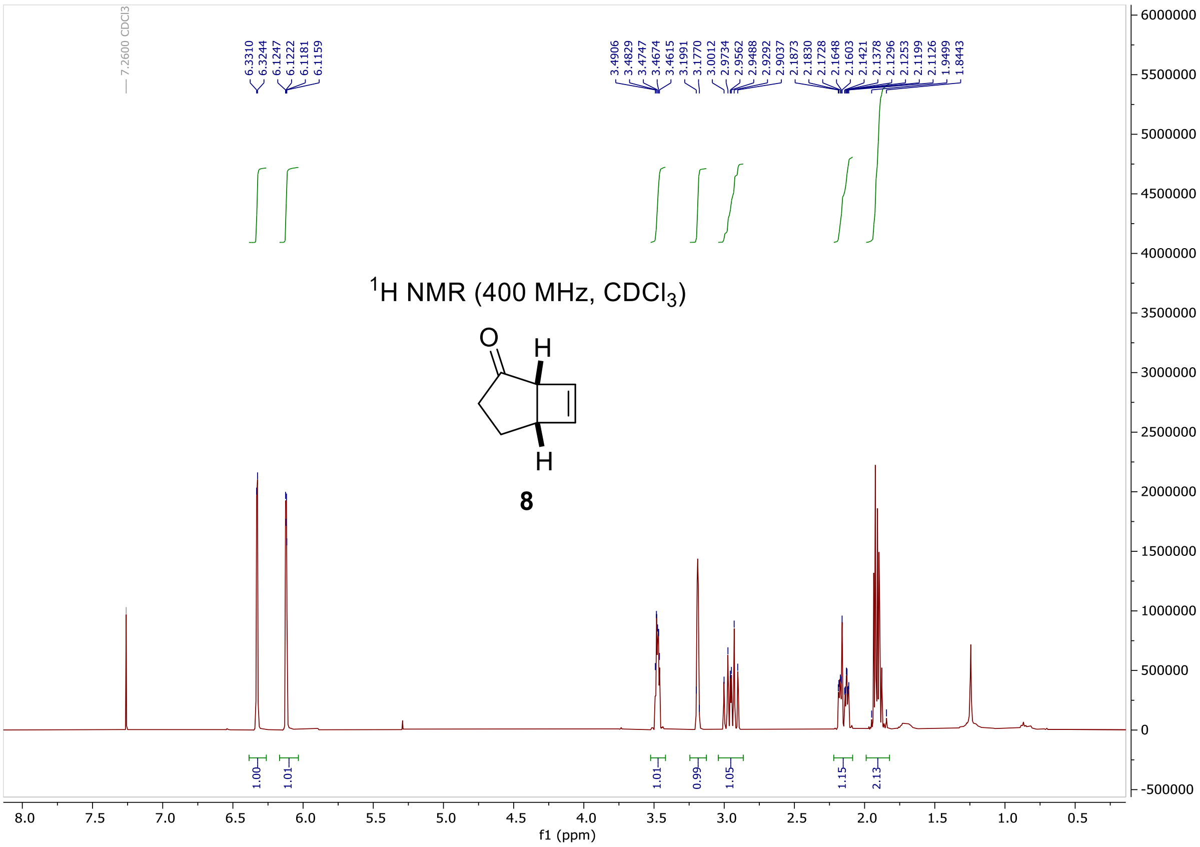
^{1}H NMR (400 MHz, $CDCl_3$)
8
7.2600 CDCl3
6.3310
6.3244
6.1247
6.1222
6.1181
6.1159
3.4906
3.4829
3.4747
3.4674
3.4615
3.1991
3.1770
3.0012
2.9734
2.9562
2.9488
2.9292
2.9037
2.1873
2.1830
2.1728
2.1648
2.1603
2.1421
2.1378
2.1296
2.1253
2.1199
2.1126
1.9499
1.8443
1.00
1.01
1.01
0.99
1.05
1.15
2.13
f1 (ppm)

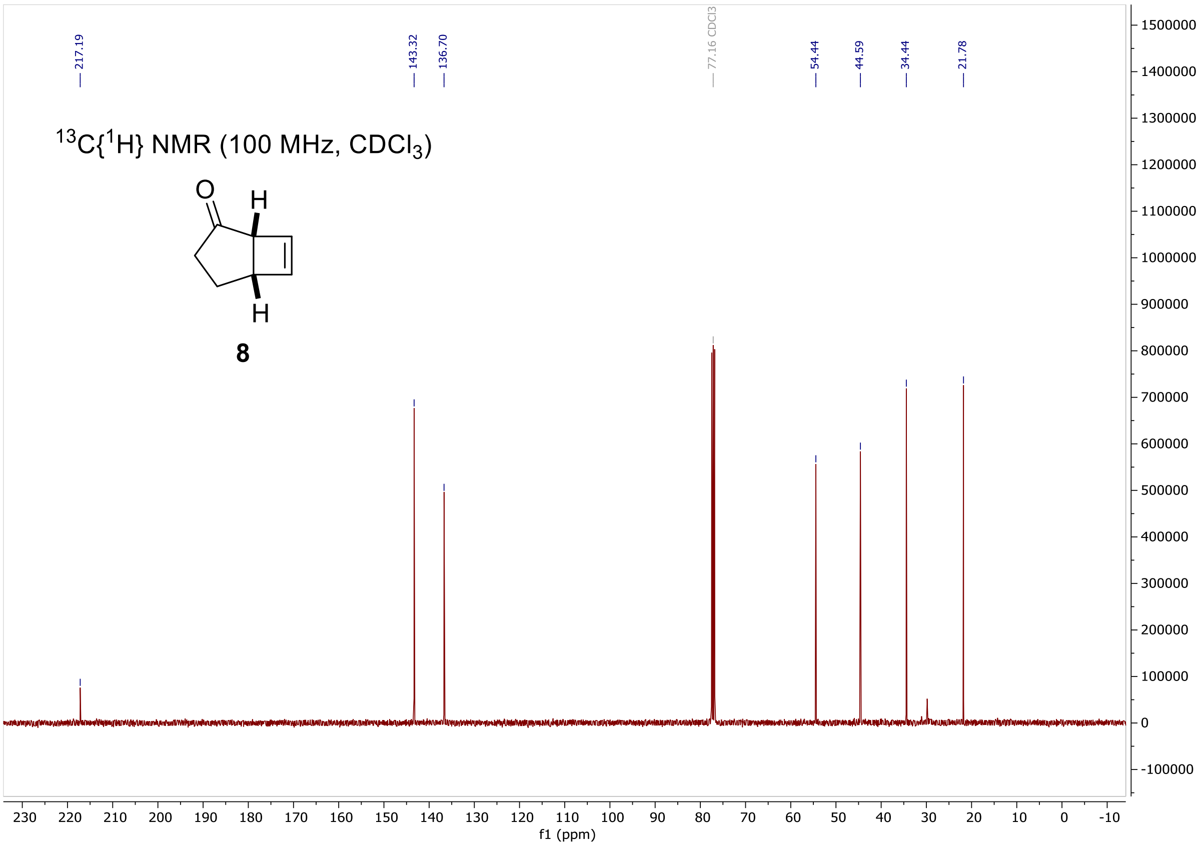
^{13}C{^{1}H} NMR (100 MHz, $CDCl_3$)
8
217.19
143.32
136.70
77.16 CDCl3
54.44
44.59
34.44
21.78
f1 (ppm)

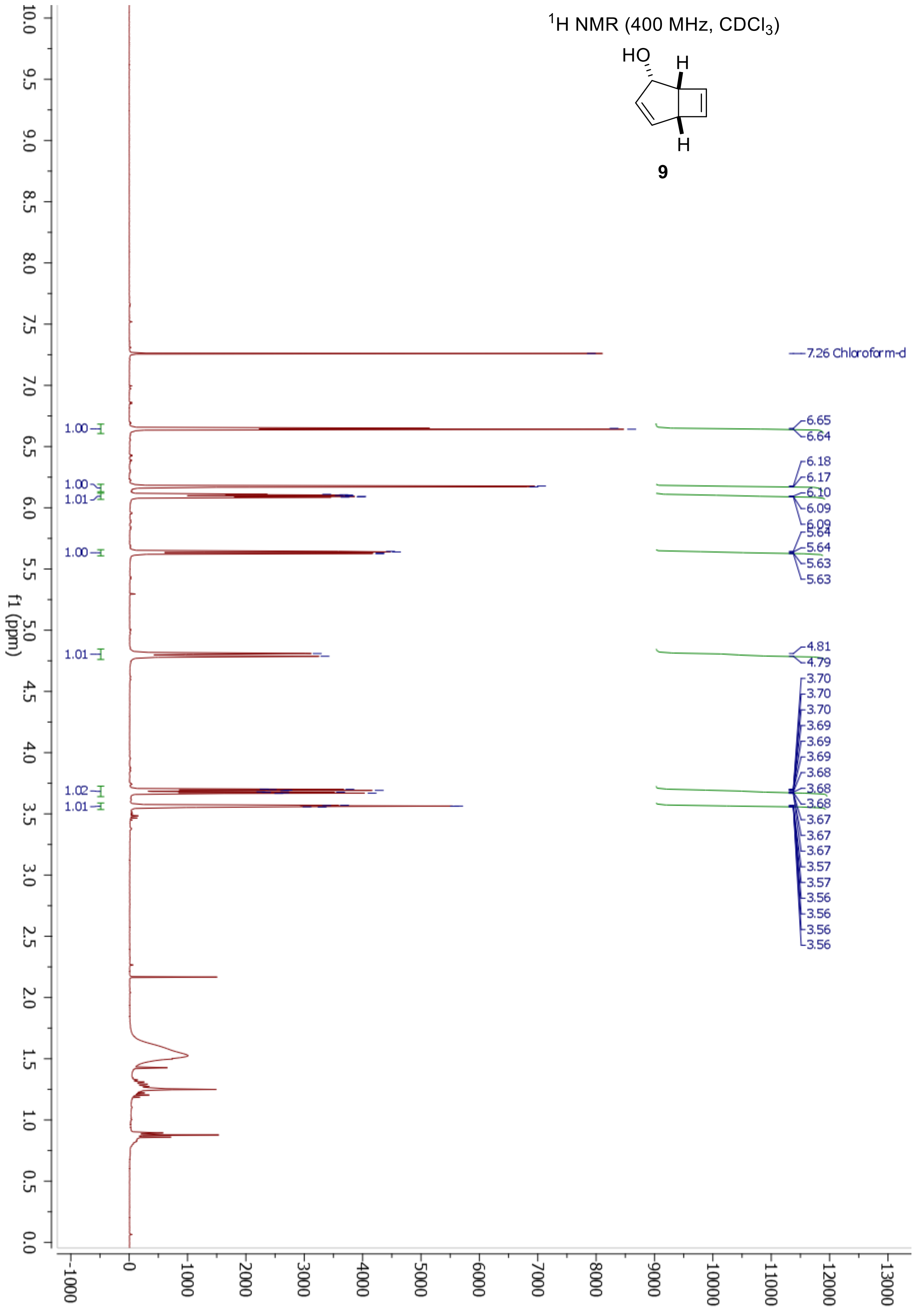

^{1}H NMR (400 MHz, CDCl$_3$)
HO
H
H
9
7.26 Chloroform-d
6.65
6.64
6.18
6.17
6.10
6.09
6.09
5.64
5.64
5.63
5.63
4.81
4.79
3.70
3.70
3.70
3.69
3.69
3.69
3.68
3.68
3.68
3.67
3.67
3.67
3.57
3.57
3.56
3.56
3.56
3.56
1.00
1.00
1.01
1.00
1.01
1.02
1.01
f1 (ppm)

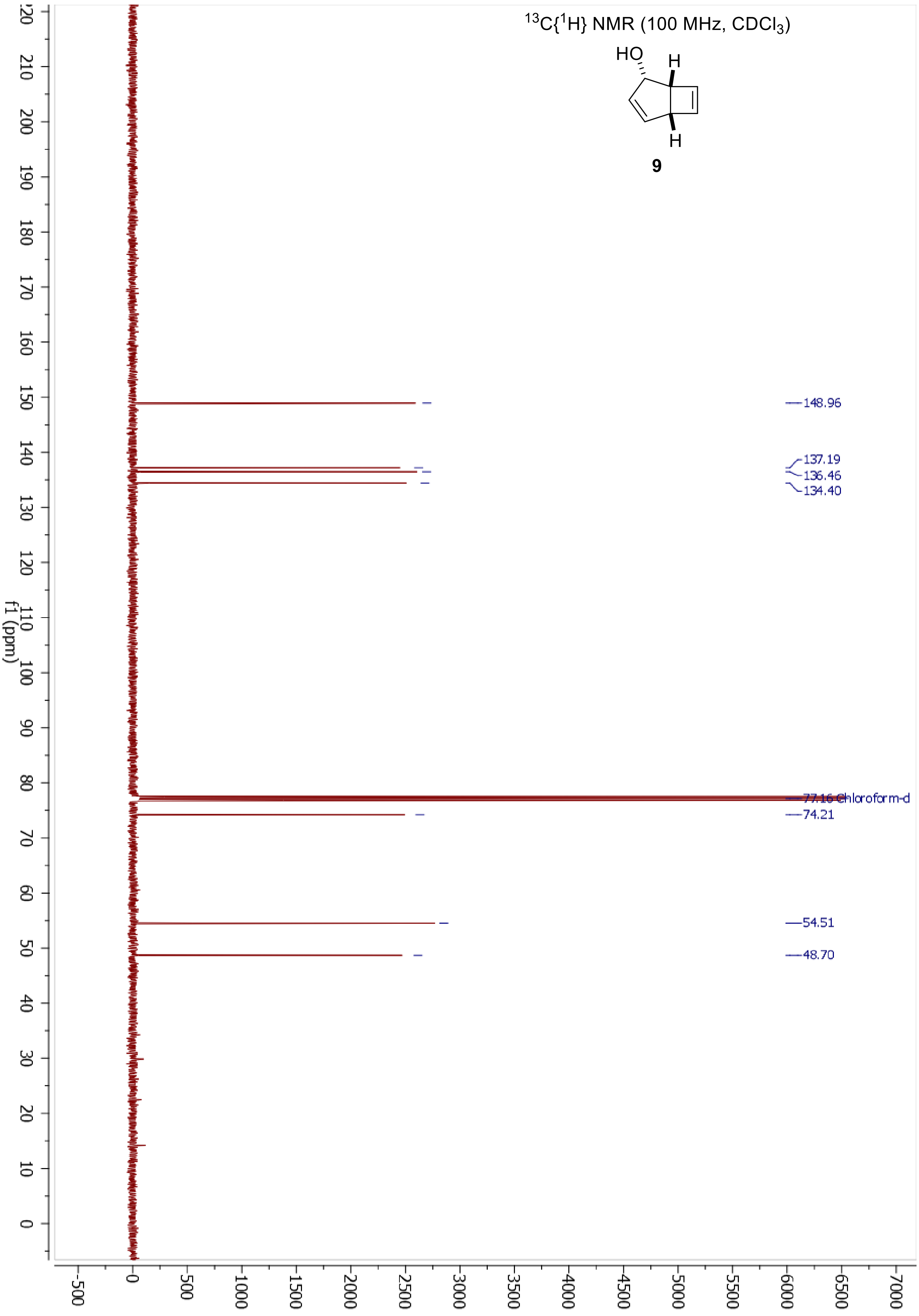

13C{1H} NMR (100 MHz, CDCl3)
HO
H
H
9
148.96
137.19
136.46
134.40
77.16 Chloroform-d
74.21
54.51
48.70
f1 (ppm)

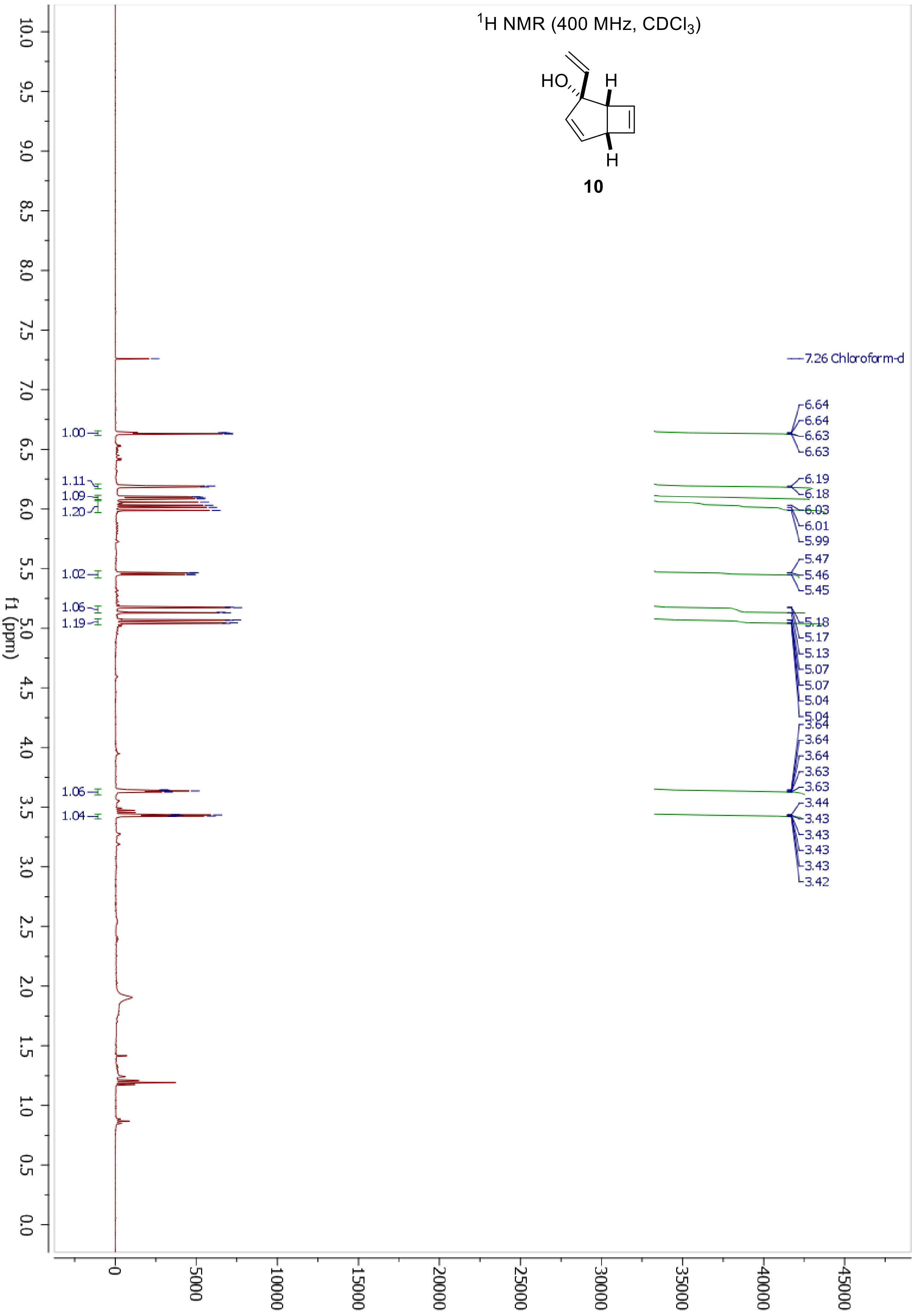

^{1}H NMR (400 MHz, CDCl$_3$)
HO
H
H
10
7.26 Chloroform-d
f1 (ppm)

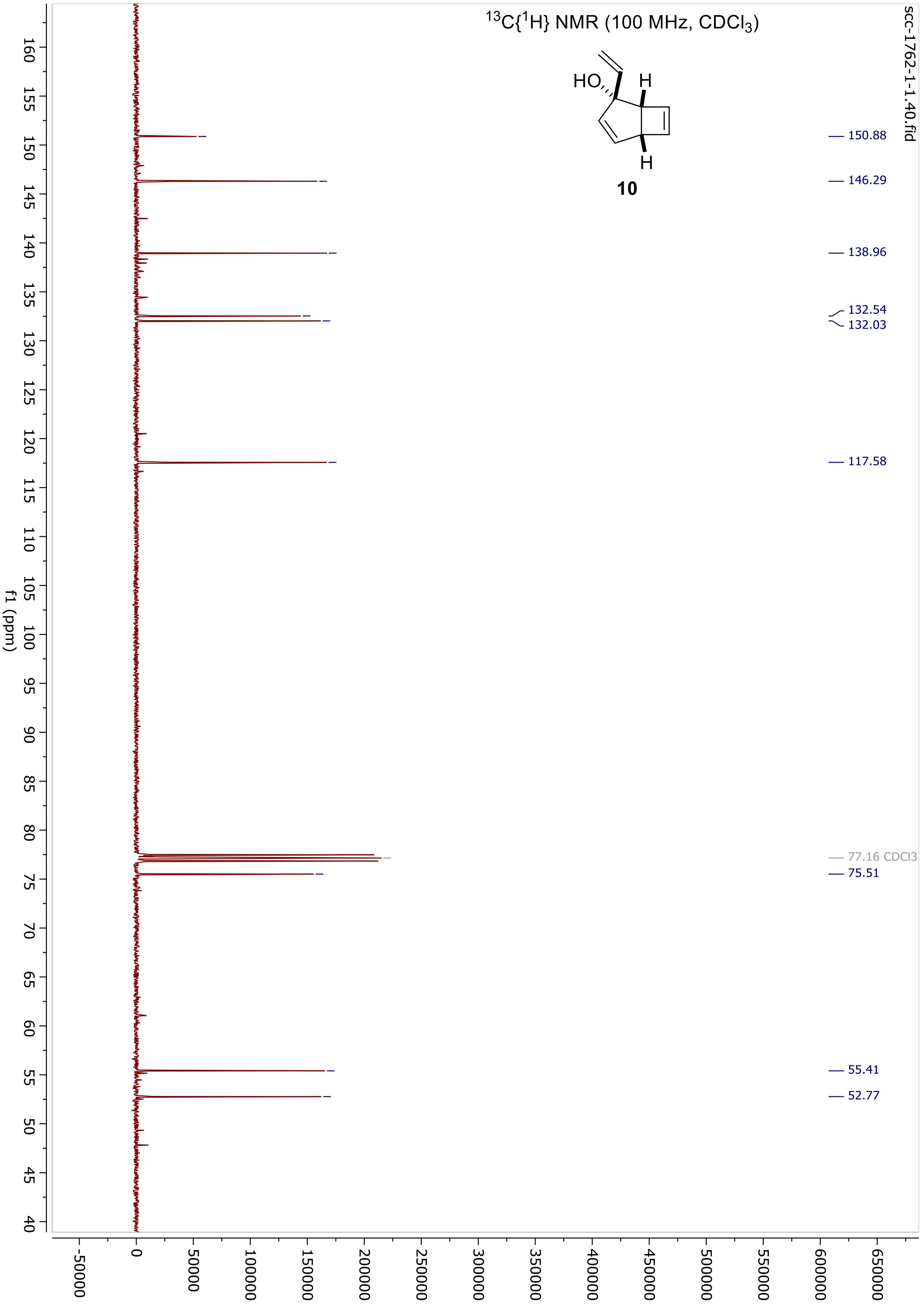

$^{13}C\{^1H\}$ NMR (100 MHz, $CDCl_3$)
HO
H
H
10
scc-1762-1-1.40.fid
150.88
146.29
138.96
132.54
132.03
117.58
77.16 CDCl3
75.51
55.41
52.77
f1 (ppm)

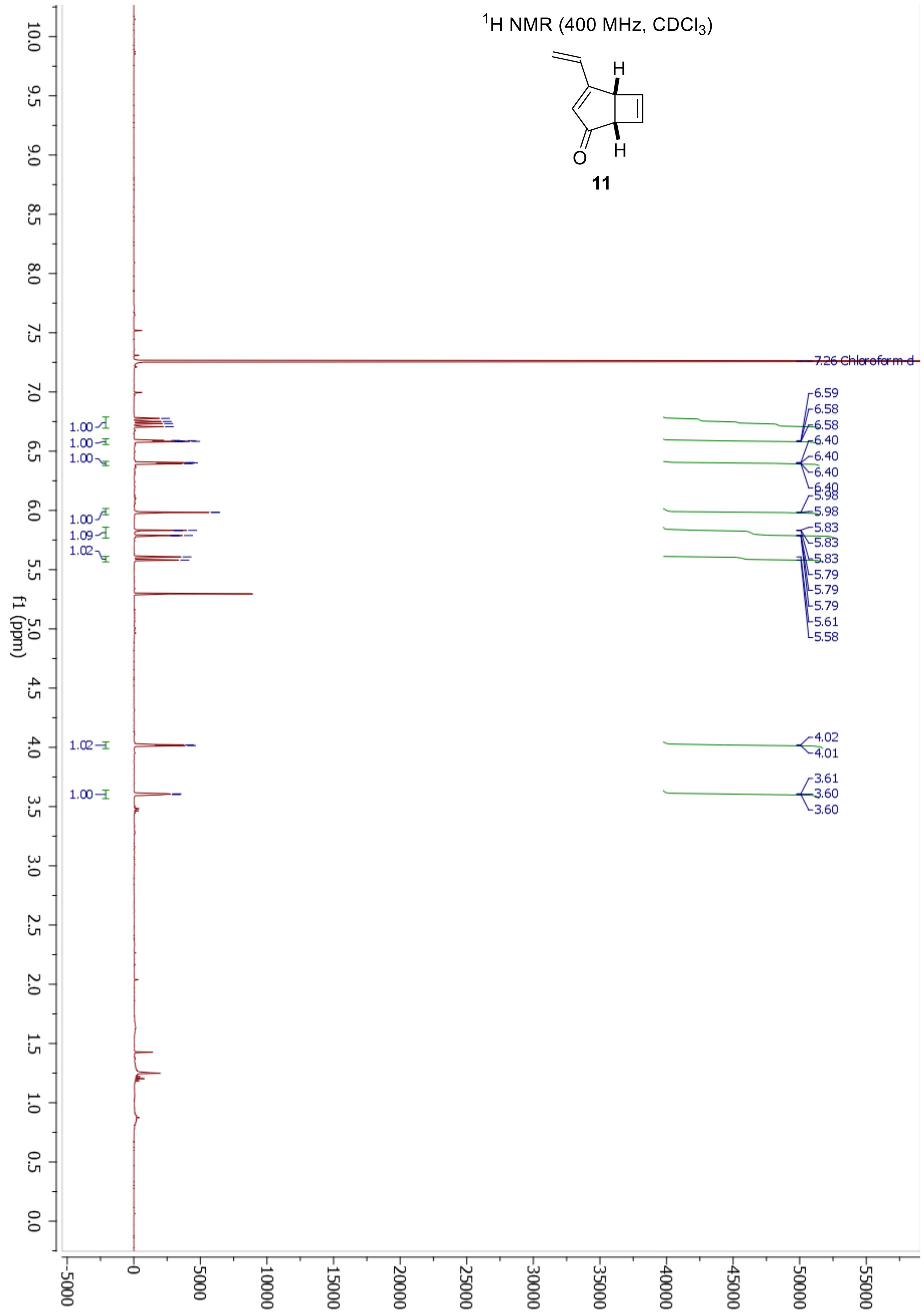

^{1}H NMR (400 MHz, CDCl$_3$)
H
H
O
11
7.26 Chloroform-d
6.59
6.58
6.58
6.40
6.40
6.40
6.40
5.98
5.98
5.83
5.83
5.83
5.79
5.79
5.79
5.61
5.58
4.02
4.01
3.61
3.60
3.60
1.00
1.00
1.00
1.00
1.09
1.02
1.02
1.00
f1 (ppm)

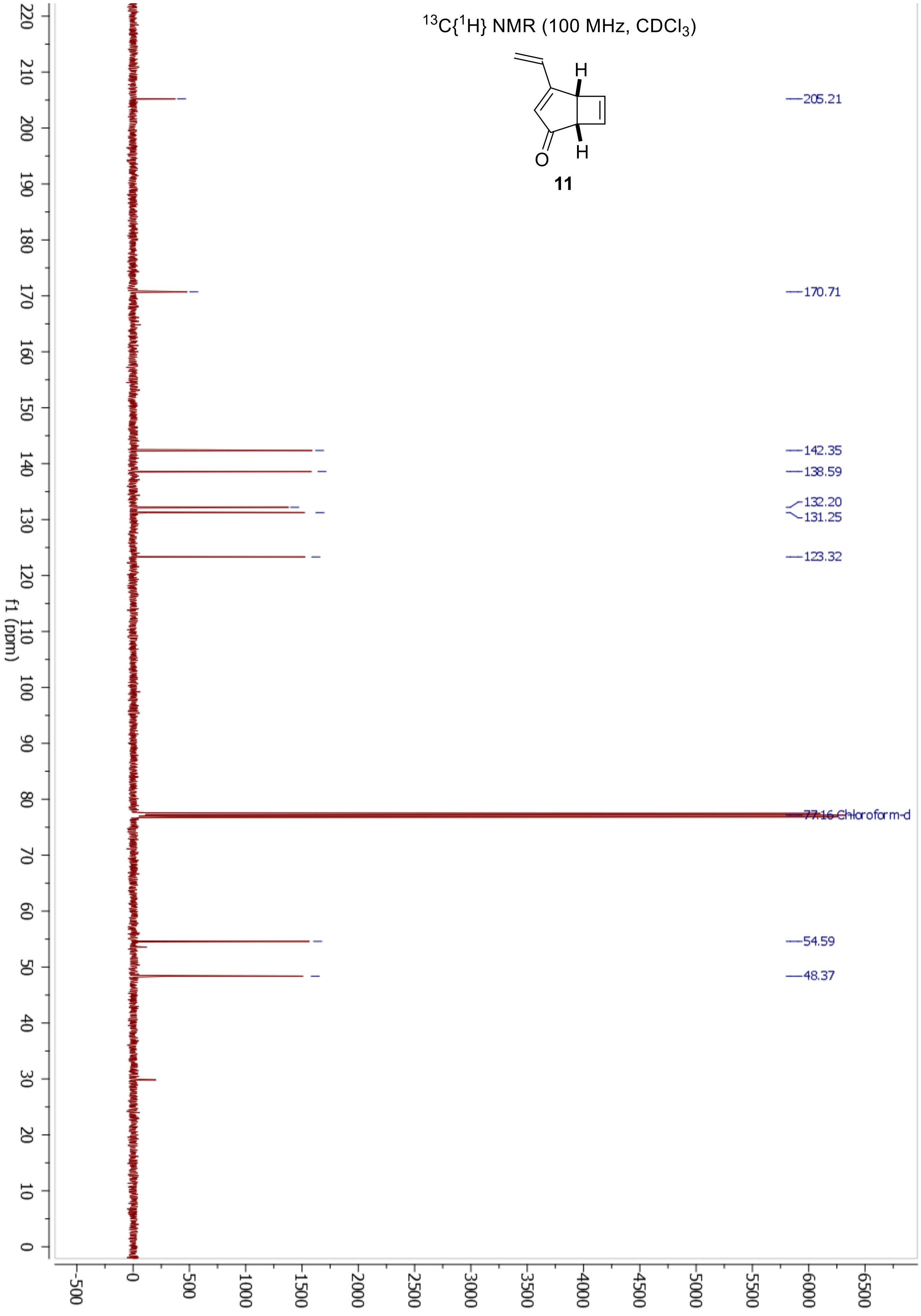

13C{1H} NMR (100 MHz, CDCl3)
H
H
O
11
205.21
170.71
142.35
138.59
132.20
131.25
123.32
77.16 Chloroform-d
54.59
48.37
f1 (ppm)

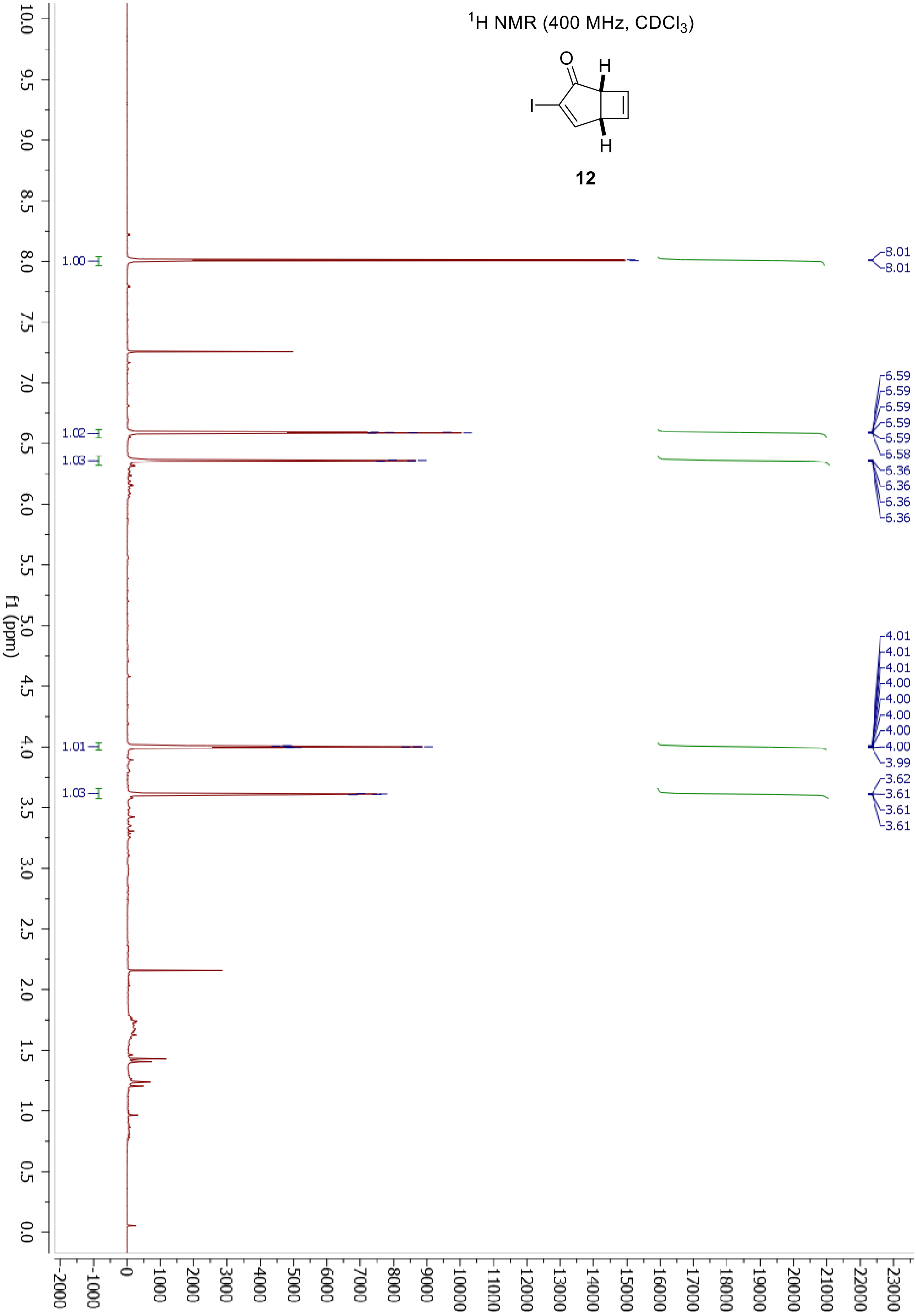

1H NMR (400 MHz, CDCl3)
O
H
I
H
12
8.01
8.01
6.59
6.59
6.59
6.59
6.59
6.58
6.36
6.36
6.36
6.36
4.01
4.01
4.01
4.00
4.00
4.00
4.00
4.00
3.99
3.62
3.61
3.61
3.61
1.00
1.02
1.03
1.01
1.03
f1 (ppm)

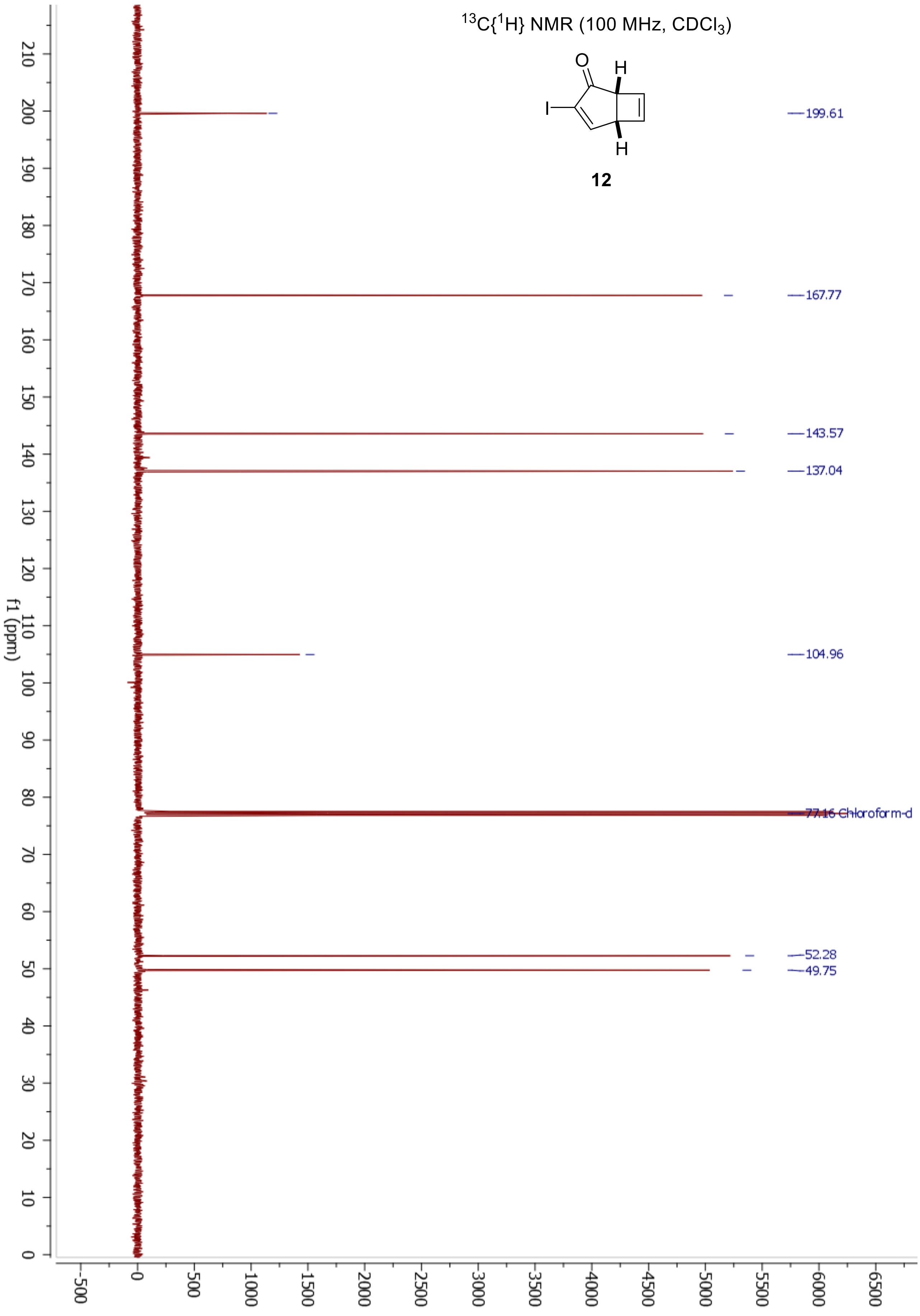
$^{13}C\{^1H\}$ NMR (100 MHz, $CDCl_3$)
O
H
I
H
12
199.61
167.77
143.57
137.04
104.96
77.16 Chloroform-d
52.28
49.75
f1 (ppm)

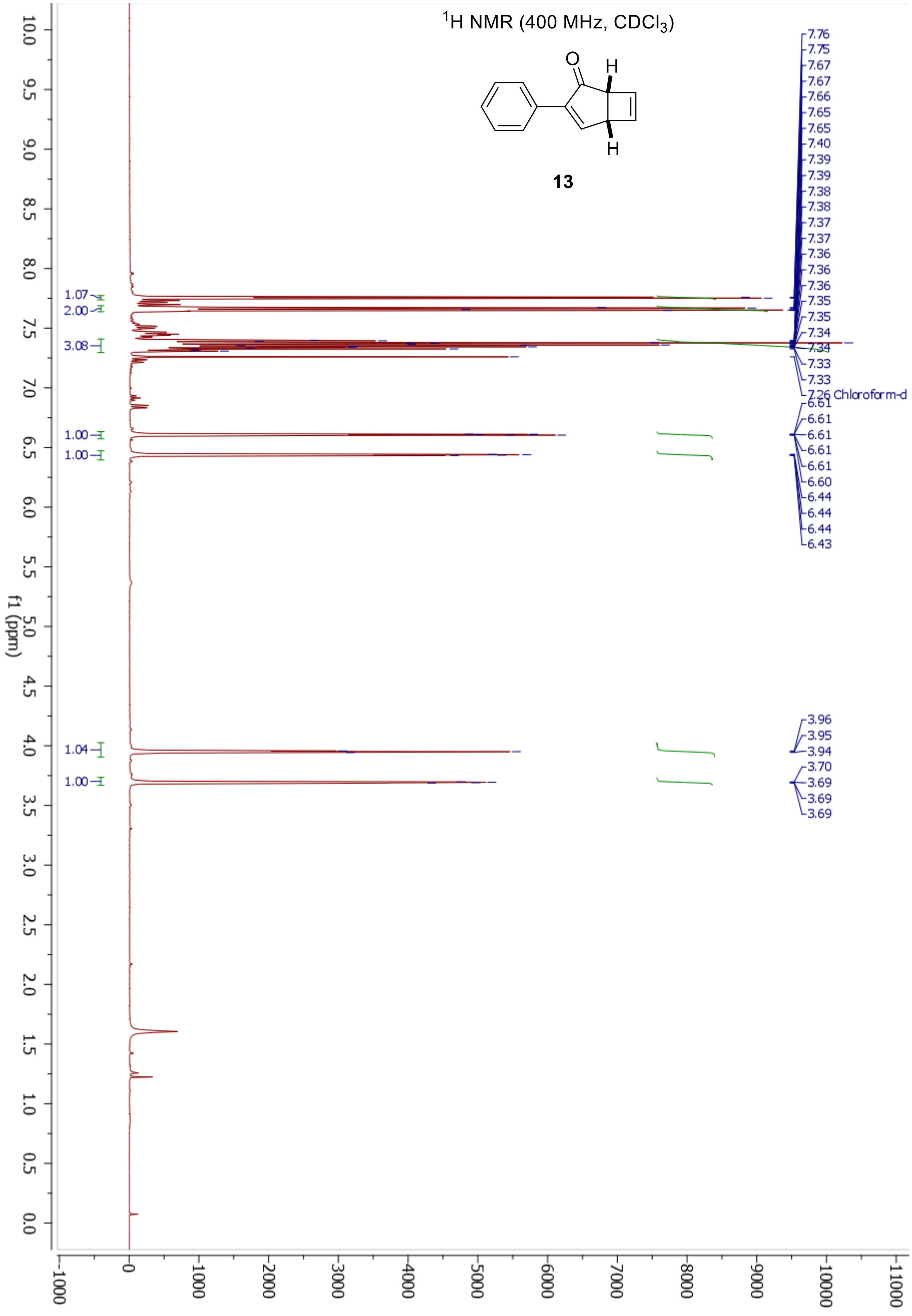

^{1}H NMR (400 MHz, CDCl$_3$)
13
7.76
7.75
7.67
7.67
7.66
7.65
7.65
7.40
7.39
7.39
7.38
7.38
7.37
7.37
7.36
7.36
7.36
7.35
7.35
7.34
7.34
7.33
7.33
7.26 Chloroform-d
6.61
6.61
6.61
6.61
6.61
6.60
6.44
6.44
6.44
6.43
3.96
3.95
3.94
3.70
3.69
3.69
3.69

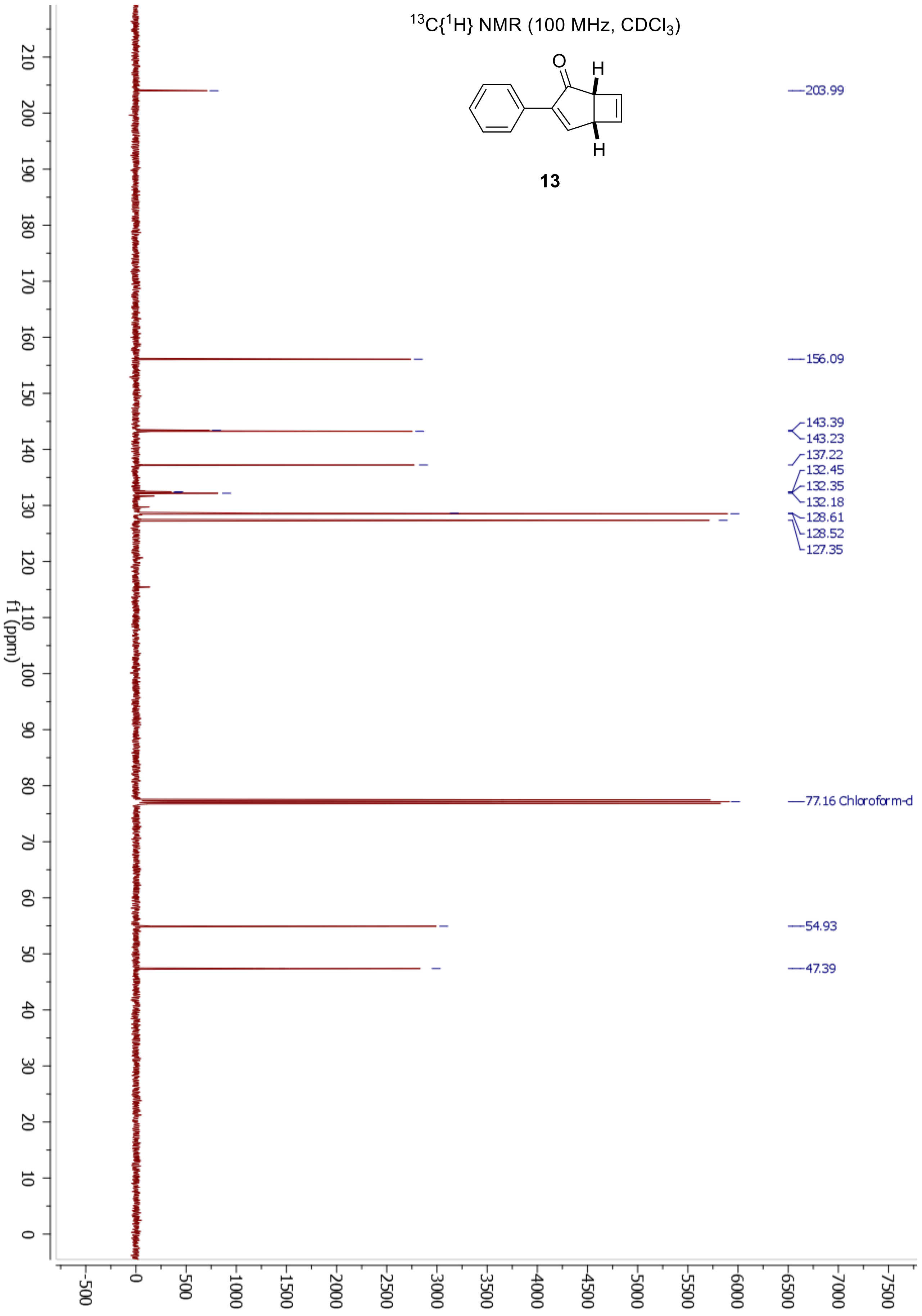
13C{1H} NMR (100 MHz, CDCl3)
O
H
H
13
203.99
156.09
143.39
143.23
137.22
132.45
132.35
132.18
128.61
128.52
127.35
77.16 Chloroform-d
54.93
47.39
f1 (ppm)

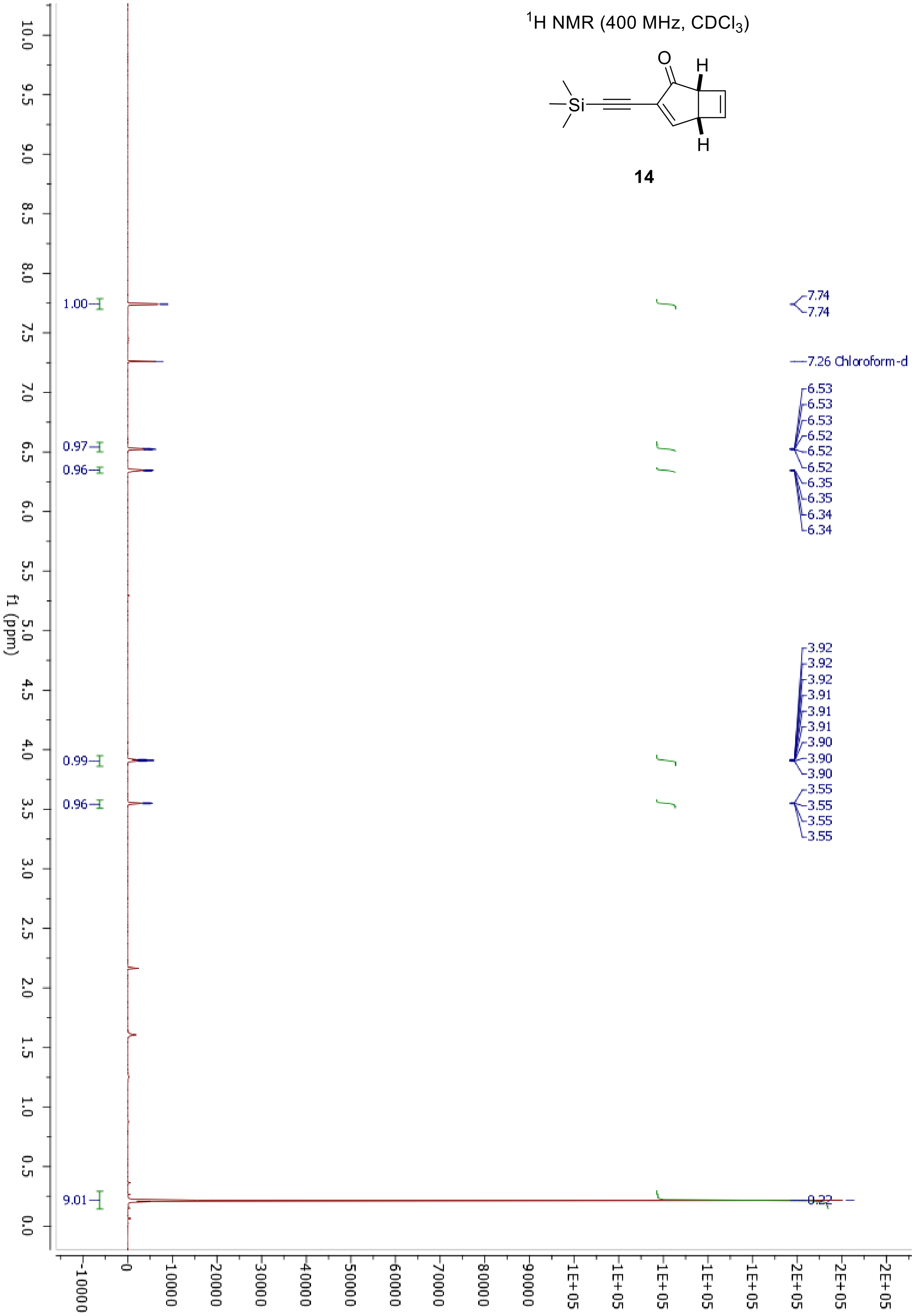

1H NMR (400 MHz, CDCl3)
O
H
Si
H
14
7.74
7.74
7.26 Chloroform-d
6.53
6.53
6.53
6.52
6.52
6.52
6.35
6.35
6.34
6.34
3.92
3.92
3.92
3.91
3.91
3.91
3.90
3.90
3.90
3.55
3.55
3.55
3.55
0.22
1.00
0.97
0.96
0.99
0.96
9.01
f1 (ppm)

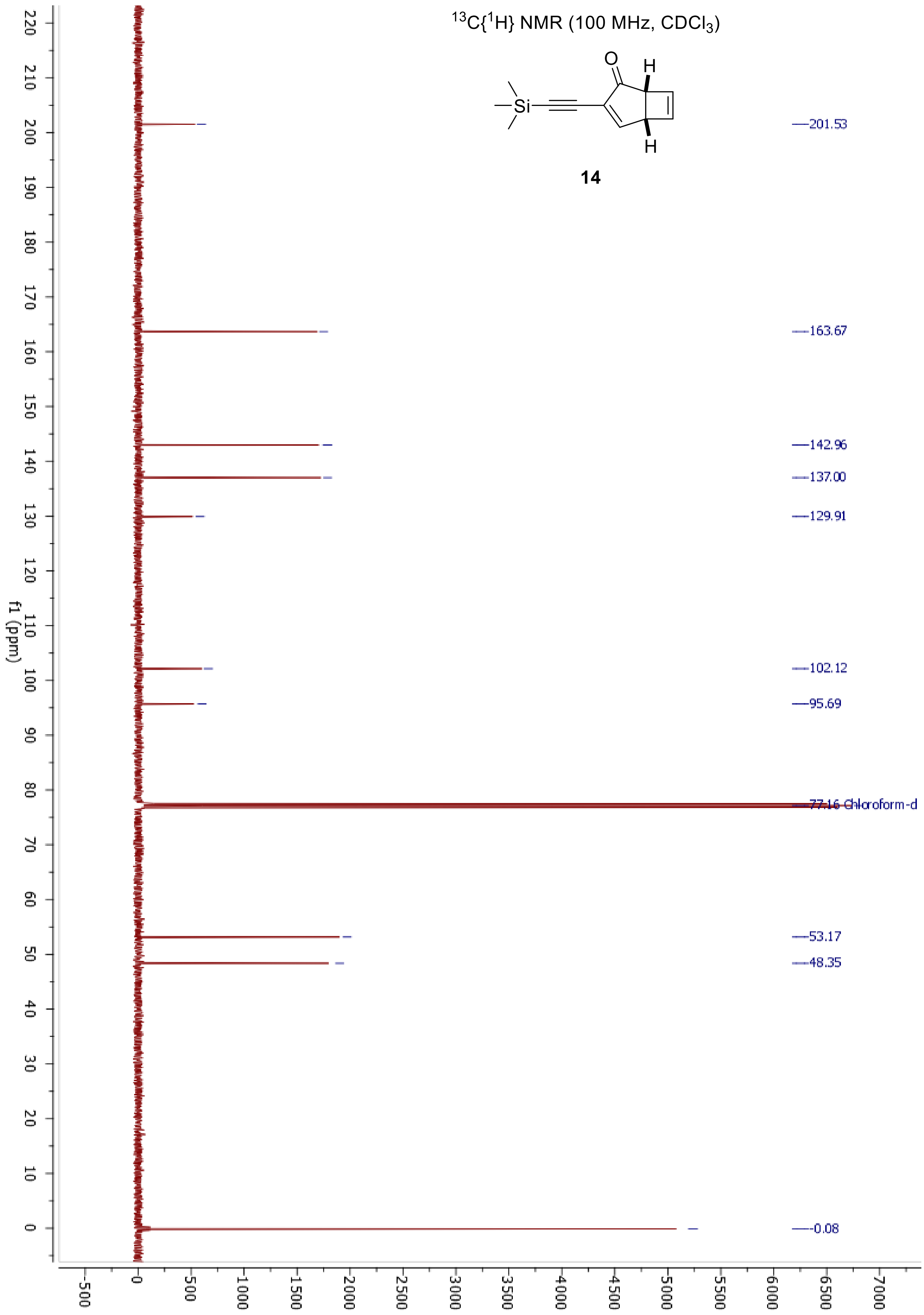
13C{1H} NMR (100 MHz, CDCl3)
O
H
Si
H
14
201.53
163.67
142.96
137.00
129.91
102.12
95.69
77.16 Chloroform-d
53.17
48.35
-0.08
f1 (ppm)

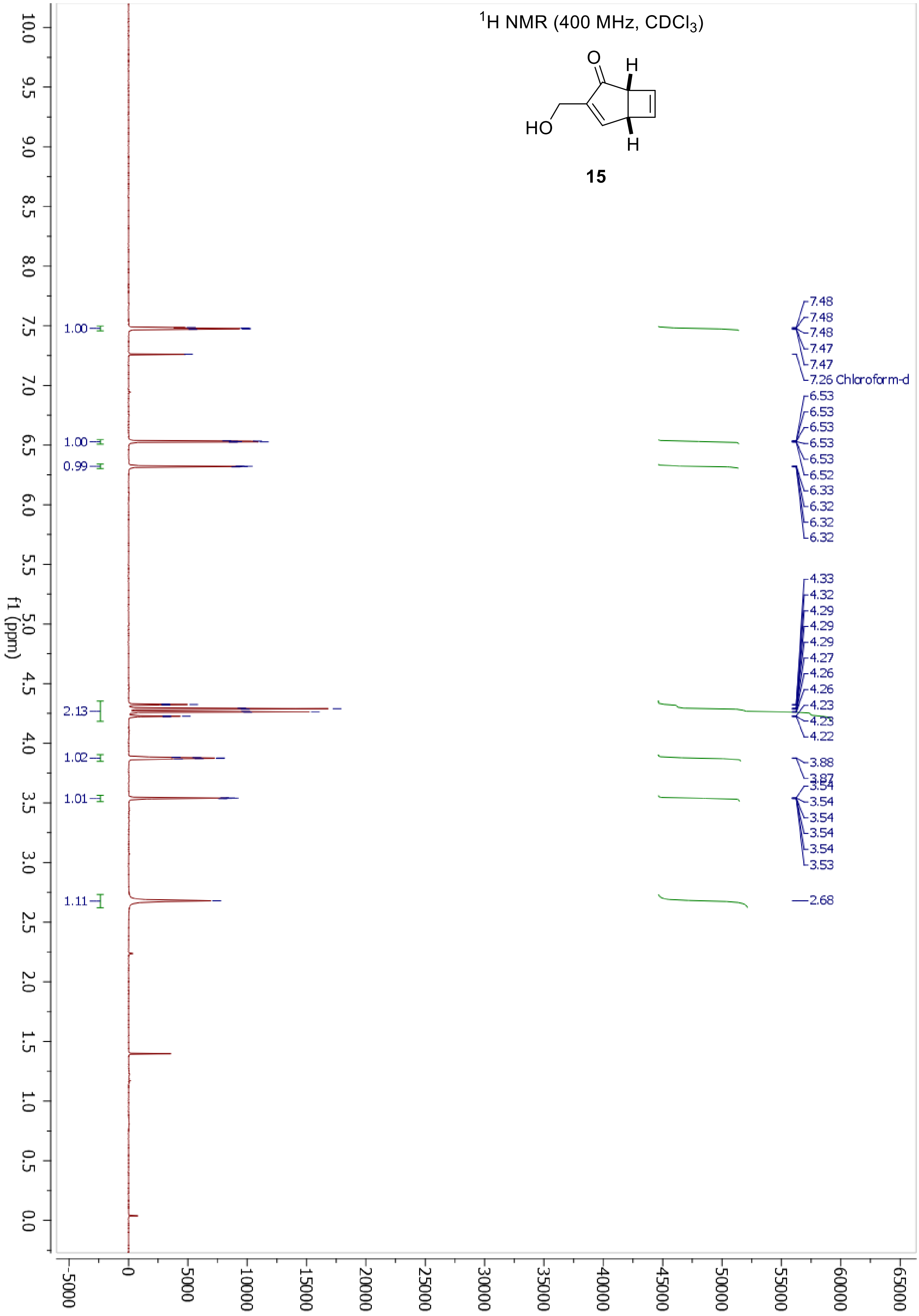

1H NMR (400 MHz, CDCl3)
15
f1 (ppm)
7.48
7.48
7.48
7.47
7.47
7.26 Chloroform-d
6.53
6.53
6.53
6.53
6.53
6.52
6.33
6.32
6.32
6.32
4.33
4.32
4.29
4.29
4.29
4.27
4.26
4.26
4.23
4.23
4.22
3.88
3.87
3.54
3.54
3.54
3.54
3.54
3.53
2.68
1.00
1.00
0.99
2.13
1.02
1.01
1.11

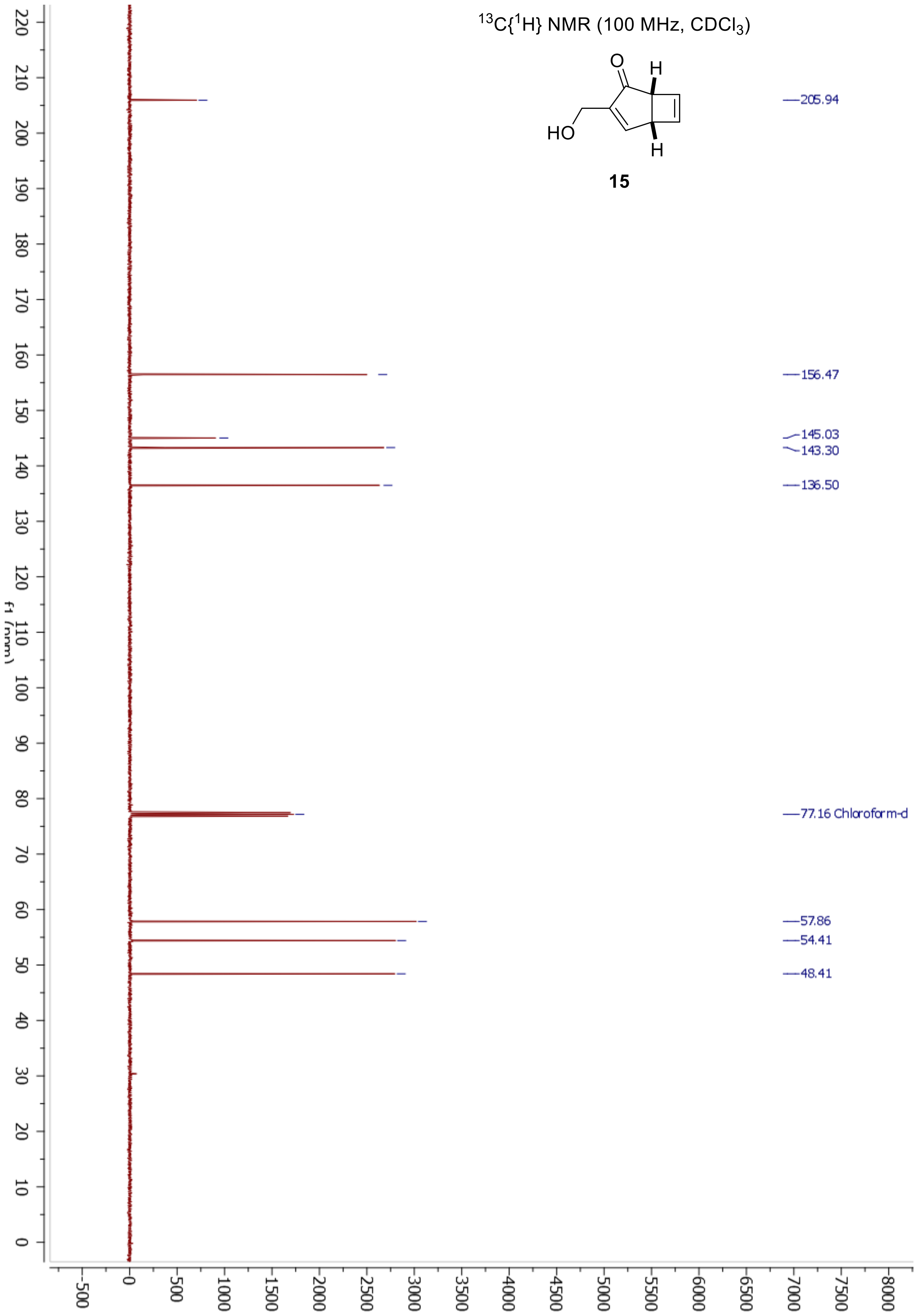

13C{1H} NMR (100 MHz, CDCl3)
O
H
HO
H
15
205.94
156.47
145.03
143.30
136.50
77.16 Chloroform-d
57.86
54.41
48.41
f1 (ppm)

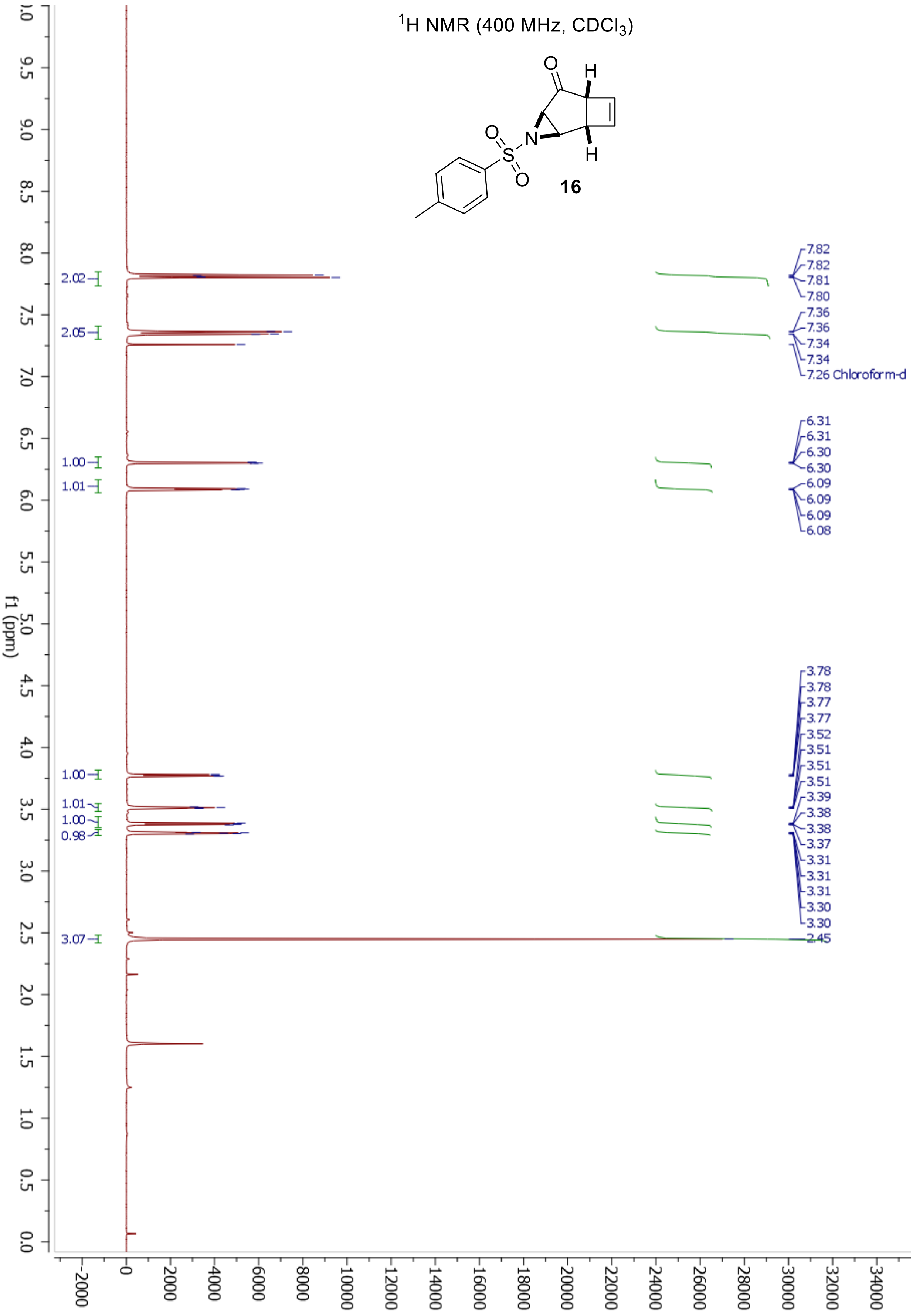
1H NMR (400 MHz, CDCl3)
16

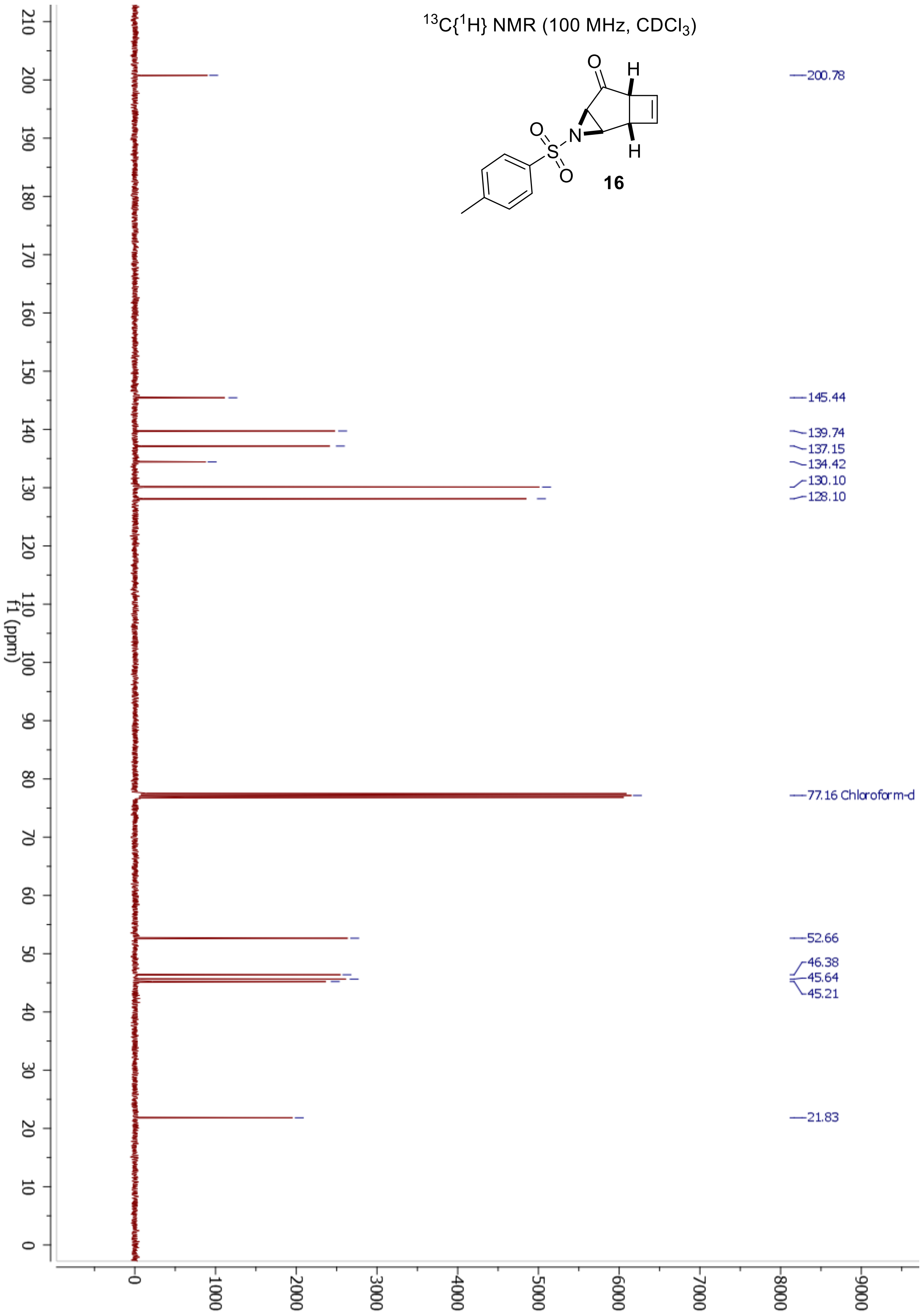
13C{1H} NMR (100 MHz, CDCl3)
16
200.78
145.44
139.74
137.15
134.42
130.10
128.10
77.16 Chloroform-d
52.66
46.38
45.64
45.21
21.83
f1 (ppm)

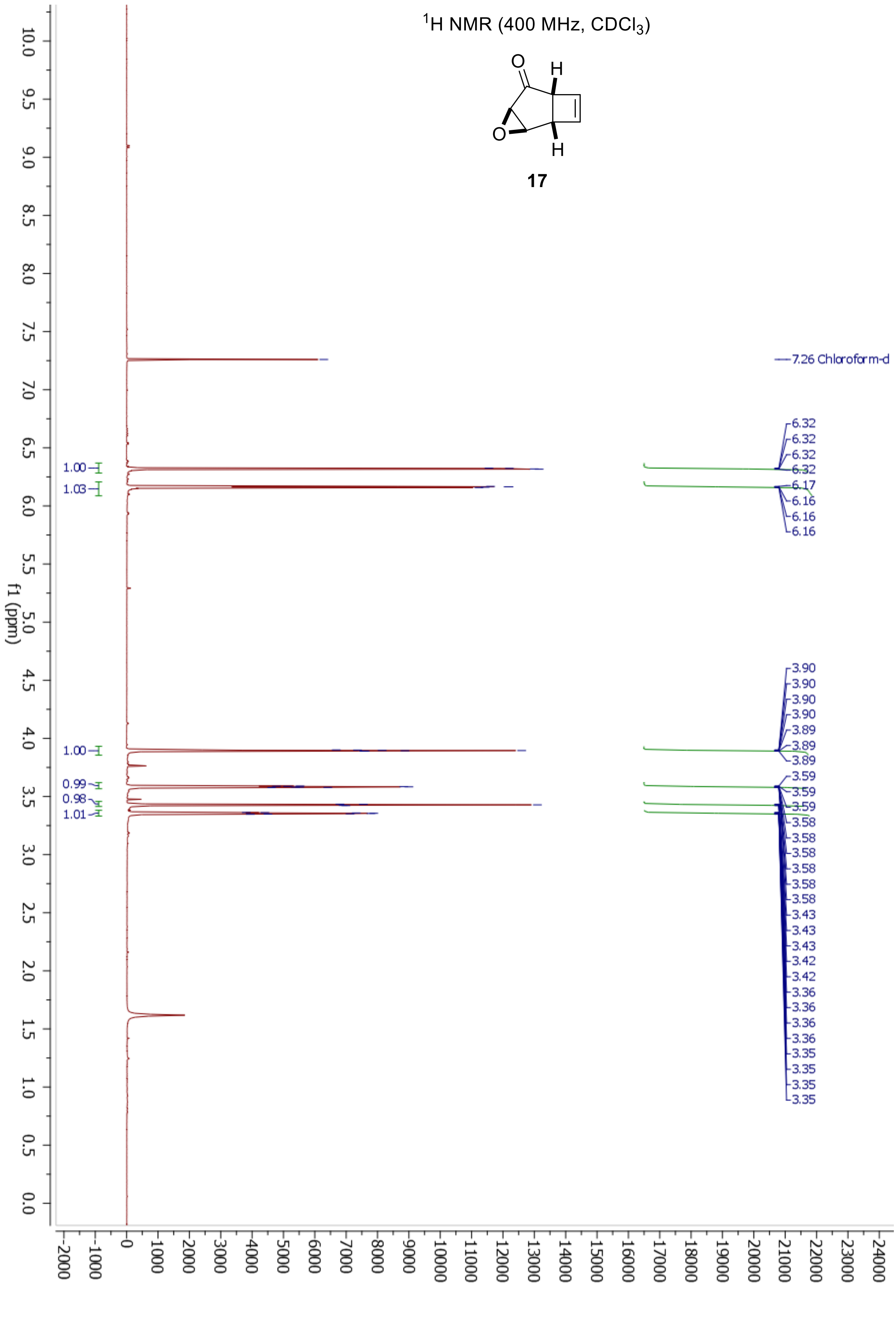

1H NMR (400 MHz, CDCl3)
17
7.26 Chloroform-d
6.32
6.32
6.32
6.32
6.17
6.16
6.16
6.16
3.90
3.90
3.90
3.90
3.89
3.89
3.89
3.59
3.59
3.59
3.58
3.58
3.58
3.58
3.58
3.58
3.43
3.43
3.43
3.42
3.42
3.36
3.36
3.36
3.36
3.35
3.35
3.35
3.35
1.00
1.03
1.00
0.99
0.98
1.01
f1 (ppm)

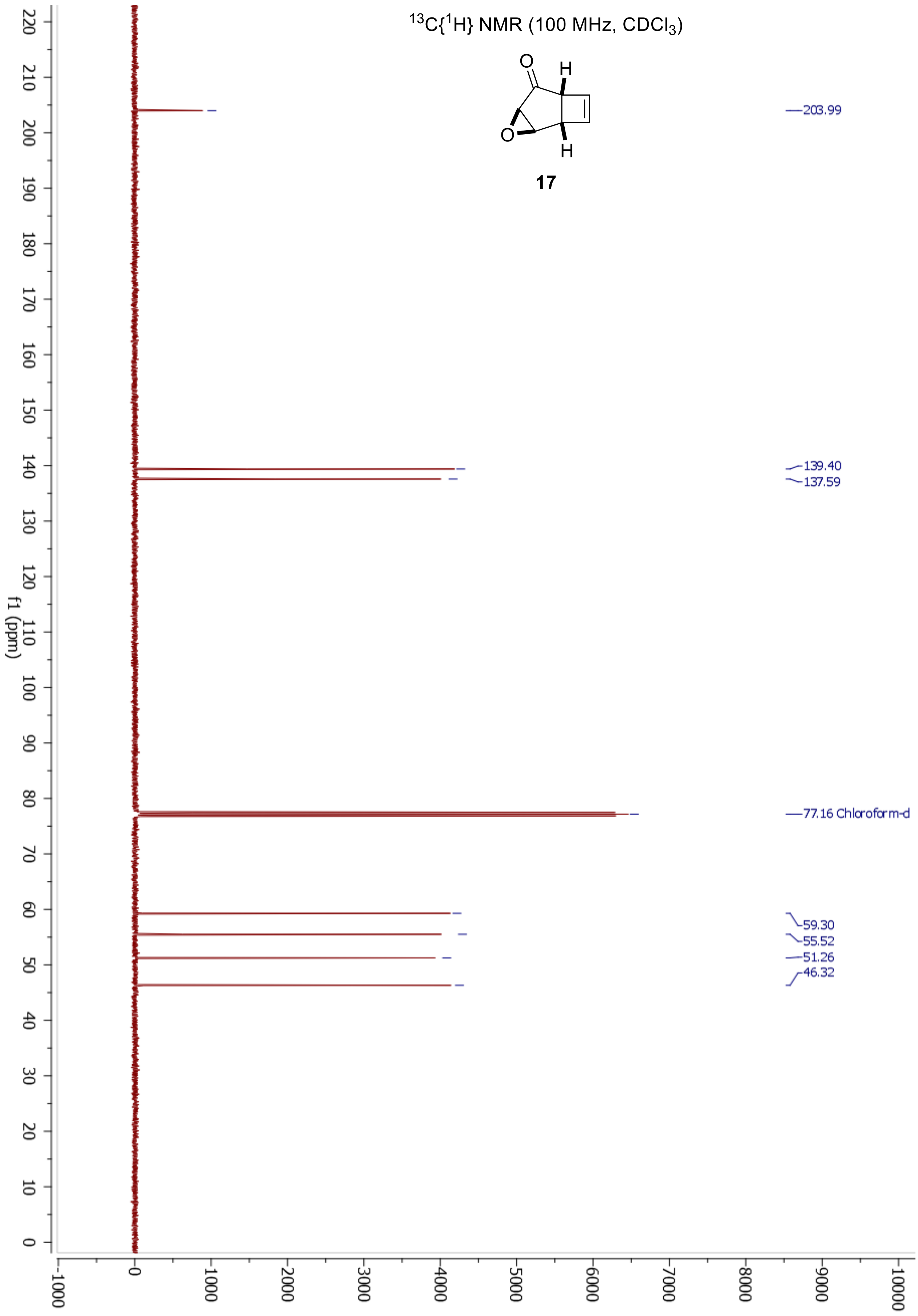
$^{13}C\{^1H\}$ NMR (100 MHz, $CDCl_3$)
17
203.99
139.40
137.59
77.16 Chloroform-d
59.30
55.52
51.26
46.32
f1 (ppm)

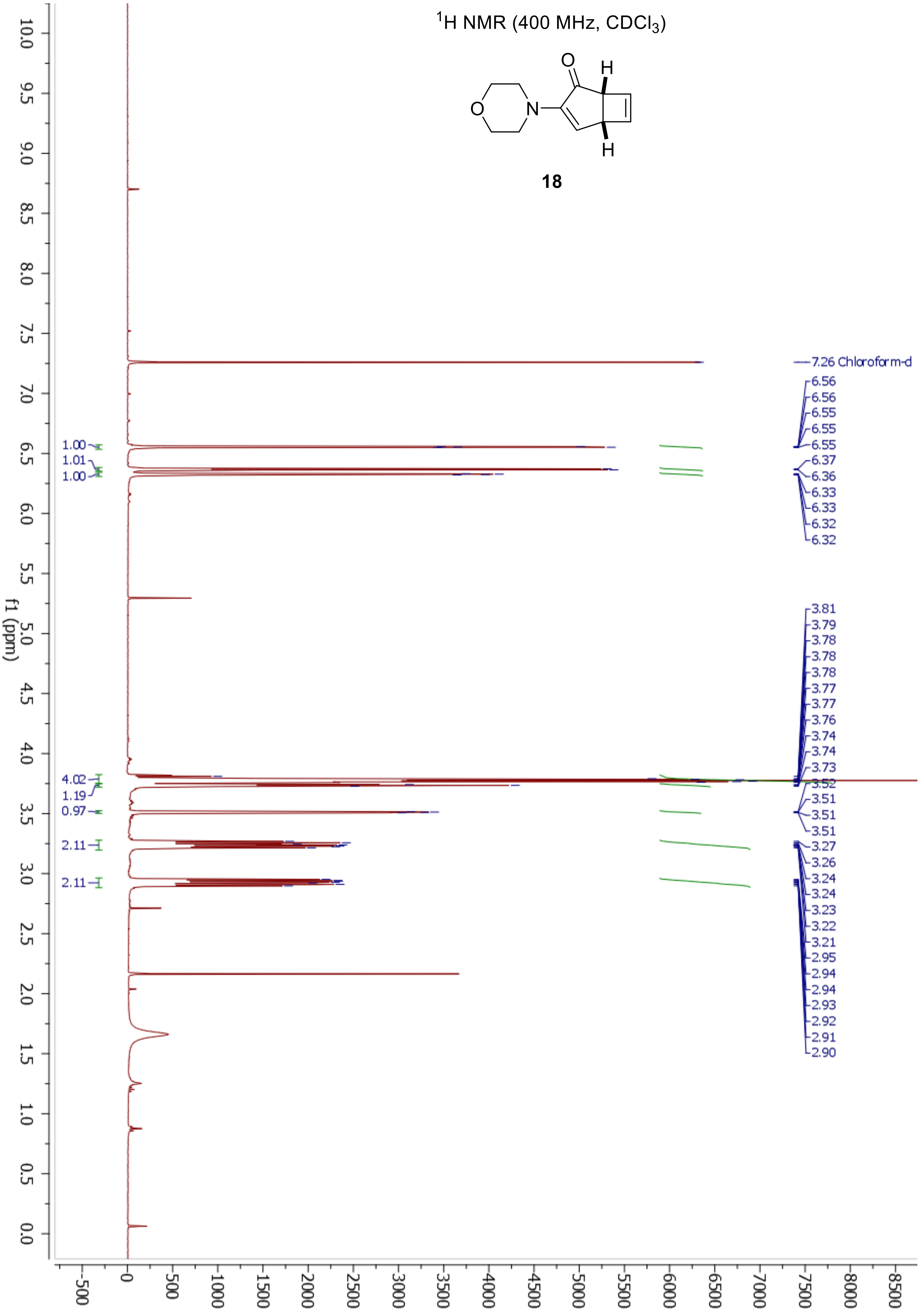
^{1}H NMR (400 MHz, $CDCl_3$)
O
N
H
18
7.26 Chloroform-d
6.56
6.56
6.55
6.55
6.55
6.37
6.36
6.33
6.33
6.32
6.32
3.81
3.79
3.78
3.78
3.78
3.77
3.77
3.76
3.74
3.74
3.73
3.52
3.51
3.51
3.51
3.27
3.26
3.24
3.24
3.23
3.22
3.21
2.95
2.94
2.94
2.93
2.92
2.91
2.90
1.00
1.01
1.00
4.02
1.19
0.97
2.11
2.11
f1 (ppm)

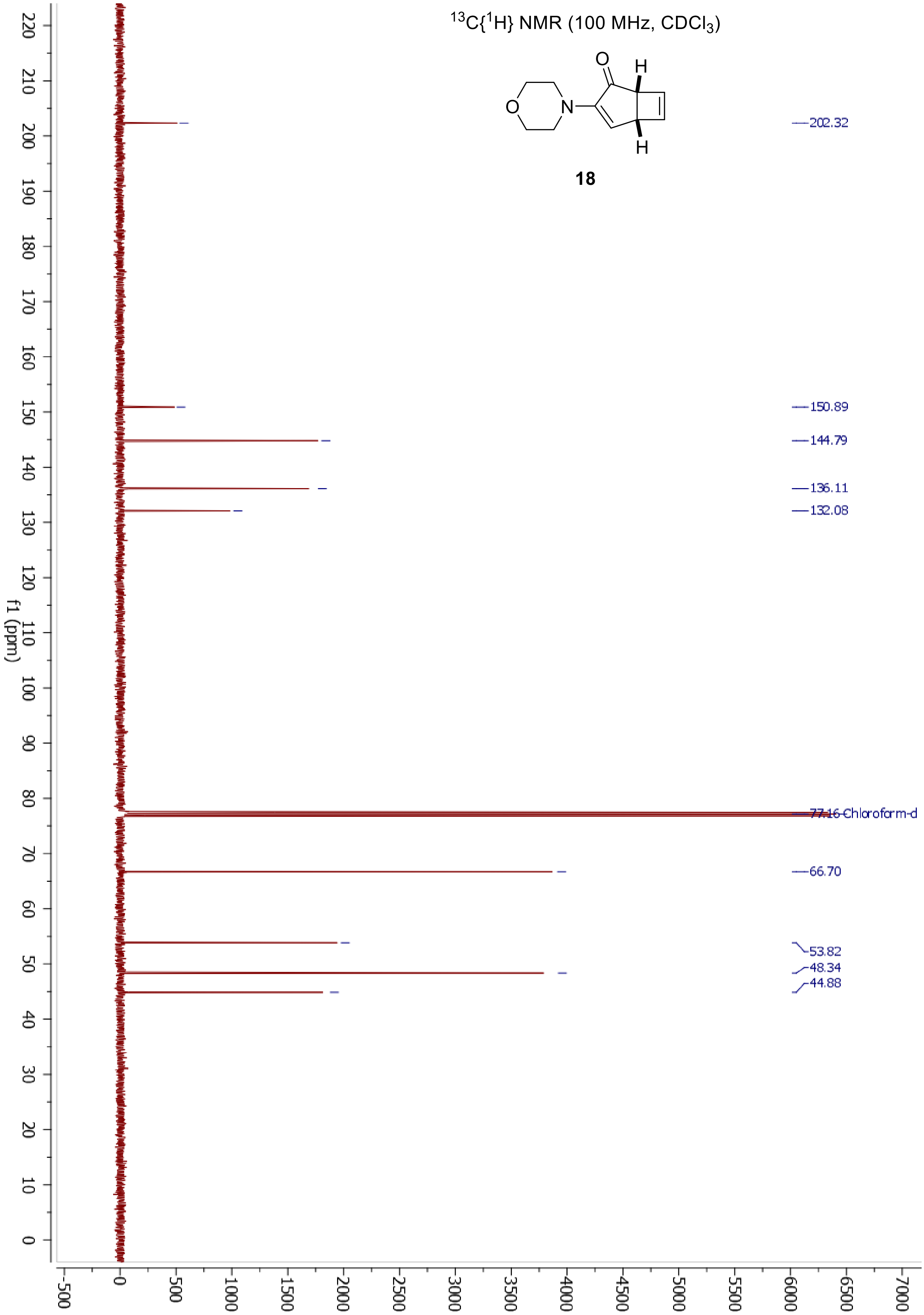
13C{1H} NMR (100 MHz, CDCl3)
O
N
O
H
H
18
202.32
150.89
144.79
136.11
132.08
77.16 Chloroform-d
66.70
53.82
48.34
44.88
f1 (ppm)

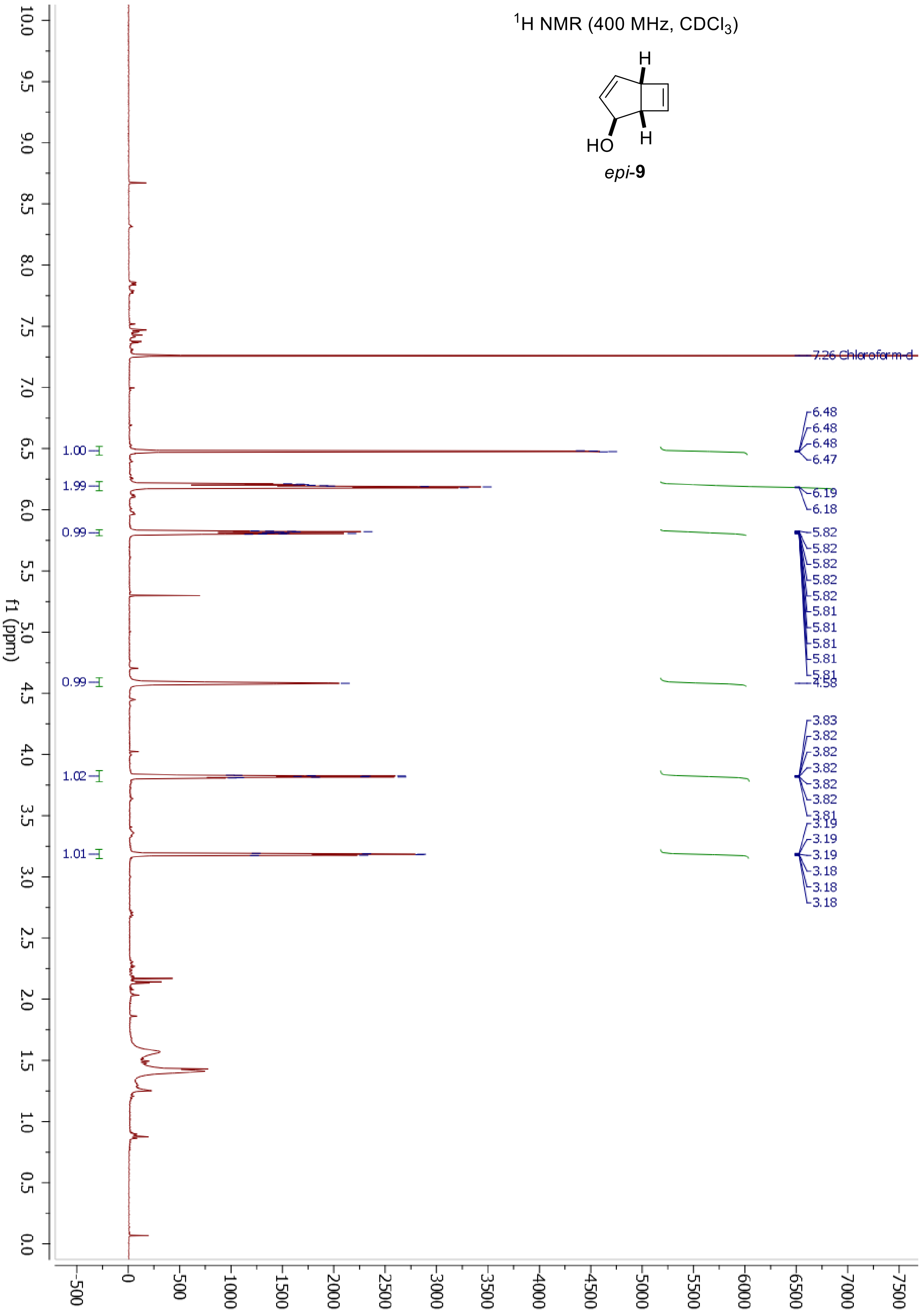

^{1}H NMR (400 MHz, $CDCl_3$)
H
H
HO
epi-**9**
7.26 Chloroform-d
6.48
6.48
6.48
6.47
6.19
6.18
5.82
5.82
5.82
5.82
5.82
5.81
5.81
5.81
5.81
5.81
4.58
3.83
3.82
3.82
3.82
3.82
3.82
3.81
3.19
3.19
3.19
3.18
3.18
3.18
1.00
1.99
0.99
0.99
1.02
1.01
f1 (ppm)

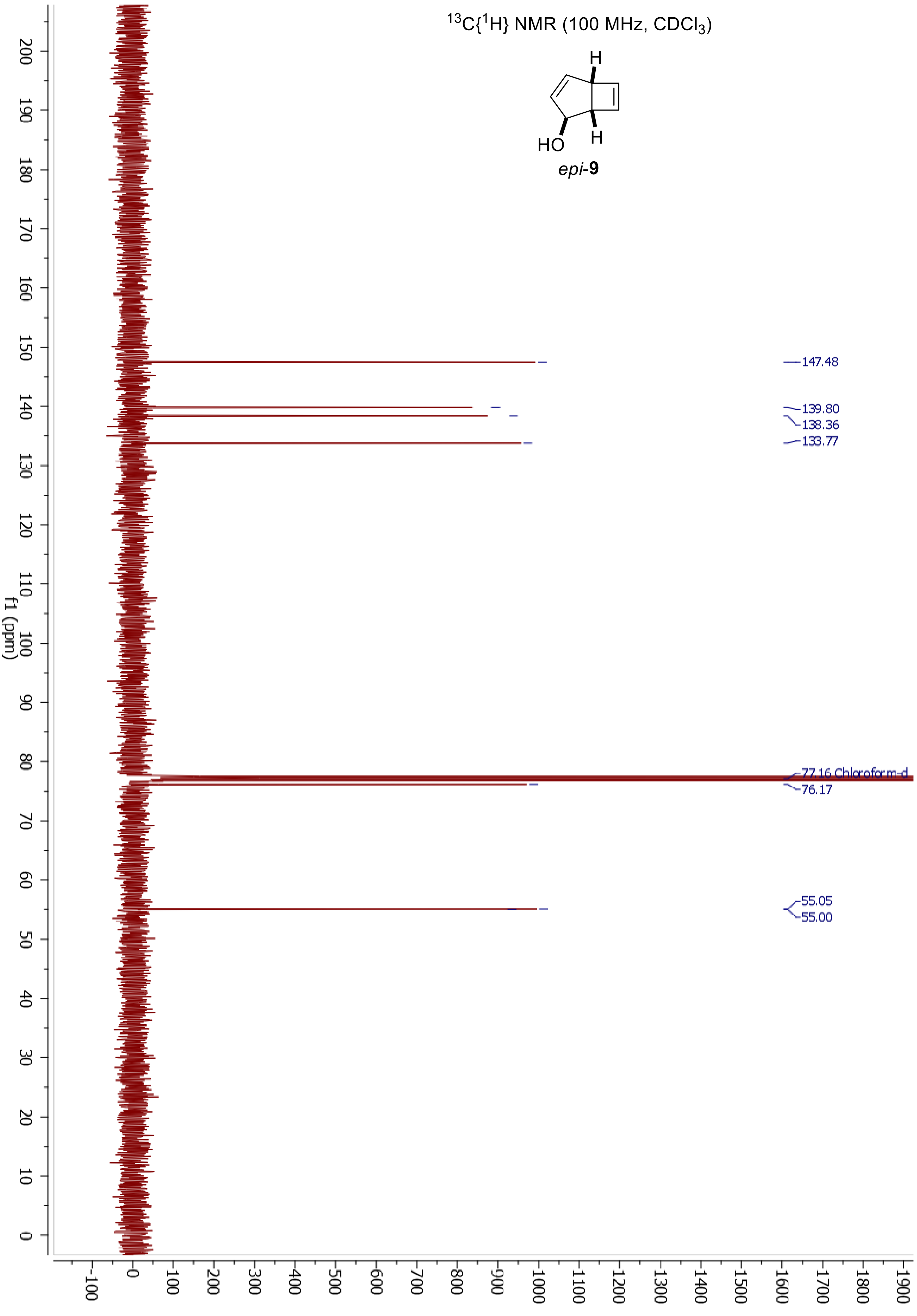

$^{13}C\{^{1}H\}$ NMR (100 MHz, $CDCl_3$)
HO
H
H
epi-**9**
147.48
139.80
138.36
133.77
77.16 Chloroform-d
76.17
55.05
55.00
f1 (ppm)

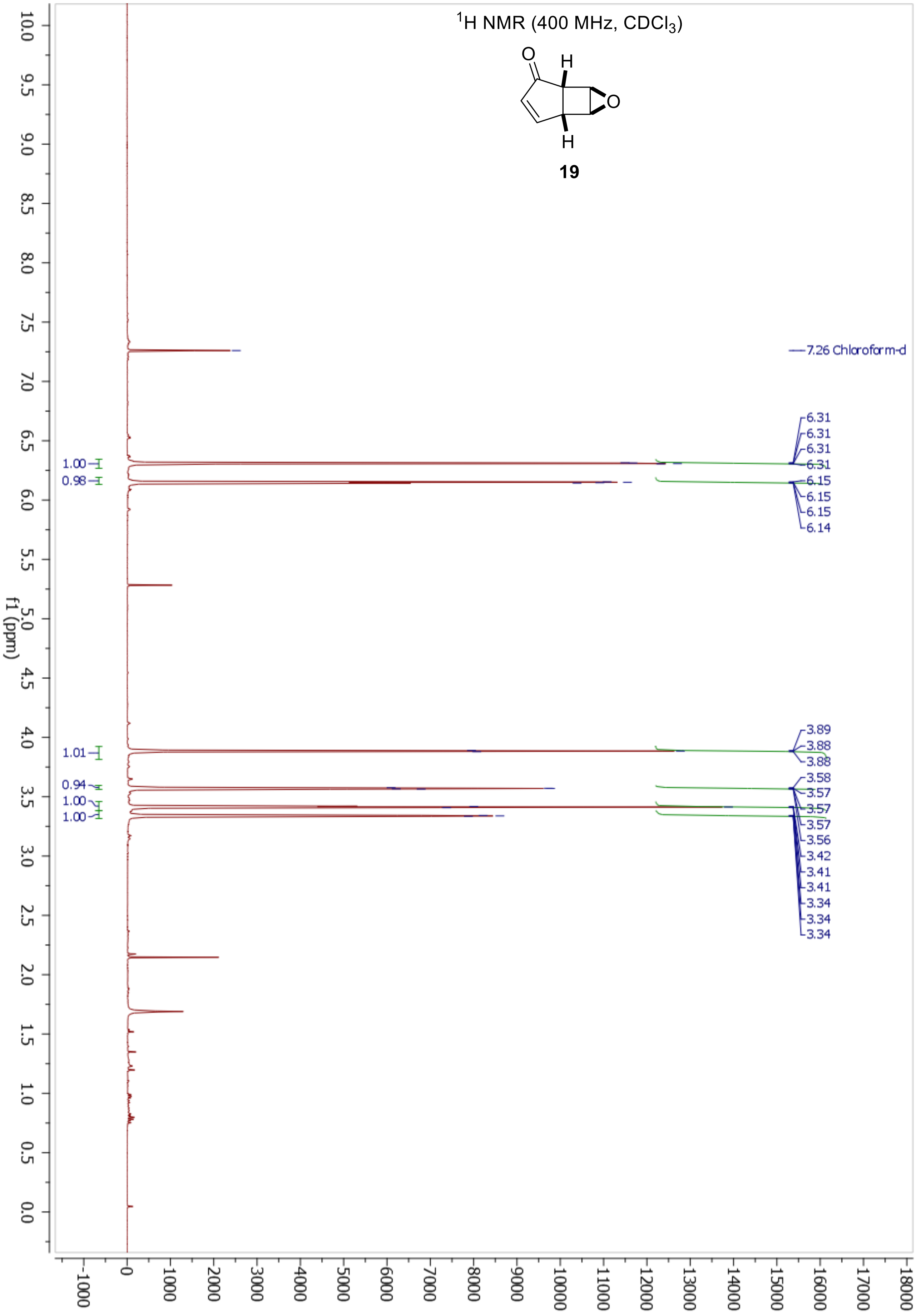

1H NMR (400 MHz, CDCl3)
O
H
O
H
19
7.26 Chloroform-d
6.31
6.31
6.31
6.31
6.15
6.15
6.15
6.14
3.89
3.88
3.88
3.58
3.57
3.57
3.57
3.56
3.42
3.41
3.41
3.34
3.34
3.34
1.00
0.98
1.01
0.94
1.00
1.00
f1 (ppm)

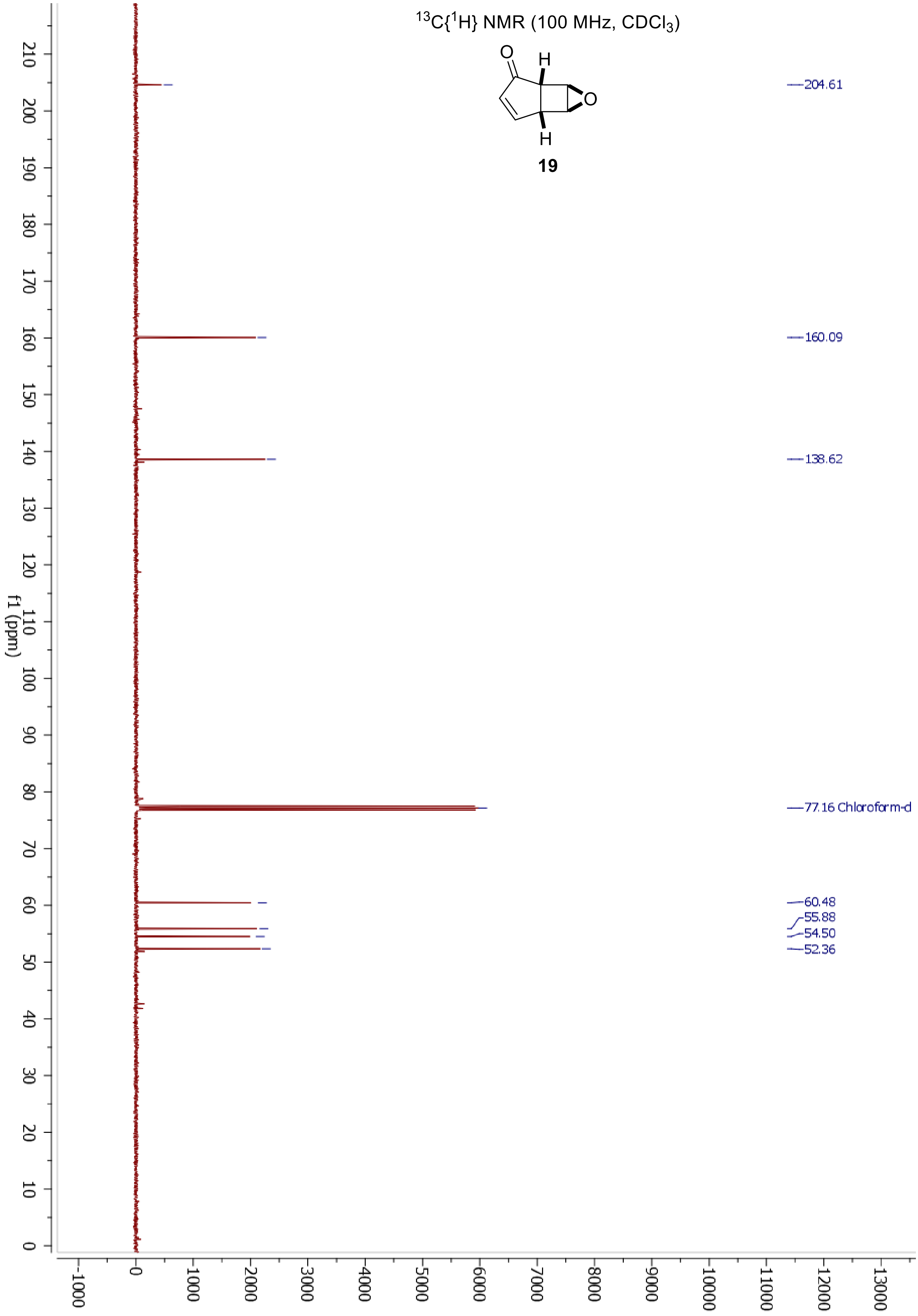
$^{13}C\{^1H\}$ NMR (100 MHz, $CDCl_3$)
19
204.61
160.09
138.62
77.16 Chloroform-d
60.48
55.88
54.50
52.36
f1 (ppm)

^1H NMR (400 MHz, $CDCl_3$)

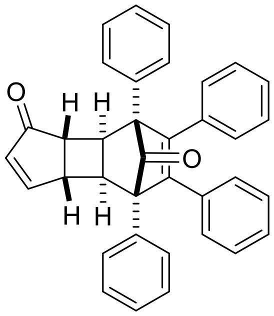

20

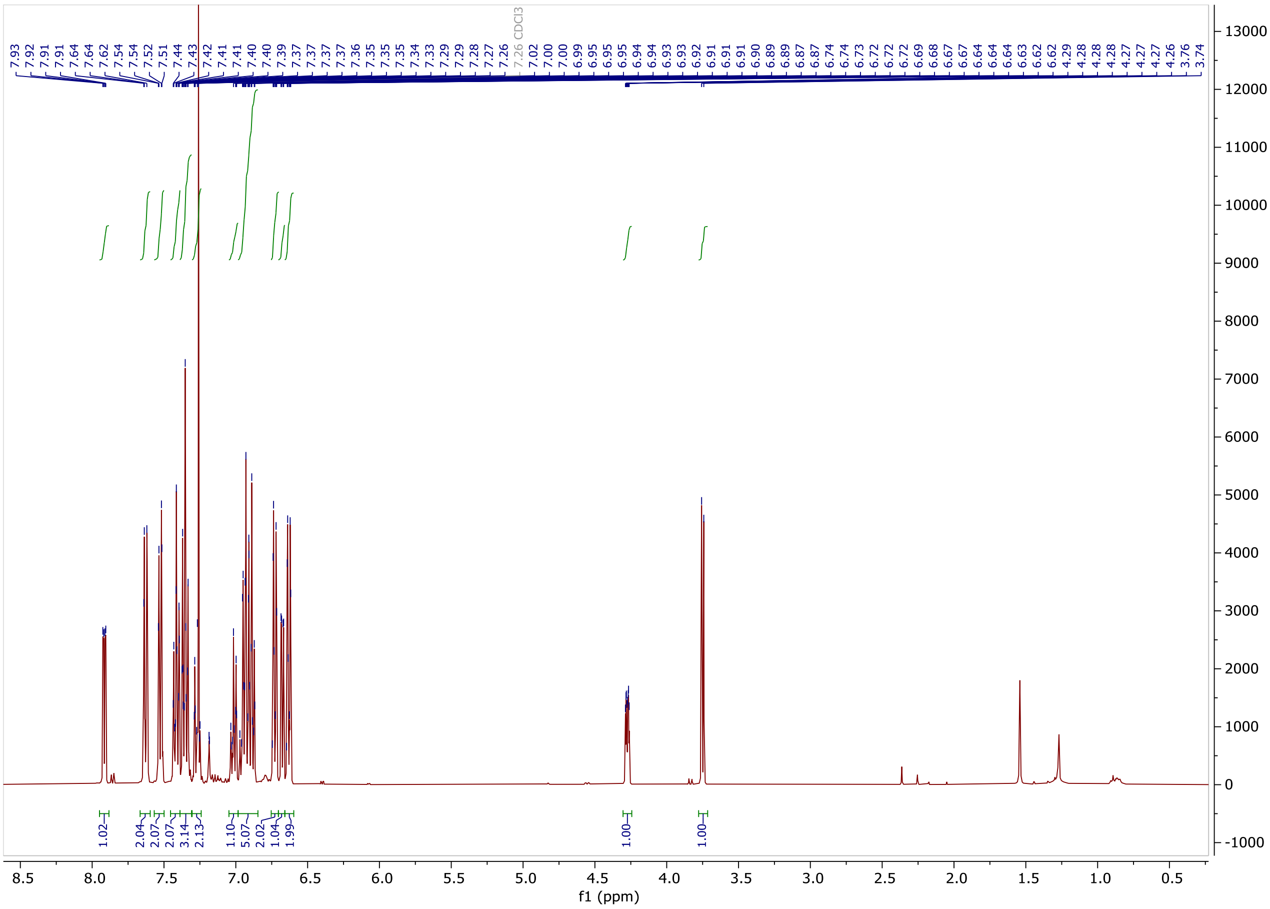

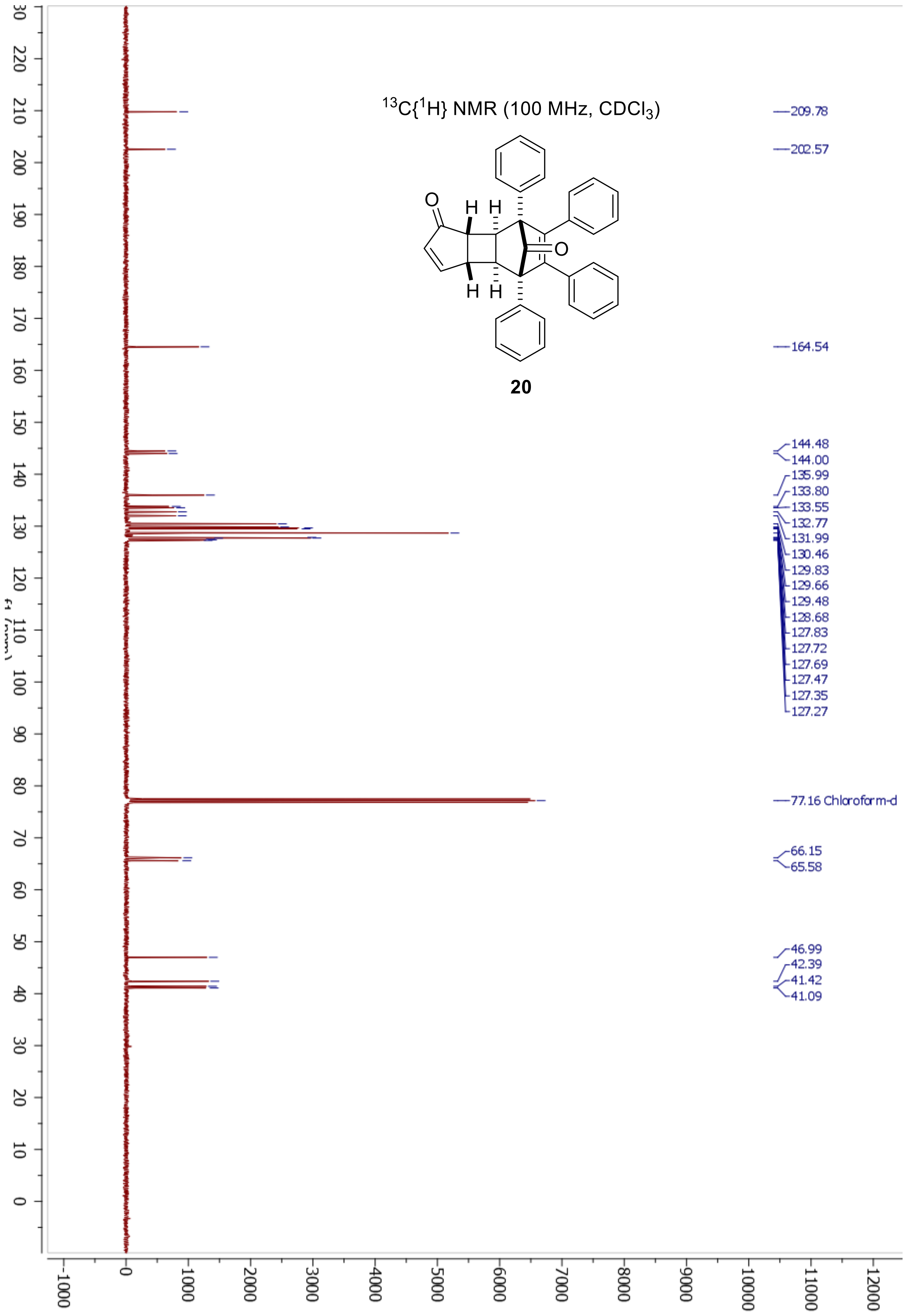

$^{13}C\{^1H\}$ NMR (100 MHz, $CDCl_3$)
20
209.78
202.57
164.54
144.48
144.00
135.99
133.80
133.55
132.77
131.99
130.46
129.83
129.66
129.48
128.68
127.83
127.72
127.69
127.47
127.35
127.27
77.16 Chloroform-d
66.15
65.58
46.99
42.39
41.42
41.09

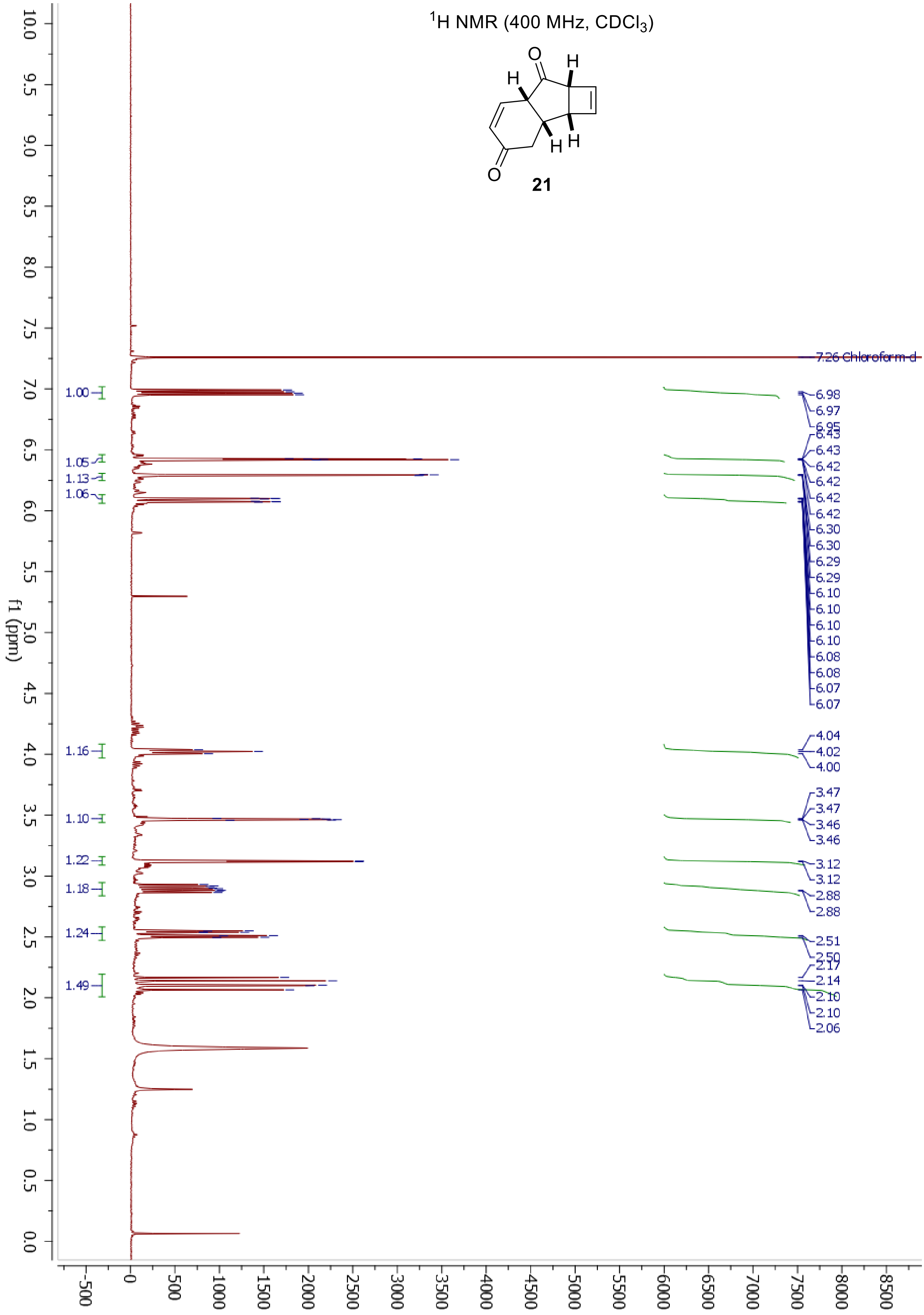

^{1}H NMR (400 MHz, CDCl$_3$)
21
7.26 Chloroform-d
6.98
6.97
6.95
6.43
6.43
6.42
6.42
6.42
6.42
6.30
6.30
6.29
6.29
6.10
6.10
6.10
6.10
6.08
6.08
6.07
6.07
4.04
4.02
4.00
3.47
3.47
3.46
3.46
3.12
3.12
2.88
2.88
2.51
2.50
2.17
2.14
2.10
2.10
2.06
1.00
1.05
1.13
1.06
1.16
1.10
1.22
1.18
1.24
1.49
f1 (ppm)

$^{13}C\{^1H\}$ NMR (100 MHz, $CDCl_3$)

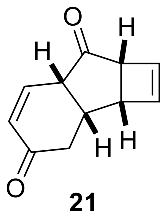

21

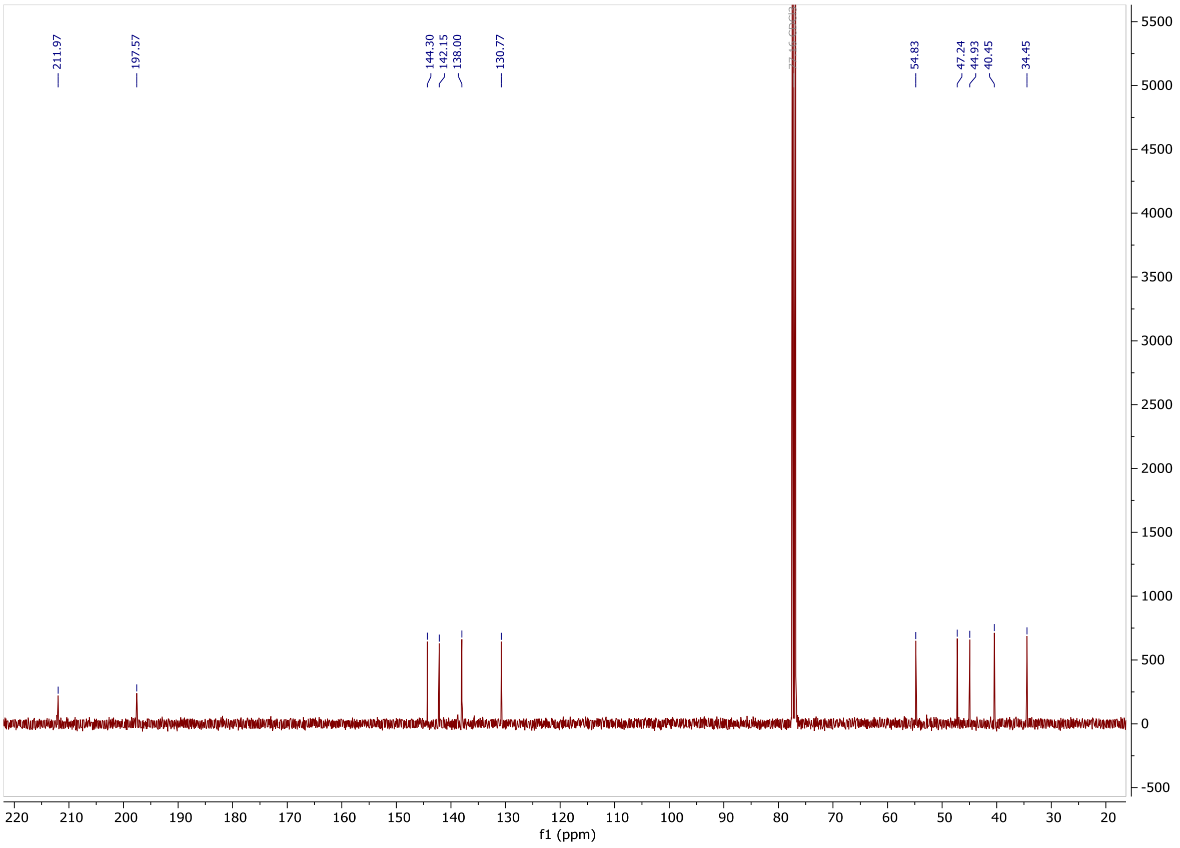

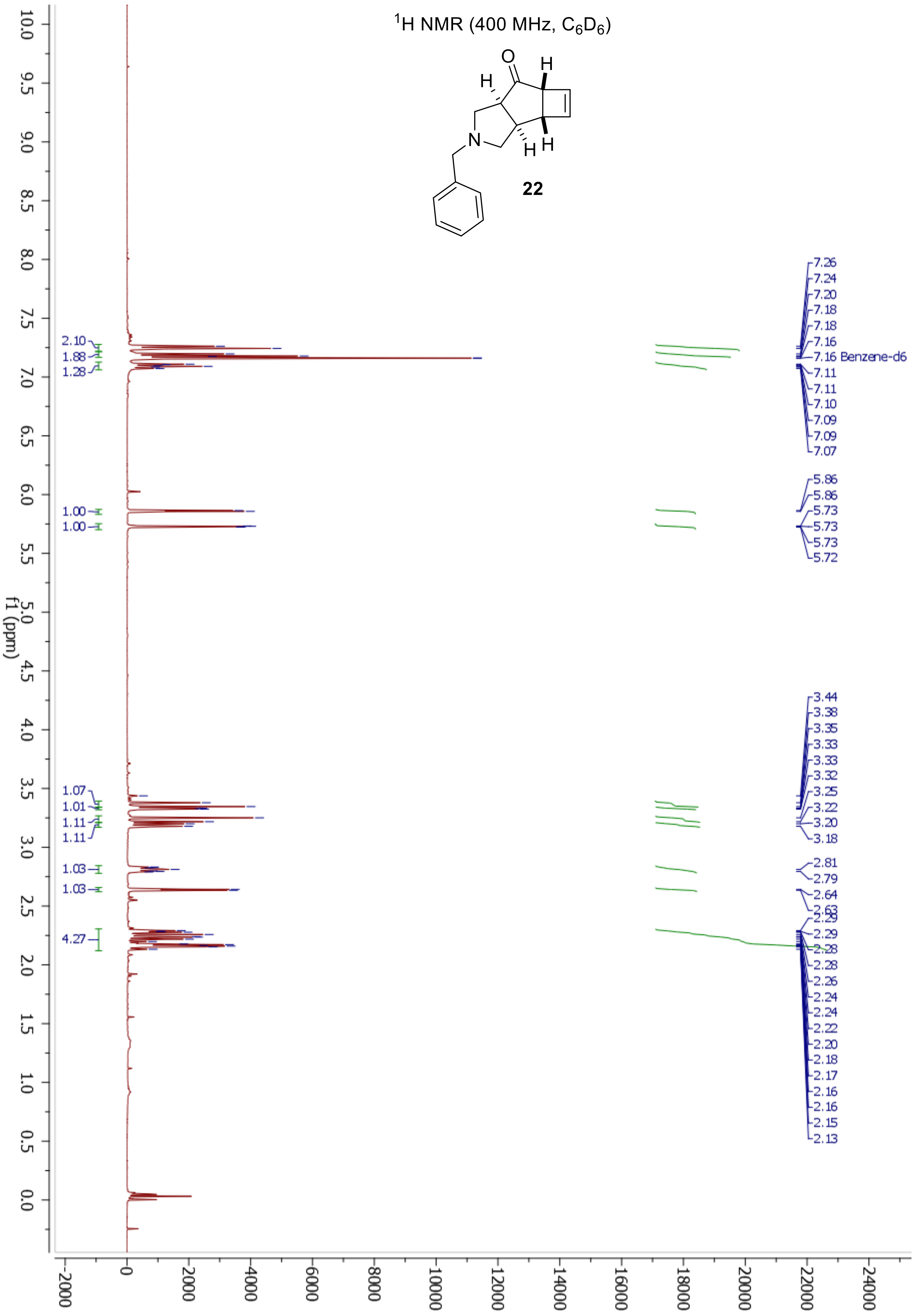

^{1}H NMR (400 MHz, C_6D_6)
22
f1 (ppm)
7.16 Benzene-d6

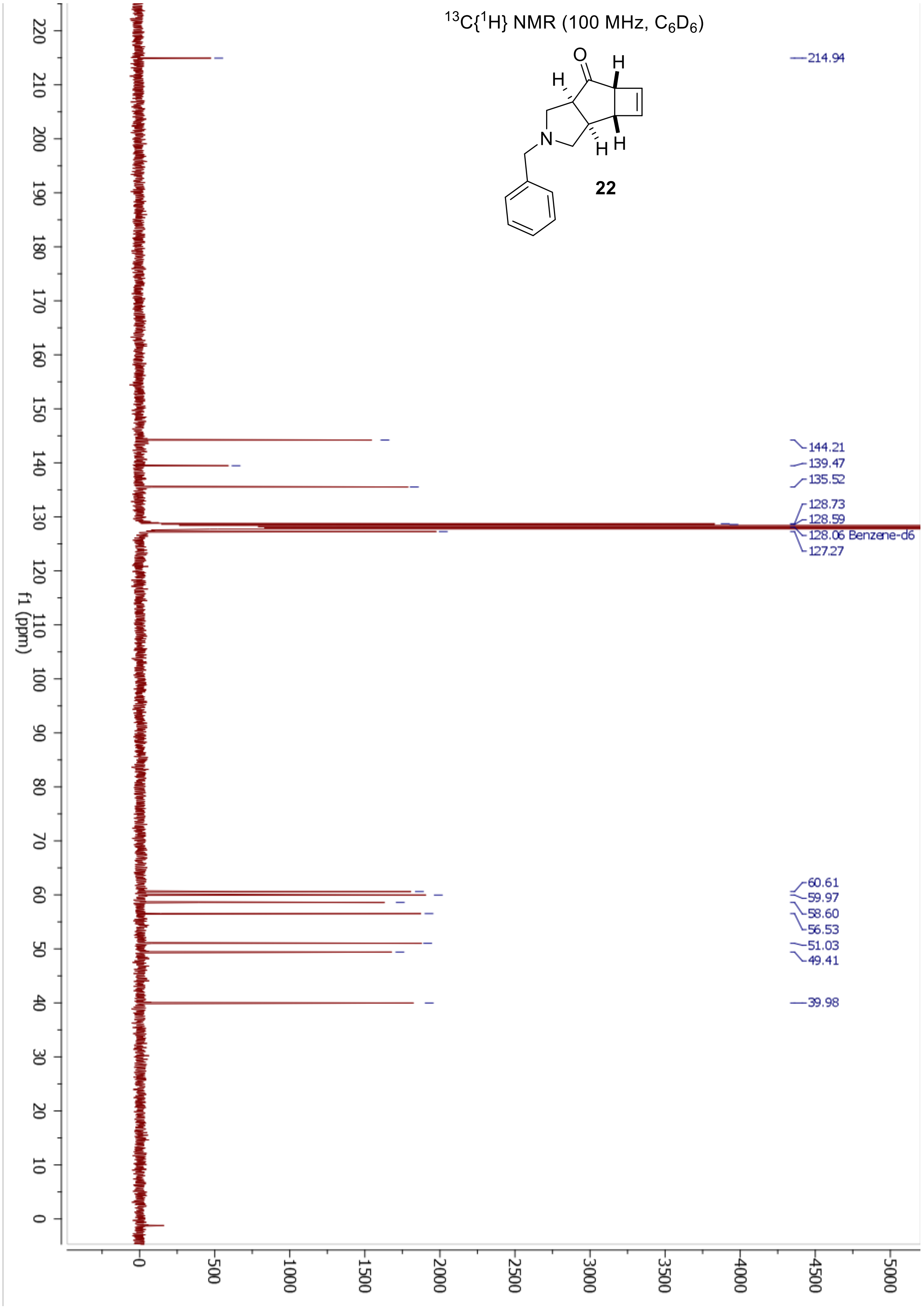

13C{1H} NMR (100 MHz, C6D6)
22
214.94
144.21
139.47
135.52
128.73
128.59
128.06 Benzene-d6
127.27
60.61
59.97
58.60
56.53
51.03
49.41
39.98
f1 (ppm)

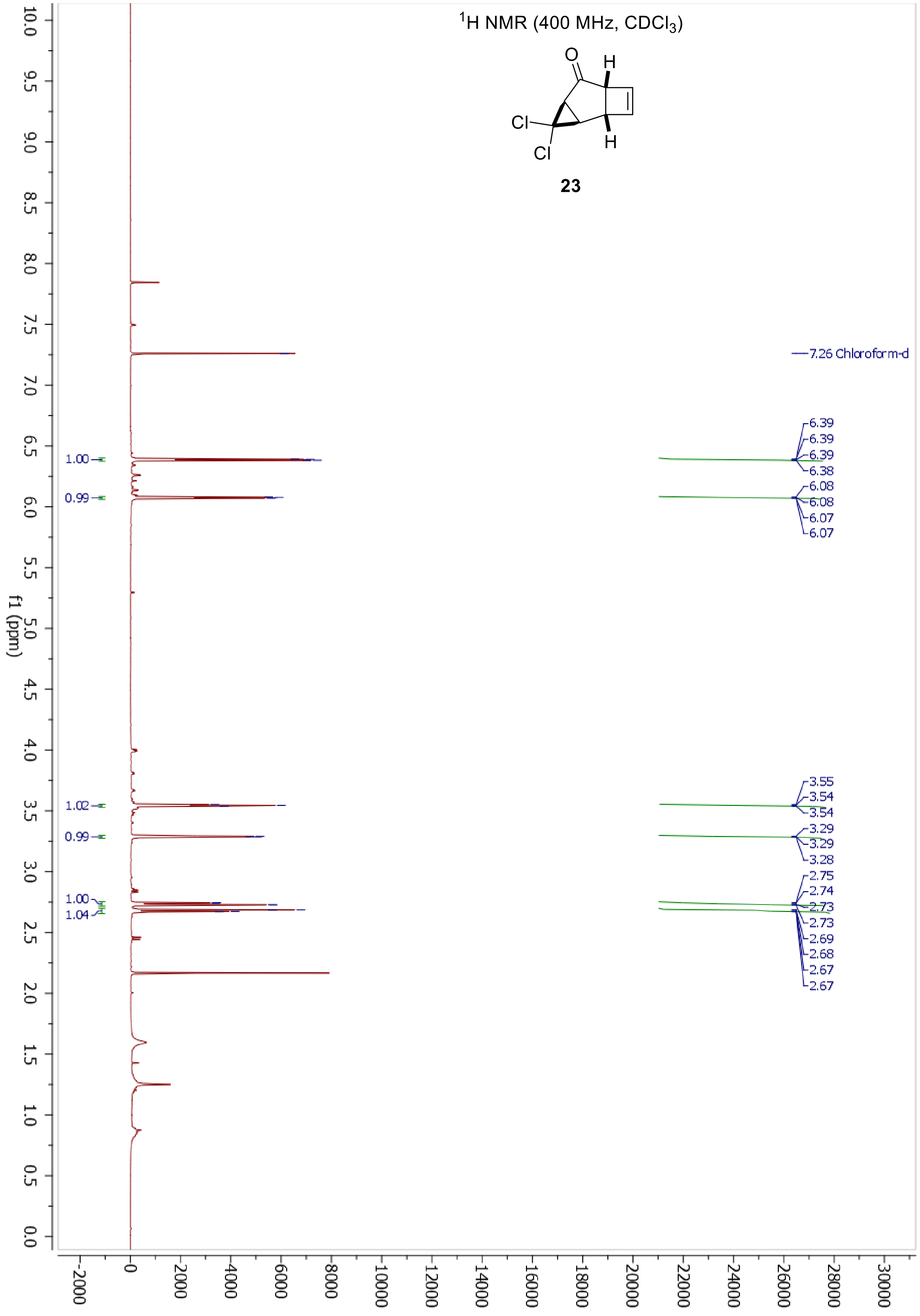

^{1}H NMR (400 MHz, $CDCl_3$)
O
H
Cl
Cl
H
23
7.26 Chloroform-d
6.39
6.39
6.39
6.38
6.08
6.08
6.07
6.07
3.55
3.54
3.54
3.29
3.29
3.28
2.75
2.74
2.73
2.73
2.69
2.68
2.67
2.67
1.00
0.99
1.02
0.99
1.00
1.04
f1 (ppm)

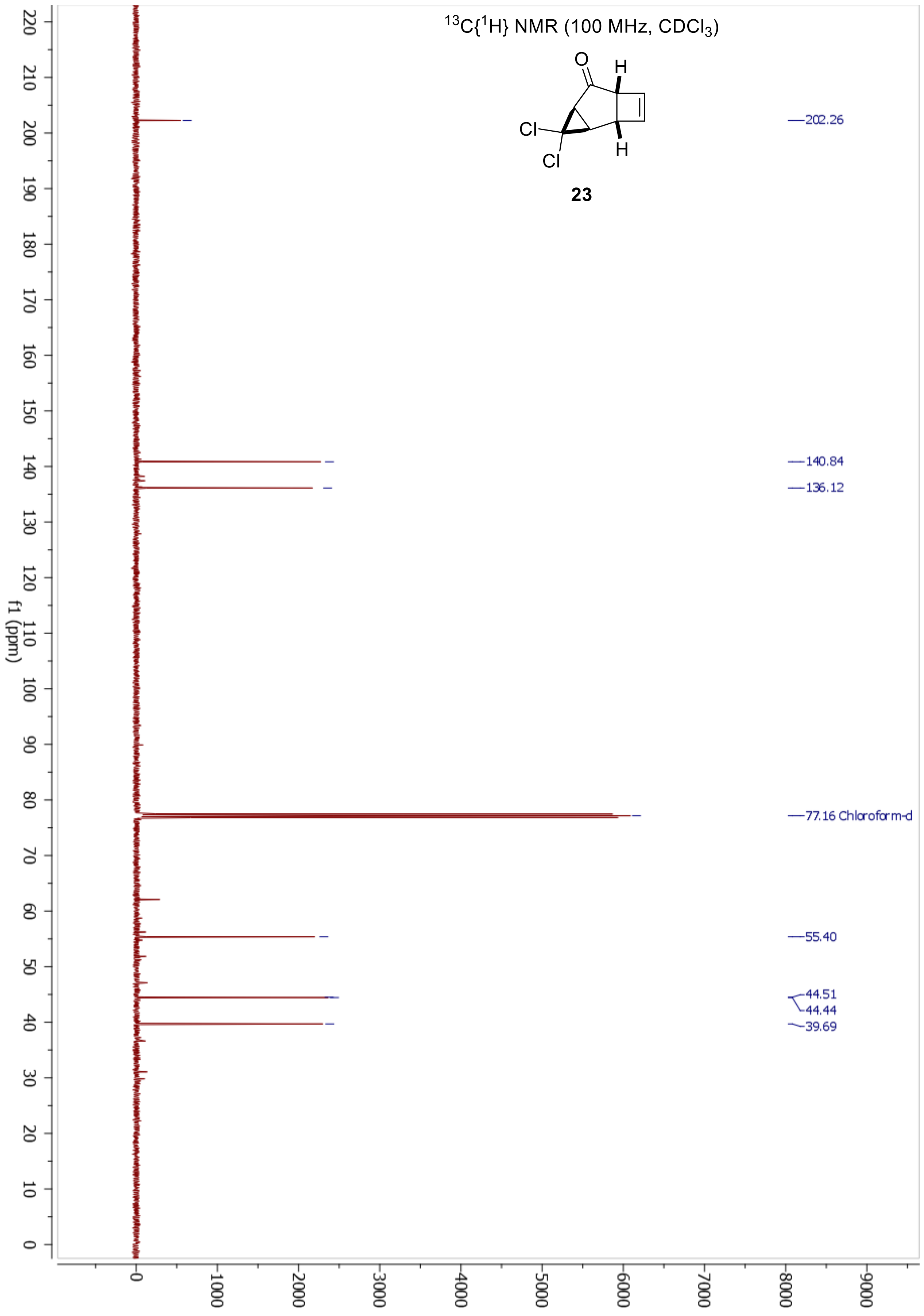
$^{13}C\{^1H\}$ NMR (100 MHz, $CDCl_3$)
O
H
Cl
Cl
H
23
202.26
140.84
136.12
77.16 Chloroform-d
55.40
44.51
44.44
39.69
f1 (ppm)
